# Tryptophanol-Derived Oxazolopyrrolidone Lactams as Potential Anticancer Agents against Gastric Adenocarcinoma

**DOI:** 10.3390/ph14030208

**Published:** 2021-03-02

**Authors:** Margarida Espadinha, Valentina Barcherini, Lídia M. Gonçalves, Elies Molins, Alexandra M. M. Antunes, Maria M. M. Santos

**Affiliations:** 1 Research Institute for Medicines (iMed.ULisboa), Faculty of Pharmacy, Universidade de Lisboa, Av. Prof. Gama Pinto, 1649-003 Lisboa, Portugal; mespadinha@ff.ulisboa.pt (M.E.); vbarcherini@ff.ulisboa.pt (V.B.); lgoncalves@ff.ulisboa.pt (L.M.G.); 2 Institut de Ciència de Materials de Barcelona (ICMAB-CSIC), Campus UAB, 08193 Bellaterra, Spain; elies.molins@icmab.es; 3 Centro de Química Estrutural, Instituto Superior Técnico, ULisboa, 1049-001 Lisboa, Portugal; alexandra.antunes@tecnico.ulisboa.pt

**Keywords:** anticancer agents, cytotoxicity, enantioselective synthesis, gastric adenocarcinoma, tryptophanol

## Abstract

Gastric cancer is one of the deadliest cancers in modern societies, so there is a high level of interest in discovering new drugs for this malignancy. Previously, we demonstrated the ability of tryptophanol-derived polycyclic compounds to activate the tumor suppressor protein p53, a relevant therapeutic target in cancer. In this work, we developed a novel series of enantiomerically pure tryptophanol-derived small molecules to target human gastric adenocarcinoma (AGS) cells. From an initial screening of fourteen compounds in AGS cell line, a hit compound was selected for optimization, leading to two derivatives selective for AGS gastric cells over other types of cancer cells (MDA-MB-231, A-549, DU-145, and MG-63). More importantly, the compounds were non-toxic in normal cells (HEK 293T). Additionally, we show that the growth inhibition of AGS cells induced by these compounds is mediated by apoptosis. Stability studies in human plasma and human liver microsomes indicate that the compounds are stable, and that the major metabolic transformations of these molecules are mono- and di-hydroxylation of the indole ring.

## 1. Introduction

**Figure 1:**
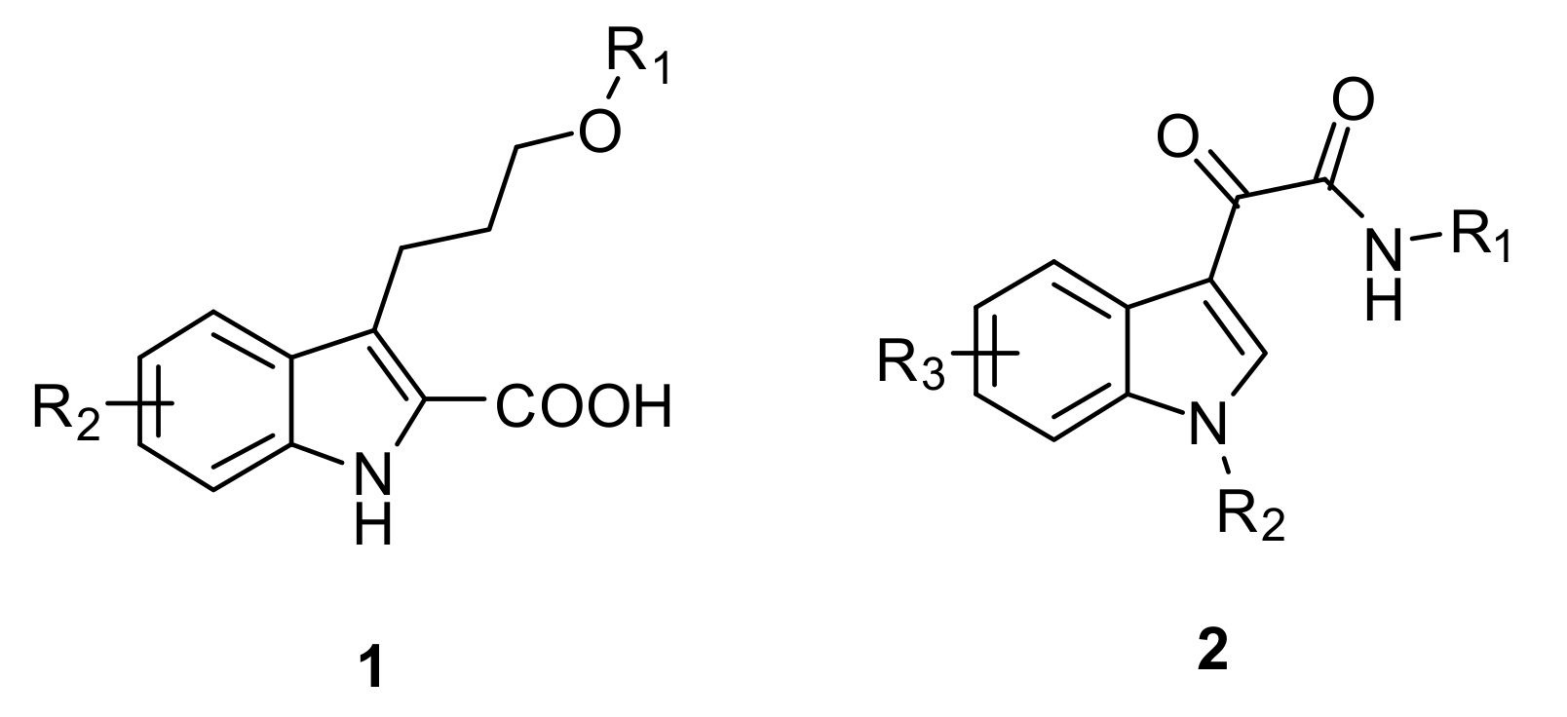
Chemical structures of indole-derived compounds with in vitro anticancer activity: Mcl-1 inhibitors (**1**) and tubulin inhibitors (**2**).

Cancer is considered a worldwide health problem, and its occurrence can be associated to a combination of environmental factors and genetic alterations [1]. According to the World Health Organization (WHO), it is estimated that in 2018, cancer contributed to 9.5 million deaths worldwide [2]. Gastric cancer (GC) ranks third in the list of deadliest cancers [1], and its occurrence and mortality are highly influenced by region and culture [3]. The survival rate of GC has not improved much over the last years. Patients with GC in early-stage, usually, do not have symptoms, which hinders the early detection of this cancer. For this reason, most patients present advanced GC and, in these cases, radical surgery is the first-line approach and the only curative treatment [4]. In the cases that surgery is not recommended, alternative treatments can be used, such as chemotherapy, radiotherapy, and immunotherapy. However, these therapeutic options only achieve modest results, and the poor response of this cancer to chemotherapy is, typically, associated to chemoresistance mechanisms [5,6]. Moreover, the severe side effects associated to drug-related toxicity are frequent [7,8]. Consequently, the discovery of new alternative therapeutics for the treatment of GC, with low cost and minimal side effects, is still urgently needed. In the last decades, the discovery of cellular mechanisms associated to malignancies has been intensive, and many anticancer agents were developed to disrupt specific biological pathways. With this, the discovery of new scaffolds increased, as well as the interest in new therapeutic applications to scaffolds already known. For example, the indole scaffold is associated to many pharmacological activities in medicinal chemistry, including antimicrobial, antioxidant, antiviral, and anticancer [9,10]. It is considered a privileged scaffold, commonly found in many natural products (e.g., alkaloids and microbial hormones) and synthetic molecules with medicinal value (e.g., compounds **1** and **2**, Figure 1) [11].

**Figure 2:**
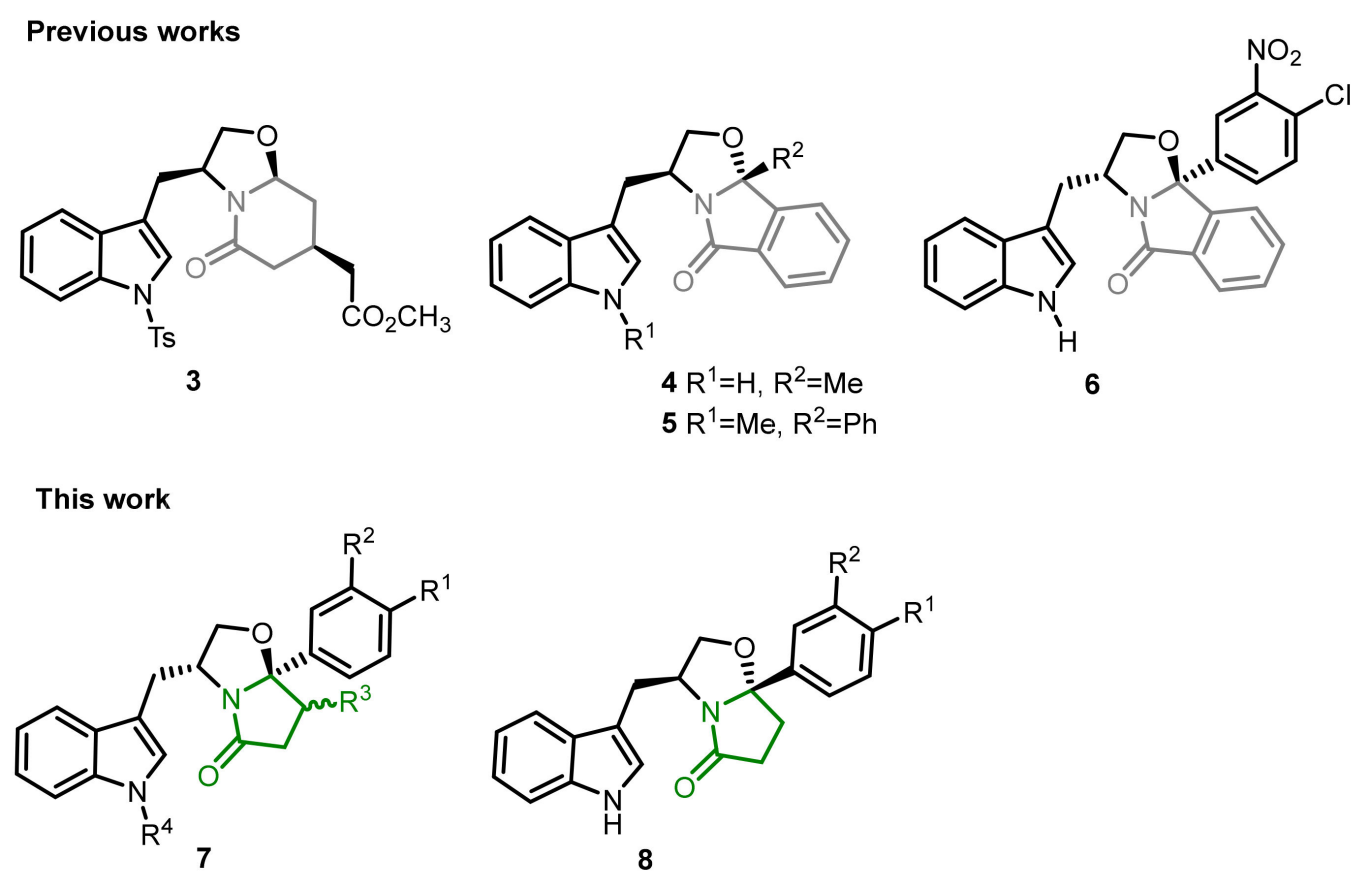
Previously reported tryptophanol-based scaffolds **3**–**6** and tryptophanol-derived oxazolopyrrolidones **7**–**8** studied in this work.

Other examples are tryptophanol-based small molecules (e.g., compounds **3**–**6**, Figure 2), reactivators of the p53 pathway, that showed in vitro antiproliferative activity in colon and breast cancer cells [12,13,14,15,16,17]. Specifically, tryptophanol-derived isoindolinones **4**–**5** presented promising in vivo antitumor results in xenograft mouse models, without cytotoxicity and genotoxicity [13,14,16]. Based on these results, and on reported results with pyrrolidone-based small molecules with anticancer activity [18,19], we envisioned that the merge of these two scaffolds could lead to compounds with interesting anticancer properties [15]. Herein, we report the synthesis of 29 enantiopure tryptophanol-derived oxazolopyrrolidone lactams (compounds **7** and **8**, Figure 2), their antiproliferative activity in human gastric adenocarcinoma (AGS) cell line, and in vitro stability and metabolic studies with this scaffold.

## 2. Results and Discussion

### 2.1. Chemistry

**Scheme 1:**
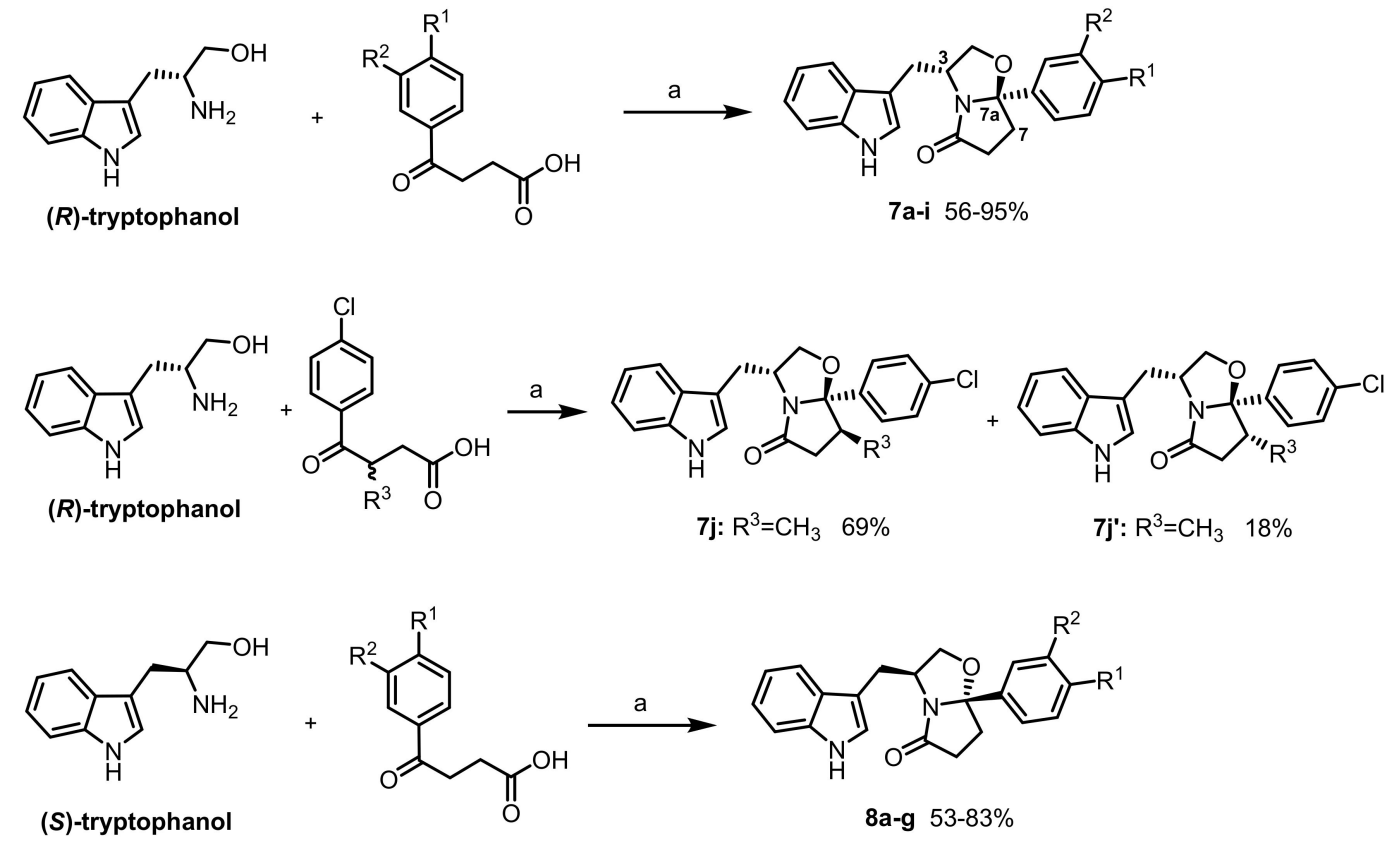
Synthesis of (*R*)- and (*S*)-tryptophanol-derived oxazolopyrrolidone lactams **7a**–**j**, **7j’**, and **8a**–**g**, respectively. Reaction conditions: (a) toluene, reflux, 10–25 h.

Enantiopure bicyclic lactams **7a**–**j**, **7j’**, and **8a**–**g** were easily accessed by a chiral-induced cyclocondensation reaction, starting from enantiopure tryptophanol and commercially available keto acids, in low to excellent yields (18–95%, Scheme 1) [20]. In almost all reactions, the formation of only one diastereomer was observed by thin-layer chromatography (TLC) and proton nuclear magnetic resonance (^1^H NMR). In the cyclocondensation reaction of (*R*)-tryptophanol with 4-(4-chlorophenyl)-3-methyl-4-oxobutanoic acid, in which an additional chirality center is formed, diastereomer **7j** (69% yield) was obtained, as well as the minor diastereoisomer **7j’** (18% yield).

**Scheme 2:**
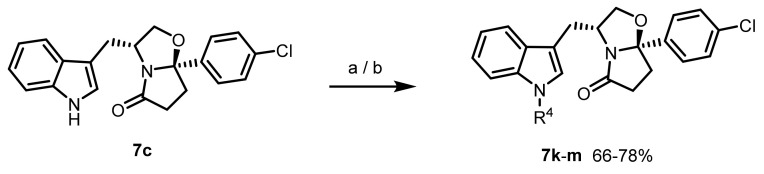
Synthesis of (*R*)-tryptophanol-derived oxazolopyrrolidone lactams **7k**–**m**. Reaction conditions: (a) compounds **7k**–**l**: ethyl iodide/acetic anhydride, NaH, DMF, 0 °C-r.t., 3–6 h; (b) compound **7m**: di-*tert*-butyl dicarbonate, DMAP, Et_3_N, THF, r.t., 3 h.

Tryptophanol-derived oxazolopyrrolidone lactams **7k**–**m**, with substituents on the indole nitrogen, were obtained in moderate to good yields (66–78%, Scheme 2). Specifically, compounds **7k**–**l** were synthesized by treatment of **7c** with sodium hydride in dimethylformamide, in the presence of iodoethane (compound **7k**) or acetic anhydride (compound **7l**). Compound **7m** was prepared by reaction of **7c** with di-*tert*-butyl dicarbonate, in the presence of 4-dimethylaminopyridine and triethylamine, in tetrahydrofuran. 

**Scheme 3:**
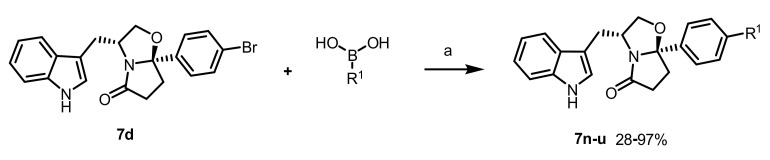
Synthesis of (*R*)-tryptophanol-derived oxazolopyrrolidone lactams **7n**–**u**. Reaction conditions: (a) Pd(PPh_3_)_2_Cl_2_, aq. sol. Na_2_CO_3_ (1 M), 1,4-dioxane, 100 °C, 2–5 h.

Compounds **7n**–**u** were obtained through Suzuki-Miyaura cross-coupling reaction between compound **7d** and different aryl boronic acids, using Pd(PPh_3_)_2_Cl_2_ as catalyst (Scheme 3). Except for compound **7u**, which was obtained in low yield (28%) due to the low solubility of the boronic acid, all the other derivatives were obtained in high yields (71–97%). 

**Figure 3:**
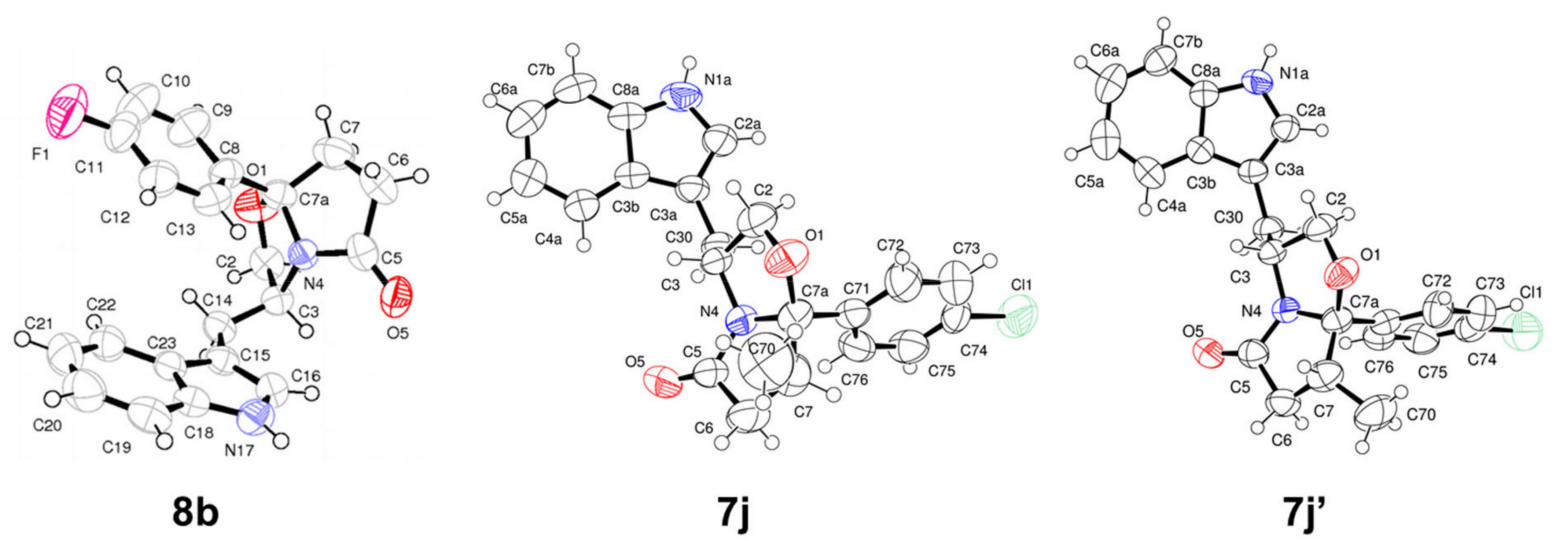
X-ray crystallographic structures of compounds **8b**, **7j**, and **7j’** (crystallographic information file (CIF) data can be found in the Supplementary Materials Tables S1–S15).

The absolute configuration of the new formed stereogenic center C-7a was established by X-ray analysis of compound **8b** (Figure 3). The ^13^C NMR spectroscopy data of compound **8b** was used as reference to confirm the stereochemistry of the other derivatives. For compounds **7a**–**i** and **8a**–**g**, the signals of C-3, C-7a, and C-7 appear between 55.5–56.5, 101.7–102.6, and 35.0–35.4 ppm, respectively. 

The spectral data obtained for compounds **7j** and **7j’** indicate that the major diastereomer **7j** has (3*R*, 7a*R*, 7*S*) configuration, while the minor diastereoisomer **7j’** has (3*R*, 7a*R*, 7*R*) configuration [21]. In particular, the methyl group appears in the ^1^H NMR spectra as a doublet at 1.12 ppm for **7j** and at 0.60 ppm for **7j’**, and in the ^13^C NMR spectra at 13.96 ppm for **7j** and at 16.40 ppm for **7j’**. Moreover, the methyl group induces a shift in the C-7 that appears at 39.7 ppm for compound **7j** and at 41.3 ppm for compound **7j’**. The chemical shift of C-3 appears in a higher field for diastereoisomer **7j’** (54.8 ppm). The absolute configuration of diastereomers **7j** and **7j’** was further confirmed by X-ray crystallography (Figure 3).

### 2.2. Effect of Tryptophanol-Derived Oxazolopyrrolidone Lactams on Cell Viability and on Apoptosis

To perform a structure–activity relationship (SAR) study, a first series of tryptophanol-derived oxazolopyrrolidone lactams containing different substituents on the phenyl ring (R^1^) at position C-7a was synthesized (compounds **7a**–**g** and **8a**–**g**, Table 1). In the design of this new compounds series, a diversity of substituents with electron donating properties (–CH_3_ and –OCH_3_ groups) and electron withdrawing properties (–F, –Cl, –Br, and –SO_2_CH_3_ groups) were chosen. Both series of enantiomers, (*S*)- and (*R*)-tryptophanol derivatives, were synthesized to evaluate the impact of compound’s stereochemistry on the antiproliferative response of AGS cells. The activity of the target compounds was assessed using the MTT reduction assay. In general, (*R*)-tryptophanol-derived oxazolopyrrolidone lactams were more active than the corresponding enantiomers, except for derivative **8b** with a *para*-fluoro substituent (**7a**–**g** vs. **8a**–**g**). From the first screening at 100 µM, analogues **7a** (R^1^ = H), **7b** (R^1^ = F), and **8e** (R^1^ = CH_3_) showed moderate antiproliferative activity, while compounds **7g** and **8g** (R^1^ = SO_2_CH_3_) did not induce appreciable cytotoxicity. Remarkably, compounds **7c**–**e** and **8c** revealed an antiproliferative response higher than 85%. The presence of chlorine or bromine substituents at R^1^ had a positive impact on the antiproliferative activity, for both enantiomers (compounds **7c**–**d** and **8c**–**d**). The derivative **7c** (R^1^ = Cl) exhibited the highest activity and was selected for chemical derivatizations to improve the antiproliferative activity of this scaffold in AGS cells.

Four sites were identified for suitable structural modifications in compound **7c**: *meta*- position of the C-7a phenyl ring (compounds **7h** and **7i**, Scheme 1), position C-7 of the pyrrolidone ring (compounds **7j** and **7j’**, Scheme 1), alkylation of the *N*-indole (compounds **7k**–**m**, Scheme 2) and C–C couplings in the C-7a phenyl ring (compounds **7n**–**u**, Scheme 3). 

The fifteen (*R*)-tryptophanol-derived oxazolopyrrolidone lactams **7h**–**u** and **7j’** obtained, as well as **7c**, were screened at 50 µM in AGS cell line (Table 2). 

(*R*)-tryptophanol-derived oxazolopyrrolidones **7h** and **7r** showed similar antiproliferative activity to **7c**, while **7j**, **7o**, and **7s** were more active than the hit compound **7c**. The presence of a pyridine (compound **7t**) or a dioxane ring (compound **7u**) led to a decrease of the antiproliferative effect in AGS cells. Additionally, *meta*-fluoro and *para*-methoxy substituents on the phenyl ring (compound **7i**) resulted in a non-significant cell death. Compounds **7n** (R^1^ = *p*-Cl-Ph), **7p** (R^1^ = *p*-OH-Ph), and **7q** (R^1^ = *p*-CH_2_OH-Ph), with bulky substituents, displayed moderate antiproliferative activity at 50 µM. The results also suggest that the presence of a *meta*-chloro substituent or electron withdrawing groups are important for the activity (**7r** and **7s** vs. **7n** and **7o**, **7r**, and **7s** vs. **7p** and **7q**). Interestingly, the two diastereomers **7j** and **7j’** had different effects in AGS cells. Diastereomer **7j**, with (3*R*, 7*R*, 7a*S*) configuration, had a high antiproliferative effect, while diastereomer **7j’** (3*R*, 7*R*, 7a*R*) had almost no effect, suggesting that the *C*-7a stereochemistry is also decisive for the antiproliferative activity of tryptophanol-derived oxazolopyrrolidone lactams in AGS cells. 

The substitution of the *N*-indole hydrogen (compound **7c**) by ethyl (compound **7k**), acetyl (compound **7l**) or *tert*-butyloxycarbonyl (compound **7m**) groups led to a decrease of activity, probably due to steric effects or because the establishment of a hydrogen bond might be important for the antiproliferative effect.

The IC_50_ values of the most promising derivatives (**7j**, **7o**, and **7s**), as well as of the hit compound **7c**, were determined in AGS cell line (Table 3). Trifluoromethyl derivative **7o** (R^1^ = *p*-CF_3_-Ph) and di-halogenated derivative **7s** (R^1^ = 3,4-Cl-Ph) were the most active derivatives with 2.3 times more potency than the hit **7c**, respectively. We then tested compounds **7o** and **7s** in four cancer cell lines of other tumor types (Table 3): MDA-MB-231 (breast adenocarcinoma), A-549 (lung carcinoma), DU-145 (prostate cancer), and MG-63 (osteosarcoma). Both compounds were much less potent in lung carcinoma cells (IC_50_ higher than 60 µM) but presented moderate activity in prostate cancer cell line DU-145 (Table 3). In osteosarcoma and breast cells, compound **7o** was around two times more active than compound **7s**. Compounds **7o** and **7s** were then evaluated in HEK 293T normal cell line [22] and, except for A-549 cells, showed selectivity towards all cancer cell lines over the non-cancer derived cell line (Table 3). 

**Figure 4:**
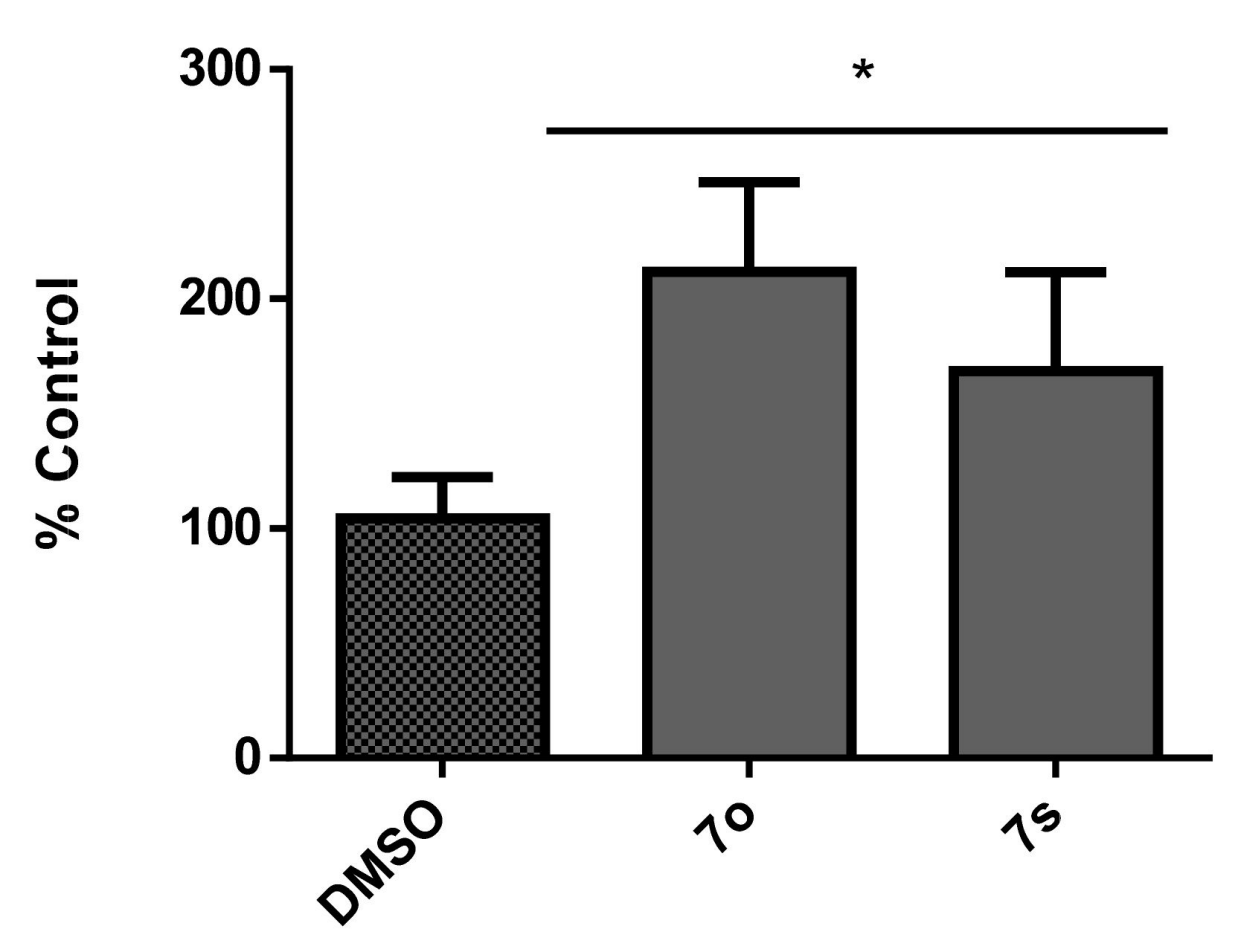
Percentage of the normalized caspase 3/7 activity in AGS cells after 48 h of exposure to the selected compounds at 12.5 µM. * Significantly different from control for *p* < 0.05.

The ability of compounds **7o** and **7s** to induce apoptosis was also explored by measuring caspase 3/7 activity in AGS cells. The assays showed that, after 48 h of compounds’ incubation at 12.5 µM, there was a significant increase of caspase 3/7 activity, indicating that the antiproliferative activity is associated with apoptosis induction (Figure 4).

### 2.3. Stability Studies in PBS, Human Plasma, and Human Liver Microsomes and Identification of Metabolites

**Figure 5:**
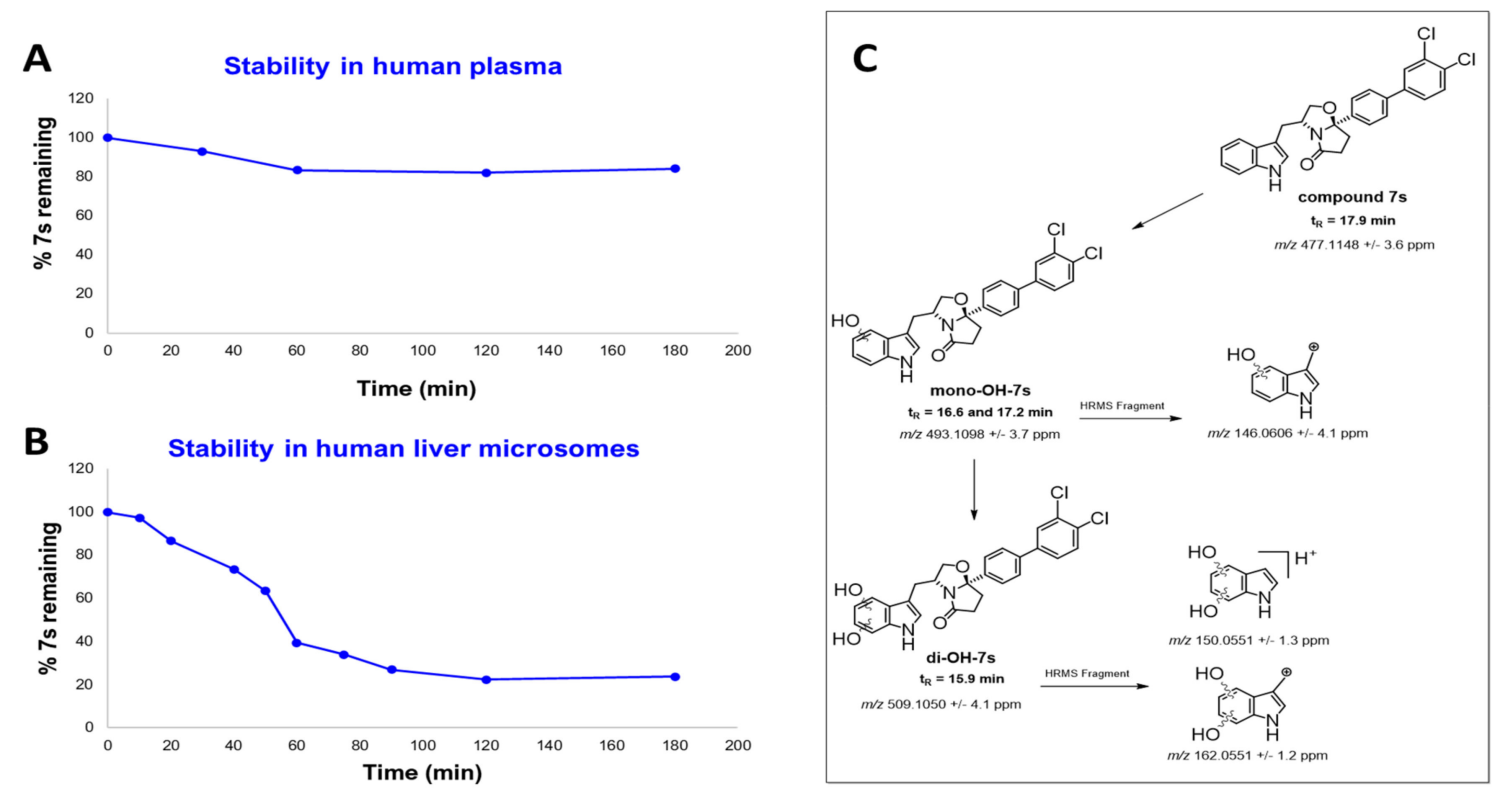
(**A**) Stability of compound **7s** in human plasma; (**B**) stability of compound **7s** in human liver microsomes; (**C**) HRMS evidence for the identification of the **mono-OH-7s** (major) and **di-OH-7s** (minor) metabolites.

Preliminary stability studies can provide useful information about possible liabilities of new drug candidates. Understanding possible clearance mechanisms and how to modulate the metabolism to reduce metabolic liability of a new bioactive chemical entity is a fundamental step in drug development that allows access to a hit compound with desirable ADME attributes [23]. The in vitro phosphate saline buffer (PBS), plasma, and metabolic stabilities for compound **7s** were evaluated. This compound showed chemical stability in PBS conditions and under plasmatic enzyme activity after 24 h of incubation, at 37 °C (Figure 5A). The in vitro metabolic stability of compound **7s** was determined upon incubation in human liver microsomes, in the presence of the Phase I cofactor NADPH (Figure 5B). This compound demonstrated to be moderately stable [24,25], with a half-life (*t*_1/2_) of 45 min (see Supplementary Materials Figure S1) and an intrinsic hepatic clearance (CL_int_) of 22.8 min^−1^·mL^−1^·Kg^−1^. Three main Phase I metabolites, stemming from mono- and di-hydroxylation of the indole moiety, were identified by LC-HRMS/MS (liquid chromatography high resolution tandem mass spectrometry) analysis. The protonated molecule of the parent compound, **7s**, is observed in the HRMS-ESI(+) full scan spectrum at *m*/*z* 477.1148 ± 3.6 ppm, with the characteristic dichlorine isotope cluster, and the base peak of the MS/MS spectrum is observed at *m*/*z* 304.0289 ± 0.3 ppm, which corresponds to the loss of the dichloro-biphenyl-dihydropyrrolone moiety from the protonated molecule (see Supplementary Materials Figure S2). A mass increment of 15.9944 *u* is observed for the protonated molecules of the two close eluting (major) metabolites at *m*/*z* 493.1100 ± 4.0 and *m*/*z* 493.1098 ± 3.7 ppm, which are, therefore, compatible with two isomer mono-hydroxylated metabolites of compound **7s**, indicated with abbreviation **mono-OH-7s** (Figure 5C, see Supplementary Materials Figure S3). The structural similarity of these two Phase I metabolites was further confirmed by the similar fragmentation patterns observed in the tandem mass spectra (see Supplementary Materials Figure S3B,C), whose base peaks correspond to the loss the dichloro-biphenyl-dihydropyrrolone moiety, similarly to what is observed for **7s**. Whereas the exact location of the hydroxyl group could not be determined, the hydroxylation of the indole moiety is suggested by the observation of the diagnostic fragment ion at *m*/*z* 146.0606 ± 4.1 ppm in the MS/MS spectra of the two mono-hydroxylated metabolites (Figure 5C, see Supplementary Materials Figure S3B,C). With retention time of 15.9 min, a minor di-hydroxilated metabolite was also identified based on the observation of the monoisotopic signal at *m*/*z* 509.1050 ± 4.1 ppm, in the full scan HRMS-ESI(+) spectra (see Supplementary Materials Figure S4B). Identification of the diagnostic fragment ion at *m*/*z* 150.0551 ± 1.3 ppm in the MS/MS spectrum confirms the di-hydroxylation on the indole ring (Figure 5C, see Supplementary Materials Figure S4B). The observation of the fragment ion at *m*/*z* 162.0551 ± 1.2 ppm (the di-hydroxylated version of the mentioned diagnostic fragment ion for **mono-OH-7s** metabolites), represents an additional evidence that the main site of Phase I biotransformation is the indole ring. This constitutes an expected metabolic transformation [26], which is not linked with drug bioactivation processes [27], and, therefore, is not anticipated to be a toxicity red flag alert. Nonetheless, taking into consideration the moderate metabolic stability of the parent compound, it might be relevant to assess the activity of hydroxylated metabolites, following further improvement of this scaffold.

## 3. Materials and Methods

### 3.1. Chemistry 

General information: THF was dried using sodium wire and benzophenone as indicator. (*R*)-Tryptophanol was obtained by reduction of (*R*)-tryptophan using lithium aluminum hydride [28]. Other reagents were obtained from commercial suppliers (Sigma-Aldrich, Alfa Aesar, and Fluorochem). General information concerning the equipment used for the elucidation of the products’ chemical structures and product characterization (NMR, melting point, optical rotations, MS, and elemental analysis) are presented in our earlier publication [21]. Multiplicities in ^1^H NMR spectra are given as: s (singlet), d (doublet), dd (double doublet), ddd (doublet of doublets of doublets), t (triplet), and m (multiplet). Compounds **7h**, **7j**, and **7j’** showed purity ≥ 95% by LC-MS, performed in a LaChrom HPLC constituted of a Merck Hitachi pump L-7100, Merck Hitachi autosampler L-7250, and a Merck Hitachi UV detector L-7400. Analyses were performed with a LiChrospher^®^100 RP-8 (5 µm) LiChroCART^®^ 250-4 column at room temperature, using a mobile phase solution constituted of 65% acetonitrile and 35% Milli-Q water. Peaks were detected at λ = 254 nm.

General procedure for the synthesis of compounds **7a**–**j**, **7j’**, and **8a**–**g**: To a suspension of enantiopure tryptophanol (0.53 mmol, 1.0 eq.) in toluene (5 mL) was added the appropriate oxocarboxylic acid (0.58 mmol, 1.1 eq.). The mixture was heated at reflux for 10–25 h in a Dean–Stark apparatus. The reaction mixture was concentrated in vacuo and the residue obtained was dissolved in EtOAc (10 mL). The organic phase was washed with saturated aqueous solution of NaHCO_3_ (15 mL) and brine (15 mL), dried over Na_2_SO_4_, filtered, and concentrated in vacuo. The residue was purified by silica gel flash chromatography using a mixture of EtOAc/*n*-hexane as eluent. 

(3*R*,7a*R*)-3-((1*H*-indol-3-yl)methyl)-7a-phenyltetrahydropyrrolo[2,1-*b*]oxazol-5(6*H*)-one (**7a**): Following the general procedure, to a solution of (*R*)-tryptophanol (0.102 g, 0.536 mmol) in toluene (5 mL) was added 3-benzoyl propionic acid (0.105 g, 0.590 mmol). Reaction time: 19 h. The compound was purified by flash chromatography (EtOAc/*n*-hexane 1:1) and recrystallized from EtOAc/*n*-hexane to give pale pink crystalline solid (0.166 g, 95%); $\alpha_{D}^{25}$ = −54.7° (*c* = 2.0, MeOH); ^1^H NMR spectra was found to be identical to the one reported [15] and obtained for compound 8a. Anal. Calcd. for C_21_H_20_N_2_O_2_·0.05H_2_O: C, 75.67%; H, 6.09%; N, 8.41%. Found C: 75.22%; H: 5.87%; N: 8.23%. The m.p. value was found to be identical to the one reported for compound **8a**.

(3*S*,7a*S*)-3-((1*H*-indol-3-yl)methyl)-7a-phenyltetrahydropyrrolo[2,1-*b*]oxazol-5(6*H*)-one (**8a**): Following the general procedure, to a solution of (*S*)-tryptophanol (0.101 g, 0.529 mmol) in toluene (5 mL) was added 3-benzoyl propionic acid (0.104 g, 0.582 mmol). Reaction time: 24 h. The compound was purified by flash chromatography (EtOAc/*n*-hexane 1:1) and recrystallized from EtOAc/*n*-hexane to give pale pink crystalline solid (0.127 g, 72%); $\alpha_{D}^{25\;}$= +40.4° (*c* = 2.0, MeOH); m.p.: 153–156 °C; ^1^H NMR (300 MHz, CDCl_3_) δ 7.99 (s, 1H, NH), 7.50 (d, *J* = 6.0 Hz, 2H, ArH), 7.46–7.29 (m, 5H, ArH), 7.17 (t, *J* = 7.5 Hz, 1H, ArH), 7.10–7.05 (m, 2H, ArH), 4.62–4.52 (m, 1H, H-3), 4.16 (t, *J* = 8.1 Hz, 1H, H-2), 3.63–3.58 (m, 1H, H-2), 3.07 (dd, *J* = 14.3, 6.2 Hz, 1H, indole-CH_2_), 2.96–2.75 (m, 1H, CH_2_), 2.68–2.35 (m, 3H, CH_2_, and indole-CH_2_), 2.34–2.14 (m, 1H, CH_2_) ppm; Anal. Calcd. for C_21_H_20_N_2_O_2_: C: 75.88%; H: 6.06%; N: 8.43%. Found C: 75.95%; H: 5.76%; N: 8.36%. ^1^H NMR spectra was found to be identical to the one reported [15].

(3*R*,7a*R*)-3-((1*H*-indol-3-yl)methyl)-7a-(4-fluorophenyl)tetrahydropyrrolo[2,1-*b*]oxazol-5(6*H*)-one (**7b**): Following the general procedure, to a solution of (*R*)-tryptophanol (0.100 g, 0.526 mmol) in toluene (5 mL) was added 3-(4-fluorobenzoyl) propionic acid (0.114 g, 0.581 mmol). Reaction time: 19 h. The compound was purified by flash chromatography (EtOAc/*n*-hexane 1:1) and recrystallized from EtOAc/*n*-hexane to give pale yellow crystalline solid (0.113 g, 70%); $\alpha_{D}^{25\;}$ = −48.8° (*c* = 2.0, MeOH); ^1^H NMR was found to be identical to the one obtained for compound 8b. Anal. Calcd. for C_21_H_19_FN_2_O_2_: C: 71.98%; H: 5.47%; N: 8.00%. Found C: 72.09%; H: 5.49%; N: 7.94%. The m.p. value was found to be identical to the one reported for compound **8b**.

(3*S*,7a*S*)-3-((1*H*-indol-3-yl)methyl)-7a-(4-fluorophenyl)tetrahydropyrrolo[2,1-*b*]oxazol-5(6*H*)-one (**8b**): Following the general procedure, to a solution of (*S*)-tryptophanol (0.102 g, 0.535 mmol) in toluene (5 mL) was added 3-(4-fluorobenzoyl) propionic acid (0.115 g, 0.588 mmol). Reaction time: 21 h. The compound was purified by flash chromatography (EtOAc/*n*-hexane 1:1) and recrystallized from EtOAc/*n*-hexane to give orange crystalline solid (0.156 g, 83%); $\alpha_{D}^{25\;}$ = +39.5° (*c* = 2.0, MeOH); m.p.: 197-198 °C; ^1^H NMR (300 MHz, CDCl_3_) δ 8.00 (s, 1H, NH), 7.51-7.41 (m, 3H, ArH), 7.33 (d, *J* = 8.1 Hz, 1H, ArH), 7.21–7.15 (m, 1H, ArH), 7.11–7.03 (m, 4H, ArH), 4.62–4.53 (m, 1H, H-3), 4.17 (dd, *J* = 8.8 Hz, 7.4 Hz, 1H, H-2), 3.59 (dd, *J* = 8.8 Hz, 6.9Hz, 1H, H-2), 3.05 (dd, *J* = 14.7 Hz, 6.2Hz, 1H, indole-CH_2_), 2.90–2.78 (m, 1H, CH_2_), 2.65–2.43 (m, 3H, CH_2_, and indole-CH_2_), 2.24 − 2.15 (m, 1H, CH_2_) ppm; ^13^C NMR (75 MHz, CDCl_3_) δ 180.3 (C=O), 162.8 (d, Cq, *J*_C-F_ = 245.3 Hz), 138.8 (d, Cq, *J* = 3.1 Hz), 136.3 (Cq), 127.5 (Cq), 126.9 (d, ArCH, *J* = 8.1 Hz), 122.3 (ArCH), 122.2 (ArCH), 119.6 (ArCH), 118.9 (ArCH), 115.5 (d, ArCH, *J* = 21.5 Hz), 111.6 (Cq), 111.3 (ArCH), 102.2 (C-7a), 72.8 (C-2), 55.8 (C-3), 35.2 (CH_2_), 32.7 (CH_2_), 29.8 (indole-CH_2_). Anal. Calcd. for C_21_H_19_FN_2_O_2_: C: 71.98%; H: 5.47%; N: 8.00%. Found C: 72.48%; H: 5.37%; N: 8.03%.

(3*R*,7a*R*)-3-((1*H*-indol-3-yl)methyl)-7a-(4-chlorophenyl)tetrahydropyrrolo[2,1-*b*]oxazol-5(6*H*)-one (**7c**): Following the general procedure, to a solution of (*R*)-tryptophanol (0.103 g, 0.541 mmol) in toluene (5 mL) was added 3-(4-chlorobenzoyl) propionic acid (0.127 g, 0.596 mmol). Reaction time: 18 h. The compound was purified by flash chromatography (EtOAc/*n*-hexane 1:1) and recrystallized from EtOAc/*n*-hexane to give pale yellow crystalline solid (0.133 g, 67%); $\alpha_{D}^{25\;}$= −63.1° (*c* = 2.0, MeOH); ^1^H NMR was found to be identical to the one obtained for compound 8c. Anal. Calcd. for C_21_H_19_ClN_2_O_2_: C: 68.76%; H: 5.18%; N: 7.62%. Found C: 68.76%; H: 5.22%; N: 7.64%. The m.p. value was found to be identical to the one reported for compound **8c**.

(3*S*,7a*S*)-3-((1*H*-indol-3-yl)methyl)-7a-(4-chlorophenyl)tetrahydropyrrolo[2,1-*b*]oxazol-5(6*H*)-one (**8c**): Following the general procedure, to a solution of (*S*)-tryptophanol (0.104 g, 0.545 mmol) in toluene (5 mL) was added 3-(4-chlorobenzoyl) propionic acid (0.128 g, 0.600 mmol). Reaction time: 23 h. The compound was purified by flash chromatography (EtOAc/*n*-hexane 1/1) and recrystallized from EtOAc/*n*-hexane to give pale pink crystalline solid (0.164 g, 82%); $\alpha_{D}^{25\;}$= +54.5° (*c* = 2.0, MeOH); m.p.: 206–208 °C; ^1^H NMR (300 MHz, CDCl_3_) δ 7.98 (s, 1H, NH), 7.47–7.32 (m, 6H, ArH), 7.21–7.16 (m, 1H, ArH), 7.12–7.06 (m, 2H, ArH), 4.62–4.52 (m, 1H, H-3), 4.17 (dd, *J* = 8.8 Hz, 7.5 Hz, 1H, H-2), 3.59 (dd, *J* = 8.8 Hz, 6.9 Hz, 1H, H-2), 3.05 (dd, *J* = 15.1 Hz, 7.5 Hz, 1H, indole-CH_2_), 2.89–2.78 (m, 1H, CH_2_), 2.65–2.44 (m, 3H, CH_2_, and indole-CH_2_), 2.22–2.14 (m, 1H, CH_2_) ppm; ^13^C NMR (75 MHz, CDCl_3_) δ 180.3 (C=O), 141.5 (Cq), 136.3 (Cq), 134.3 (Cq), 129.0 (ArCH), 127.4 (Cq), 126.7 (ArCH), 122.3 (ArCH), 122.2 (ArCH), 119.5 (ArCH), 118.8 (ArCH), 111.4 (Cq), 111.3 (ArCH), 102.1 (C-7a), 72.9 (C-2), 55.8 (C-3), 35.1 (CH2), 32.6 (CH2), 29.8 (indole-CH2). Anal. Calcd. for C_21_H_19_ClN_2_O_2_: C: 68.76%; H: 5.22%; N: 7.62%. Found C: 68.94%; H: 5.06%; N: 7.60%.

(3*R*,7a*R*)-3-((1*H*-indol-3-yl)methyl)-7a-(4-bromophenyl)tetrahydropyrrolo[2,1-*b*]oxazol-5(6*H*)-one (**7d**): Following the general procedure, to a solution of (*R*)-tryptophanol (0.102 g, 0.536 mmol) in toluene (5 mL) was added 3-(4-bromobenzoyl) propionic acid (0.153 g, 0.590 mmol). Reaction time: 18 h. The compound was purified by flash chromatography (EtOAc/*n*-hexane 1:1) and recrystallized from EtOAc/*n*-hexane to give pale pink crystalline solid (0.182 g, 83%); $\alpha_{D}^{25\;}$ = −53.6° (*c* = 2.0, MeOH); ^1^H NMR was found to be identical to the one obtained for compound 8d. Anal. Calcd. for C_21_H_19_BrN_2_O_2_·0.15H_2_O: C: 60.92%; H: 4.71%; N: 6.77%. Found C: 60.47%; H: 4.55%; N: 6.55%. The m.p. value was found to be identical to the one reported for compound **8d**.

(3*S*,7a*S*)-3-((1*H*-indol-3-yl)methyl)-7a-(4-bromophenyl)tetrahydropyrrolo[2,1-*b*]oxazol-5(6*H*)-one (**8d**): Following the general procedure, to a solution of (*S*)-tryptophanol (0.102 g, 0.536 mmol) in toluene (5 mL) was added 3-(4-bromobenzoyl) propionic acid (0.151 g, 0.589 mmol). Reaction time: 18 h. The compound was purified by flash chromatography (EtOAc/*n*-hexane 1:1) and recrystallized from EtOAc/*n*-hexane to give pale yellow crystalline solid (0.159 g, 72%); $\alpha_{D}^{25\;}$ = +52.3° (*c* = 2.0, MeOH); m.p.: 207-210 °C. ^1^H NMR (300 MHz, CDCl_3_) δ 7.97 (s, 1H, NH), 7.52–7.45 (m, 3H, ArH), 7.37–7.32 (m, 3H, ArH), 7.21–7.05 (m, 3H, ArH), 4.62–4.52 (m, 1H, H-3), 4.17 (dd, *J* = 8.8 Hz, 7.4 Hz, 1H, H-2), 3.59 (dd, *J* = 8.8 Hz, 6.9 Hz, 1H, H-2), 3.05 (dd, *J* = 14.7 Hz, 6.1 Hz, 1H, indole-CH_2_), 2.89–2.78 (m, 1H, CH2), 2.65–2.44 (m, 3H, CH_2_, and indole-CH_2_), 2.22–2.14 (m, 1H, CH_2_) ppm; ^13^C NMR (75 MHz, CDCl_3_) δ 180.3 (C=O), 142.1 (Cq), 136.3 (Cq), 131.8 (ArCH), 127.5 (Cq), 127.1 (ArCH), 122.5 (Cq), 122.2 (ArCH), 122.1 (ArCH), 119.7 (ArCH), 118.9 (ArCH), 111.6 (Cq), 111.3 (ArCH), 102.1 (C-7a), 72.9 (C-2), 55.8 (C-3), 35.1 (CH2), 32.7 (CH_2_), 29.8 (indole-CH_2_). Anal. Calcd. for C_21_H_19_BrN_2_O_2_: C: 61.33%; H: 4.66%; N: 6.81%. Found C: 61.26%; H: 4.48%; N: 6.76%.

(3*R*,7a*R*)-3-((1*H*-indol-3-yl)methyl)-7a-(*p*-tolyl)tetrahydropyrrolo[2,1-*b*]oxazol-5(6*H*)-one (**7e**): Following the general procedure, to a solution of (*R*)-tryptophanol (0.103 g, 0.541 mmol) in toluene (5 mL) was added 3-(4-methylbenzoyl) propionic acid (0.114 g, 0.596 mmol). Reaction time: 19 h. The compound was purified by flash chromatography (EtOAc/*n*-hexane 1:1) and recrystallized from EtOAc/*n*-hexane to give pale pink crystalline solid (0.160 g, 86%); $\alpha_{D}^{25\;}$= −58.7° (*c* = 2.0, MeOH); ^1^H NMR was found to be identical to the one obtained for compound 8e. Anal. Calcd. for C_22_H_22_N_2_O_2_: C: 76.28%; H: 6.40%; N: 8.09%. Found C: 75.87%; H: 6.23%; N: 8.06%. The m.p. value was found to be identical to the one reported for compound **8e**.

(3*S*,7a*S*)-3-((1*H*-indol-3-yl)methyl)-7a-(*p*-tolyl)tetrahydropyrrolo[2,1-*b*]oxazol-5(6*H*)-one (**8e**): Following the general procedure, to a solution of (*S*)-tryptophanol (0.100 g, 0.526 mmol) in toluene (5 mL) was added 3-(4-methylbenzoyl) propionic acid (0.112 g, 0.583 mmol). Reaction time: 18 h. The compound was purified by flash chromatography (EtOAc/*n*-hexane 1:1) and recrystallized from EtOAc/*n*-hexane to give pale pink crystalline solid (0.097 g, 53%); $\alpha_{D}^{25\;}$= +45.1° (*c* = 2.0, MeOH); m.p.: 210–213 °C. ^1^H NMR (300 MHz, CDCl_3_) δ 8.00 (s, 1H, NH), 7.47–7.32 (m, 4H, ArH), 7.21–7.06 (m, 5H, ArH), 4.60–4.50 (m, 1H, H-3), 4.15 (dd, *J* = 8.7 Hz, 7.4 Hz, 1H, H-2), 3.61 (dd, *J* = 8.8 Hz, 6.9 Hz, 1H, H-2), 3.09 (dd, *J* = 14.7 Hz, 6.1 Hz, 1H, indole-CH_2_), 2.90–2.79 (m, 1H, CH_2_), 2.64-2.43 (m, 3H, CH_2_, and indole-CH_2_), 2.39 (s, 3H, CH_3_), 2.26-2.17 (m, 1H, CH_2_) ppm; ^13^C NMR (75 MHz, CDCl_3_) δ 180.3 (C=O), 139.9 (Cq), 138.2 (Cq), 136.3 (Cq), 129.3 (ArCH), 127.5 (Cq), 125.2 (ArCH), 122.3 (ArCH), 122.2 (ArCH), 119.6 (ArCH), 119.0 (ArCH), 111.9 (Cq), 111.2 (ArCH), 102.6 (C-7a), 72.9 (C-2), 55.7 (C-3), 35.4 (CH_2_), 32.9 (CH_2_), 29.9 (indole-CH_2_), 21.4 (CH_3_). Anal. Calcd. for C_22_H_22_N_2_O_2_: C: 76.28%; H: 6.40%; N: 8.09%. Found C: 76.49%; H: 6.27%; N: 8.16%.

(3*R*,7a*R*)-3-((1*H*-indol-3-yl)methyl)-7a-(4-methoxyphenyl)tetrahydropyrrolo[2,1-*b*]oxazol-5(6*H*)-one (**7f**): Following the general procedure, to a solution of (*R*)-tryptophanol (0.100 g, 0.526 mmol) in toluene (5 mL) was added 3-(4-methoxybenzoyl) propionic acid (0.121 g, 0.581 mmol). Reaction time: 24 h. The compound was purified by flash chromatography (EtOAc/*n*-hexane 1:1) and recrystallized from EtOAc/*n*-hexane to give pale yellow crystalline solid (0.106 g, 56%); $\alpha_{D}^{25\;}$= −43.0° (*c* = 1.0, MeOH); ^1^H NMR was found to be identical to the one obtained for compound 8f. Anal. Calcd. for C_22_H_22_N_2_O_3_·0.15H_2_O: C: 72.36%; H: 6.17%; N: 7.67%. Found C: 72.22%; H: 6.21%; N: 7.53%. The m.p. value was found to be identical to the one reported for compound **8f**.

(3*S*,7a*S*)-3-((1*H*-indol-3-yl)methyl)-7a-(4-methoxyphenyl)tetrahydropyrrolo[2,1-*b*]oxazol-5(6*H*)-one (**8f**): Following the general procedure, to a solution of (*S*)-tryptophanol (0.101 g, 0.533 mmol) in toluene (5 mL) was added 3-(4-methoxybenzoyl) propionic acid (0.122 g, 0.586 mmol). Reaction time: 25 h. The compound was purified by flash chromatography (EtOAc/*n*-hexane 1:1) and recrystallized from EtOAc/*n*-hexane to give pale yellow crystalline solid (0.134 g, 69%); $\alpha_{D}^{25\;}$ = +48.1° (*c* = 1.0, MeOH); m.p.: 185–187 °C. ^1^H NMR (300 MHz, CDCl_3_) δ 8.00 (s, 1H, NH), 7.47–7.32 (m, 4H, ArH), 7.20–7.05 (m, 3H, ArH), 6.92–6.89 (m, 2H, ArH), 4.61–4.50 (m, 1H, H-3), 4.15 (dd, *J* = 8.4 Hz, 7.7 Hz, 1H, H-2), 3.84 (s, 3H, O-CH_3_), 3.61 (dd, *J* = 8.7 Hz, 7.0 Hz, 1H, H-2), 3.08 (dd, *J* = 14.7 Hz, 6.2 Hz, 1H, indole-CH_2_), 2.90–2.79 (m, 1H, CH_2_), 2.64–2.44 (m, 3H, CH_2_, and indole-CH_2_), 2.25–2.17 (m, 1H, CH_2_) ppm; ^13^C NMR (75 MHz, CDCl3) δ 180.2 (C=O), 159.7 (Cq), 136.3 (Cq), 134.9 (Cq), 127.5 (Cq), 126.6 (ArCH), 122.2 (ArCH), 119.5 (ArCH), 119.0 (ArCH), 114.1 (ArCH), 111.8 (Cq), 111.2 (ArCH), 102.5 (C-7a), 72.8 (C-2), 55.7 (OCH_3_), 55.5 (C-3), 35.3 (CH_2_), 32.8 (CH_2_), 29.8 (indole-CH_2_). Anal. Calcd. for C_22_H_22_N_2_O_3_: C: 72.91%; H: 6.12%; N: 7.73%. Found C: 72.73%; H: 5.76%; N: 7.73%.

(3*R*,7a*R*)-3-((1*H*-indol-3-yl)methyl)-7a-(4-(methylsulfonyl)phenyl)tetrahydropyrrolo[2,1-*b*]oxazol-5(6*H*)-one (**7g**): Following the general procedure, to a solution of (*R*)-tryptophanol (0.106 g, 0.557 mmol) in toluene (5 mL) was added 3-(4-methylsulfonylbenzoyl) propionic acid (0.157 g, 0.613 mmol). Reaction time: 22 h. The compound was purified by flash chromatography (EtOAc/*n*-hexane 7:3) and recrystallized from EtOAc/*n*-hexane to give a pale yellow crystalline solid (0.171g, 75%); $\alpha_{D}^{25\;}$= −57.2° (*c* = 2.0, MeOH); ^1^H NMR was found to be identical to the one obtained for compound 8g. Anal. Calcd. for C_22_H_22_N_2_O_4_S: C: 64.37%; H: 5.40%; N: 6.82%. Found C: 64.31%; H: 5.32%; N: 6.81%. The m.p. value was found to be identical to the one reported for compound **8g**.

(3*S*,7a*S*)-3-((1*H*-indol-3-yl)methyl)-7a-(4-(methylsulfonyl)phenyl)tetrahydropyrrolo[2,1-*b*]oxazol-5(6*H*)-one (**8g**): Following the general procedure, to a solution of (*S*)-tryptophanol (0.100 g, 0.526 mmol) in toluene (5 mL) was added 3-(4-methylsulfonylbenzoyl) propionic acid (0.148 g, 0.578 mmol). Reaction time: 23 h. The compound was purified by flash chromatography (EtOAc/*n*-hexane 7:3) and recrystallized from EtOAc/*n*-hexane to give yellow crystalline solid (0.131 g, 61%); $\alpha_{D}^{25\;}$= +66.9 (*c* = 2.0, MeOH); m.p.: 205–207 °C. ^1^H NMR (300 MHz, CDCl_3_) δ 8.01 (s, 1H, NH), 7.90–7.88 (m, 2H, ArH), 7.62–7.59 (m, 2H, ArH), 7.43 (d, *J* = 7.9 Hz, 1H, ArH), 7.33 (d, *J* = 8.1 Hz, 1H, ArH), 7.21–7.16 (m, 1H, ArH), 7.11–7.06 (m, 1H, ArH), 7.00 (d, *J* = 2.3 Hz, 1H, ArH), 4.66–4.56 (m, 1H, H-3), 4.22 (dd, *J* = 8.9, 7.4 Hz, 1H, H-2), 3.61 (dd, *J* = 8.9, 7.0 Hz, 1H, H-2), 3.10 (s, 3H, SO_2_CH_3_), 3.00 (dd, *J* = 15.5, 5.9 Hz, 1H, indole-CH_2_), 2.92–2.75 (m, 1H, CH_2_), 2.70–2.45 (m, 3H, CH_2_, and indole-CH_2_), 2.21–2.13 (m, 1H, CH_2_) ppm; ^13^C NMR (75 MHz, CDCl_3_) δ 180.1 (C=O), 149.2 (Cq), 140.5 (Cq), 136.3 (Cq), 128.0 (ArCH), 127.5 (Cq), 126.2 (ArCH), 122.4 (ArCH), 119.7 (ArCH), 118.7 (ArCH), 111.4 (Cq), 111.1 (ArCH), 101.8 (C-7a), 72.9 (C-2), 56.0 (C-3), 44.6 (SO_2_CH_3_), 35.1 (CH_2_), 32.6 (CH_2_), 29.5 (indole-CH_2_). Anal. Calcd. for C_22_H_22_N_2_O_4_S: C: 64.37%; H: 5.40%; N: 6.82%. Found C: 64.59%; H: 5.51%; N: 6.69%.

(3*R*,7a*R*)-3-((1*H*-indol-3-yl)methyl)-7a-(3-chloro-4-methylphenyl)tetrahydropyrrolo[2,1-*b*]oxazol-5(6*H*)-one (**7h**): Following the general procedure, to a solution of (*R*)-tryptophanol (0.041 g, 0.218 mmol) in toluene (2 mL) was added 4-(3-chloro-4-methylphenyl)-4-oxobutanoic acid (0.058 g, 0.254 mmol). Reaction time: 10 h. The compound was purified by flash chromatography (EtOAc/*n*-hexane 1:1) and recrystallized from CH_2_Cl_2_/*n*-hexane to give a white solid (0.077 g, 93%); $\alpha_{D}^{25\;}$= −29.6° (*c* = 1.0, CH_2_Cl_2_); m.p.: 171–172 °C. ^1^H NMR (300 MHz, CDCl_3_): δ 7.97 (s, 1H, NH), 7.50 (d, *J* = 1.4 Hz, 1H, ArH), 7.48 (d, *J* = 8.1 Hz, 1H, ArH), 7.33 (d, *J* = 8.0 Hz, 1H, ArH), 7.28–7.23 (m, 2H, ArH), 7.21–7.15 (m, 1H, ArH), 7.13–7.09 (m, 1H, ArH), 7.06 (d, *J* = 2.4 Hz, 1H, ArH), 4.62–4.50 (m, 1H, H-3), 4.16 (dd, *J* = 8.8, 7.4 Hz, 1H, H-2), 3.61 (dd, *J* = 8.8, 6.9 Hz, 1H, H-2), 3.10 (dd, *J* = 14.6, 6.0 Hz, 1H, indole-CH_2_), 2.85 (ddd, *J* = 16.3, 9.8, 8.1 Hz, 1H, CH_2_), 2.62 (dd, *J* = 10.1, 3.3 Hz, 1H, CH_2_), 2.50 (ddd, *J* = 18.4, 9.6, 6.1 Hz, 2H, CH_2_, and indole-CH_2_), 2.41 (s, 3H, CH_3_), 2.25–2.15 (m, 1H, CH_2_); ^13^C NMR (75 MHz, CDCl_3_) δ 180.1 (C=O), 160.6 (Cq), 142.3 (Cq), 136.2 (Cq), 134.9 (Cq), 131.4 (ArCH), 127.4 (Cq), 125.9 (ArCH), 123.5 (ArCH), 122.3 (ArCH), 122.2 (ArCH), 119.6 (Cq), 118.9 (ArCH), 111.6 (ArCH), 111.2 (ArCH), 101.8 (C-7a), 72.9 (C-2), 55.7 (C-3), 35.1 (CH_2_), 32.7 (CH_2_), 29.8 (indole-CH_2_), 20.0 (CH_3_). LRMS (ESI) *m*/*z* calcd for C_22_H_21_ClN_2_O_2_: 380, found 381 [M+H]^+^.

(3*R*,7a*R*)-3-((1*H*-indol-3-yl)methyl)-7a-(3-fluoro-4-methoxyphenyl)tetrahydropyrrolo[2,1-*b*]oxazol-5(6*H*)-one (**7i**): Following the general procedure, to a solution of (*R*)-tryptophanol (0.100 g, 0.526 mmol) in toluene (5 mL) was added 3-(3-fluoro-4-methoxybenzoyl) propionic acid (0.131 g, 0.578 mmol). Reaction time: 22 h. The compound was purified by flash chromatography (EtOAc/*n*-hexane 1:1) and recrystallized from EtOAc/*n*-hexane to give a pale yellow crystalline solid (0.139 g, 69%); $\alpha_{D}^{25\;}$ = −51.3° (*c* = 2.0, MeOH); m.p.: 131–132 °C; ^1^H NMR (300 MHz, (CDCl_3_) δ 8.01 (s, 1H, NH), 7.47 (d, *J* = 7.9 Hz, 1H, ArH), 7.33 (d, *J* = 8.0 Hz, 1H, ArH), 7.23–7.04 (m, 5H, ArH), 6.93 (t, *J* = 8.4 Hz, 1H, ArH), 4.61–4.41 (m, 1H, H-3), 4.16 (dd, *J* = 8.7, 7.4 Hz, 1H, H-2), 3.92 (s, 3H, O-CH_3_), 3.61 (dd, *J* = 8.8, 6.9 Hz, 1H, H-2), 3.08 (dd, *J* = 13.9, 6.0 Hz, 1H, indole-CH_2_), 2.90–2.83 (m, 1H, CH_2_), 2.64–2.43 (m, 3H, CH_2, and indole_-CH_2_), 2.24–2.15 (m, 1H, CH_2_) ppm; ^13^C NMR (75 MHz, CDCl_3_) δ 180.2 (C=O), 152.2 (d, Cq, *J*_C-F_ = 245.2 Hz), 147.5 (Cq), 147.3 (Cq), 136.3 (Cq), 135.7 (d, Cq, *J* = 5.2 Hz), 127.4 (Cq), 122.0 (d, ArCH, *J* = 6.3 Hz), 120.8 (d, ArCH, *J* = 3.5 Hz), 119.5 (ArCH), 118.8 (ArCH), 113.2 (d, ArCH, *J* = 1.6 Hz), 113.2 (ArCH), 111.5 (Cq), 111.3 (ArCH), 101.9 (C-7a), 72.9 (C-2), 56.5 (C-3), 55.8 (OCH_3_), 35.2 (CH_2_), 32.9 (CH_2_), 29.8 (indole-CH_2_). Anal. Calcd. for C_22_H_21_FN_2_O_3_: C: 69.46%; H: 5.56%; N: 7.36%. Found C: 69.49%; H: 5.76%; N: 7.12%.

(3*R*,7a*R*,7*S*)-3-((1*H*-indol-3-yl)methyl)-7a-(4-chlorophenyl)-7-methyltetrahydropyrrolo[2,1-*b*]oxazol-5(6*H*)-one (**7j**) and (3*R*,7a*R*,7*R*)-3-((1*H*-indol-3-yl)methyl)-7a-(4-chlorophenyl)-7-methyltetrahydropyrrolo[2,1-*b*]oxazol-5(6*H*)-one (**7j‘**): Following the general procedure, to a solution of (*R*)-tryptophanol (0.039 g, 0.207 mmol) in toluene (2 mL) was added 4-(4-chlorophenyl)-3-methyl-4-oxobutanoic acid (0.057 g, 0.239 mmol). Reaction time: 17 h. Two compounds were purified by flash chromatography (EtOAc/*n*-hexane 2:3) and recrystallized from CH_2_Cl_2_/*n*-hexane.

(**7j**): The product was obtained as a white solid (0.055 g, 69%); $\alpha_{D}^{25\;}$= −30.5° (*c* = 1.0, CH_2_Cl_2_); m.p.: 201–202 °C. ^1^H NMR (300 MHz, CDCl_3_): δ 8.00 (s, 1H, NH), 7.41 (d, *J* = 8.6 Hz, 3H, ArH), 7.34 (d, *J* = 8.8 Hz, 3H, ArH), 7.18 (t, *J* = 7.4 Hz, 1H, ArH), 7.11 (s, 1H, ArH), 7.07 (d, *J* = 7.4 Hz, 1H, ArH), 4.67–4.56 (m, 1H, H-3), 4.13 (t, *J* = 8.0 Hz, 1H, H-2), 3.58 (dd, *J* = 8.5, 6.6 Hz, 1H, H-2), 3.04–2.85 (m, 2H, CH_2_, and indole-CH_2_), 2.44 (td, *J* = 15.1, 8.1 Hz, 2H, CH_2_, and indole-CH_2_), 2.18 (dd, *J* = 17.3, 5.6 Hz, 1H, CH_2_), 1.12 (d, *J* = 7.1 Hz, 3H, CH_3_); ^13^C NMR (75 MHz, CDCl_3_) δ 181.1 (C=O), 141.4 (Cq), 136.2 (Cq), 134.2 (Cq), 128.9 (ArCH), 127.5 (Cq), 127.0 (ArCH), 122.3 (ArCH), 122.1 (ArCH), 119.6 (ArCH), 118.8 (ArCH), 111.8 (Cq), 111.2 (ArCH), 103.0 (C-7a), 72.3 (C-2), 56.5 (C-3), 40.0 (CH_2_), 39.7 (CH_2_), 29.7 (indole-CH_2_), 14.0 (CH_3_). LRMS (ESI) *m*/*z* calcd for C_22_H_21_ClN_2_O_2_: 380, found 381 [M+H]^+^.

(**7j’**): The product was obtained as white solid (0.014 g, 18%); $\alpha_{D}^{25\;}$ = −45.7° (*c* = 1.0, CH_2_Cl_2_); m.p.: 205-206 °C. ^1^H NMR (300 MHz, CDCl_3_): δ 7.95 (s, 1H, NH), 7.48 (d, *J* = 7.9 Hz, 1H, ArH), 7.34 (t, *J* = 8.7 Hz, 5H, ArH), 7.19 (t, *J* = 7.3 Hz, 1H, ArH), 7.10 (t, *J* = 7.3 Hz, 1H, ArH), 6.99 (s, 1H, ArH), 4.55 (td, *J* = 12.4, 6.8 Hz, 1H, H-3), 4.24–4.17 (m, 1H, H-2), 3.64 (dd, *J* = 8.6, 7.1 Hz, 1H, H-2), 3.10 (dd, *J* = 14.7, 5.3 Hz, 1H, indole-CH_2_), 2.78–2.63 (m, 2H, CH_2_), 2.48 (dt, *J* = 15.8, 8.1 Hz, 2H, CH_2_, and indole-CH_2_), 0.65 (d, *J* = 6.5 Hz, 3H, CH_3_); ^13^C NMR (75 MHz, CDCl_3_) δ 177.1 (C=O), 137.9 (Cq), 136.3 (Cq), 134.4 (Cq), 128.7 (ArCH), 127.9 (ArCH), 127.5 (Cq), 122.4 (ArCH), 122.2 (ArCH), 119.7 (ArCH), 119.0 (ArCH), 111.6 (Cq), 111.2 (ArCH), 104.1 (C-7a), 73.5 (C-2), 54.8 (C-3), 42.1 (CH_2_), 41.3 (CH_2_), 29.6 (indole-CH_2_), 16.4 (CH_3_). LRMS (ESI) *m*/*z* calcd for C_22_H_21_ClN_2_O_2_: 380, found 381 [M+H]^+^.

General procedure for the synthesis of **7k–l**: The (*R*)-tryptophanol-derived oxazolopyrrolidone lactam (0.129 mmol) was dissolved in dry DMF (5 mL), and the solution was cooled to 0 °C, under N_2_ atmosphere. Sodium hydride (NaH) in 60% dispersion in mineral oil (0.250 mmol, 2.0 eq.) was added portion wise and the mixture stirred for 15 min. The appropriate protecting reagent (0.320 mmol, 2.5 eq.) was added and the reaction mixture stirred at room temperature for 3–6 h. After reaction completion, water (10 mL) was added followed by EtOAc (10 mL). The aqueous phase was washed with EtOAc (2x10 mL); the combined organic phases were washed with brine (10 mL), dried with MgSO_4_, and concentrated in vacuo. The residue was purified by silica gel flash chromatography using EtOAc/*n*-hexane as eluent.

(3*R*,7a*R*)-7a-(4-chlorophenyl)-3-((1-ethyl-1*H*-indol-3-yl)methyl)tetrahydropyrrolo[2,1-*b*]oxazol-5(6*H*)-one (**7k**): Following the general procedure, to a solution of 7c (0.120 g, 0.327 mmol) in DMF (13.5 mL) was added NaH (0.016 g, 0.654 mmol) and ethyl iodide (65.4 µL, 0.818 mmol). Reaction time: 3 h. The compound was purified by flash chromatography (EtOAc/*n*-hexane 1:2) to afford the title compound as a pale yellow oil (0.101 g, 78%); ^1^H NMR (300 MHz, CDCl_3_) δ 7.39 (d, *J* = 7.9 Hz, 1H. ArH), 7.36–7.17 (m, 5H, ArH), 7.16–7.08 (m, 1H, ArH), 7.04–6.97 (m, 1H, ArH), 6.87 (s, 1H, ArH), 4.48 (m, 1H, H-3), 4.08 (dd, *J* = 8.8, 7.5 Hz, 1H, H-2), 4.01 (q, *J* = 7.3 Hz, 2H, CH_2_CH_3_), 3.52 (dd, *J* = 8.8, 7.0 Hz, 1H, H-2), 3.01 (dd, *J* = 14.6, 5.3 Hz, 1H, indole-CH_2_), 2.84–2.70 (m, 1H, CH_2_), 2.58–2.36 (m, 3H, CH_2_, and indole-CH_2_), 2.15–2.07 (m, 1H, CH_2_), 1.33 (t, *J* = 7.3 Hz, 3H, CH_2_CH_3_); ^13^C NMR (75 MHz, CDCl_3_) δ 180.2 (C=O), 141.6 (Cq), 136.1 (Cq), 134.3 (Cq), 129.0 (ArCH), 128.1 (Cq), 126.8 (ArCH), 125.3 (ArCH), 121.7 (ArCH), 119.2 (ArCH), 119.1 (ArCH), 110.0 (Cq), 109.4 (ArCH), 102.1 (C-7a), 72.9 (C-2), 55.9 (C-3), 40.9 (CH_2_CH_3_), 35.2 (CH_2_), 32.8 (CH_2_), 29.7 (indole-CH_2_), 15.6 (CH_2_CH_3_). Anal. Calcd. for C_23_H_23_ClN_2_O_2_: C: 69.96%; H: 5.87%; N: 7.09%. Found C: 70.12%; H: 6.40%; N: 6.95%.

(3*R*,7a*R*)-3-((1-acetyl-1*H*-indol-3-yl)methyl)-7a-(4-chlorophenyl)tetrahydropyrrolo[2,1-*b*]oxazol-5(6*H*)-one (**7l**): Following the general procedure, to a solution of 7c (0.094 g, 0.256 mmol) in DMF (9.5 mL) was added NaH (12.3 mg, 0.512 mmol) and acetic anhydride (60.6 µL, 0.641 mmol). Reaction time: 6 h. The compound was purified by flash chromatography (EtOAc/*n*-hexane 1:1) to afford the title compound as a white powder (0.072 g, 69%); m.p.: 66–67 °C; ^1^H NMR (300 MHz, CDCl_3_) δ 8.32 (d, *J* = 7.8 Hz, 1H, ArH), 7.56 (s, 1H, ArH), 7.28–7.11 (m, 7H, ArH), 4.61–4.45 (m, 1H, H-3), 4.20 (dd, *J* = 8.7, 7.6 Hz, 1H, H-2), 3.49 (dd, *J* = 8.7, 6.5 Hz, 1H, H-2), 2.80–2.52 (m, 3H, CH_2_, and indole-CH_2_), 2.52 (s, 3H, CH_3_), 2.48–2.35 (m, 2H, CH_2_, and indole-CH_2_), 2.16–2.02 (m, 1H, CH_2_); ^13^C NMR (75 MHz, CDCl_3_) δ 180.9 (C=O), 168.9 (C=O), 141.2 (Cq), 135.9 (Cq), 134.5 (Cq), 130.6 (Cq), 129.1 (ArCH), 126.6 (ArCH), 125.5 (ArCH), 123.7 (ArCH), 123.3 (ArCH), 118.7 (ArCH), 118.3 (Cq), 116.8 (ArCH), 102.4 (C-7a), 72.6 (C-2), 54.9 (C-3), 34.7 (CH_2_), 32.4 (CH_2_), 29.6 (indole-CH_2_), 24.2 (CH_3_). Anal. Calcd. for C_23_H_21_ClN_2_O_3_: C: 67.56%; H: 5.18%; N: 6.85%. Found C: 67.37%; H: 5.47%; N: 6.72%.

Procedure for the synthesis of *tert*-butyl 3-(((3*R*,7a*R*)-7a-(4-chlorophenyl)-5-oxohexahydropyrrolo[2,1-*b*]oxazol-3-yl)methyl)-1*H*-indole-1-carboxylate (**7m**): To a solution of 7c (0.070 g, 0.191 mmol) in THF (7.0 mL) was added anhydrous Et_3_N (58.6 µL, 0.420 mmol), DMAP (0.006 g, 0.048 mmol), and Boc_2_O (0.054 g, 0.248 mmol). The reaction mixture was stirred at room temperature for 3 h. After reaction completion, the mixture was concentrated in vacuo and the crude was dissolved in EtOAc (20 mL). The organic phase was washed with a sat. sol. of NH_4_Cl (2 × 15 mL), a sat. sol. of NaHCO_3_ (2 × 15 mL) and brine (15 mL). The combined organic phases were dried with MgSO_4_, concentrated in vacuo and the compound was purified by flash chromatography (EtOAc/*n*-hexane 2:3) to afford the title compound as a pale yellow powder (0.059 g, 66%); m.p.: 163–165 °C; ^1^H NMR (300 MHz, CDCl_3_) δ 8.01 (d, *J* = 7.6 Hz, 1H, ArH), 7.37–7.30 (m, 4H, ArH), 7.29–7.18 (m, 3H, ArH), 7.14 (td, *J* = 7.5, 1.1 Hz, 1H, ArH), 4.57–4.40 (m, 1H, H-3), 4.14 (dd, *J* = 8.8, 7.5 Hz, 1H, H-2), 3.51 (dd, *J* = 8.9, 7.0 Hz, 1H, H-2), 2.90 (ddd, *J* = 14.7, 5.7, 1.2 Hz, 1H, indole-CH_2_), 2.85–2.70 (m, 1H, CH_2_), 2.59–2.30 (m, 3H, CH_2_, and indole-CH_2_), 2.16–2.05 (m, 1H, CH_2_), 1.58 (s, 9H, C(CH_3_)_3_); ^13^C NMR (75 MHz, CDCl_3_) δ 180.3 (C=O), 149.8 (C=O), 141.4 (Cq), 134.4 (Cq), 130.4 (Cq), 129.1 (ArCH), 126.6 (ArCH), 124.7 (ArCH), 123.5 (ArCH), 122.7 (ArCH), 119.1 (ArCH), 116.1 (Cq), 115.4 (ArCH), 102.1 (C-7a), 83.8 (C(CH_3_)_3_), 72.8 (C-2), 55.2 (C-3), 35.2 (CH_2_), 32.7 (CH_2_), 29.5 (indole-CH_2_), 28.4 (C(CH_3_)_3_); Anal. Calcd. for C_26_H_27_ClN_2_O_4_: C: 66.88%; H: 5.83%; N: 6.00%. Found C: 66.90%; H: 6.16%; N: 5.89%.

General procedure for the synthesis of **7n–u**: To a solution of the appropriate tryptophanol-derived oxazolopiperidone lactams (0.230 mmol) in dioxane (2.3 mL) was added Pd(PPh_3_)_2_Cl_2_ (0.023 mmol, 0.1 eq) and 1 M aq. sol. of Na_2_CO_3_ (690 µL), followed by the appropriate boronic acid (0.280 mmol,1.2 eq.). The resulting mixture was degassed and stirred at 100 °C, under N_2_ atmosphere, for 2–5 h. After cooling to room temperature, the reaction mixture was diluted with CH_2_Cl_2_, filtered in celite, and concentrated in vacuo. The residue was purified by silica gel flash chromatography using EtOAc/*N*-hexane as eluent.

(3*R*,7a*R*)-3-((1*H*-indol-3-yl)methyl)-7a-(4’-chloro-[1,1’-biphenyl]-4-yl)tetrahydropyrrolo[2,1-*b*]oxazol-5(6*H*)-one (**7n**): Following the general procedure, to a solution of 7d (0.036 g, 0.088 mmol) in dioxane (1.0 mL) was added Pd(PPh_3_)_2_Cl_2_ (0.003 g, 8.8 µmol), 1 M aq. sol. of Na_2_CO_3_ (266 µL), and 4-chlorophenylboronic acid (0.017 g, 0.107 mmol). Reaction time: 4 h. The compound was purified by flash chromatography (EtOAc/*n*-hexane 2:3) to afford the title compound as a white solid (0.036 g, 94%); m.p.: 201–204 °C; ^1^H NMR (300 MHz, CDCl_3_) δ 8.08 (s, 1H, NH), 7.63–7.50 (m, 6H, ArH), 7.48–7.39 (m, 3H, ArH), 7.33 (d, *J* = 8.1 Hz, 1H, ArH), 7.22–7.12 (m, 1H, ArH), 7.12–7.01 (m, 2H, ArH), 4.70–4.52 (m, 1H, H-3), 4.20 (dd, *J* = 8.7, 7.4 Hz, 1H, H-2), 3.66 (dd, *J* = 8.8, 6.8 Hz, 1H, H-2), 3.11 (dd, *J* = 15.1, 6.6 Hz, 1H, indole-CH_2_), 3.01–2.78 (m, 1H, CH_2_), 2.73–2.44 (m, 3H, CH_2_, and indole-CH_2_), 2.38–2.17 (m, 1H, CH_2_); ^13^C NMR (75 MHz, CDCl_3_) δ 180.3 (C=O), 142.3 (Cq), 140.1 (Cq), 139.1 (Cq), 136.3 (Cq), 133.9 (Cq), 129.2 (ArCH), 128.5 (ArCH), 127.5 (Cq), 127.4 (ArCH), 125.9 (ArCH), 122.3 (ArCH), 122.2 (ArCH), 119.6 (ArCH), 118.9 (ArCH), 111.8 (Cq), 111.3 (ArCH), 102.4 (C-7a), 73.0 (C-2), 55.8 (C-3), 35.2 (CH_2_), 32.8 (CH_2_), 29.9 (indole-CH_2_); Anal. Calcd. for C_27_H_23_ClN_2_O_2_: C: 73.21%; H: 5.23%; N: 6.32%. Found C: 73.56%; H: 5.83%; N: 5.92%.

(3*R*,7a*R*)-3-((1*H*-indol-3-yl)methyl)-7a-(4’-(trifluoromethyl)-[1,1’-biphenyl]-4-yl)tetrahydropyrrolo[2,1-*b*]oxazol-5(6*H*)-one (**7o**): Following the general procedure, to a solution of 7d (0.050 g, 0.122 mmol) in dioxane (1.4 mL) was added Pd(PPh_3_)_2_Cl_2_ (0.004 g, 12.2 µmol), 1 M aq. sol. of Na_2_CO_3_ (370 µL), and 4-(trifluoromethyl)phenylboronic acid (0.028 g, 0.148 mmol). Reaction time: 3 h. The compound was purified by flash chromatography (EtOAc/*n*-hexane 2:3) to afford the title compound as a white solid (0.050 g, 86%); m.p.: 201–203 °C; ^1^H NMR (300 MHz, CDCl_3_) δ 7.99 (s, 1H, NH), 7.72 (s, 4H, ArH), 7.64–7.55 (m, 4H, ArH), 7.43 (d, *J* = 7.5 Hz, 1H, ArH), 7.33 (d, *J* = 8.1 Hz, 1H, ArH), 7.21–7.14 (m, 1H, ArH), 7.12–7.03 (m, 2H, ArH), 4.67–4.53 (m, 1H, H-3), 4.20 (dd, *J* = 8.8, 7.4 Hz, 1H, H-2), 3.65 (dd, *J* = 8.8, 6.8 Hz, 1H, H-2), 3.10 (dd, *J* = 15.0, 6.5 Hz, 1H, indole-CH_2_), 2.91–2.83 (m, 1H, CH_2_), 2.67–2.48 (m, 3H, CH_2_, and indole-CH_2_), 2.33–2.22 (m, 1H, CH_2_); ^13^C NMR (75 MHz, CDCl_3_) δ 180.3 (C=O), 143.9 (Cq), 142.7 (Cq), 139.8 (Cq), 136.0 (Cq), 129.5 (q, Cq, J = 32.3 Hz), 127.8 (ArCH), 127.6 (ArCH), 127.1 (Cq), 125.8 (q, ArCH, J = 3.7 Hz), 125.7 (ArCH), 124.2 (q, Cq, J_C-F_ = 270.2 Hz), 122.3 (ArCH), 122.2 (ArCH), 119.6 (ArCH), 118.1 (ArCH), 111.7 (Cq), 111.2 (ArCH), 102.4 (C-7a), 72.9 (C-2), 55.8 (C-3), 35.2 (CH_2_), 32.8 (CH_2_), 29.9 (indole-CH_2_); Anal. Calcd. for C_28_H_23_F_3_N_2_O_2_: C: 70.58%; H: 4.87%; N: 5.88%. Found C: 70.09%; H: 5.19%; N: 5.83%.

(3*R*,7a*R*)-3-((1*H*-indol-3-yl)methyl)-7a-(4’-hydroxy-[1,1’-biphenyl]-4-yl)tetrahydropyrrolo[2,1-*b*]oxazol-5(6*H*)-one (**7p**): Following the general procedure, to a solution of **7d** (0.050 g, 0.122 mmol) in dioxane (1.4 mL) was added Pd(PPh_3_)_2_Cl_2_ (0.004 g, 12.2 µmol), 1 M aq. sol. of Na_2_CO_3_ (370 µL), and 4-hydroxyphenylboronic acid (0.021 g, 0.148 mmol). Reaction time: 2 h. The compound was purified by flash chromatography (EtOAc/*n*-hexane 3:2) to afford the title compound as a white solid (0.044 g, 85%); m.p.: 223–225 °C; ^1^H NMR (300 MHz, CDCl_3_) δ 7.98 (s, 1H, NH), 7.62–7.43 (m, 7H, ArH), 7.34 (d, *J* = 7.8 Hz, 1H, ArH), 7.18 (t, *J* = 7.6 Hz, 1H, ArH), 7.10–7.05 (m, 2H, ArH), 6.95 (d, *J* = 8.5 Hz, 2H, ArH), 5.21 (s, 1H, OH), 4.68–4.51 (m, 1H, H-3), 4.20 (dd, *J* = 8.5, 7.8 Hz, 1H, H-2), 3.66 (dd, *J* = 8.6, 6.8 Hz, 1H, H-2), 3.13 (dd, *J* = 14.9, 6.3 Hz, 1H, indole-CH_2_), 2.98–2.76 (m, 1H, CH_2_), 2.75–2.46 (m, 3H, CH_2_, and indole-CH_2_), 2.33–2.24 (m, 1H, CH_2_); ^13^C NMR (75 MHz, (CD_3_)_2_SO) δ 179.5 (C=O), 157.2 (Cq), 140.8 (Cq), 139.9 (Cq), 136.0 (Cq), 130.1 (Cq), 127.7 (ArCH), 126.9 (Cq), 126.0 (ArCH), 125.5 (ArCH), 122.9 (ArCH), 121.0 (ArCH), 118.3 (ArCH), 117.9 (ArCH), 115.8 (ArCH), 111.4 (ArCH), 109.9 (Cq), 101.7 (C-7a), 72.3 (C-2), 55.3 (C-3), 40.4 (CH_2_), 32.8 (CH_2_), 29.8 (indole-CH_2_); Anal. Calcd. for C_27_H_24_N_2_O_3_·0.15H_2_O: C: 75.91%; H: 5.75%; N: 6.56%. Found C: 75.74%; H: 5.85%; N: 6.57%.

(3*R*,7a*R*)-3-((1*H*-indol-3-yl)methyl)-7a-(4’-(hydroxymethyl)-[1,1’-biphenyl]-4-yl)tetrahydropyrrolo[2,1-*b*]oxazol-5(6*H*)-one (**7q**): Following the general procedure, to a solution of **7d** (0.070 g, 0.170 mmol) in dioxane (2.0 mL) was added Pd(PPh_3_)_2_Cl_2_ (0.005 g, 17.0 µmol), 1 M aq. sol. of Na_2_CO_3_ (520 µL), and 4-(hydroxymethyl)phenylboronic acid (0.032 g, 0.208 mmol). Reaction time: 3 h. The compound was purified by flash chromatography (EtOAc/*n*-hexane 3:2) to afford the title compound as a pale yellow solid (0.053 g, 71%); m.p.: 213–215 °C; ^1^H NMR (300 MHz, CDCl_3_) δ 8.00 (s, 1H, NH), 7.65–7.44 (m, 9H, ArH), 7.33 (d, *J* = 8.0 Hz, 1H, ArH), 7.17 (t, *J* = 7.3 Hz, 1H, ArH), 7.11–7.02 (m, 2H, ArH), 4.77 (s, 2H, CH_2_), 4.67–4.53 (m, 1H, H-3), 4.19 (t, *J* = 8.0 Hz, 1H, H-2), 3.66 (t, *J* = 8.0 Hz, 1H, H-2), 3.11 (dd, *J* = 14.7, 6.0 Hz, 1H, indole-CH_2_), 2.95–2.78 (m, 1H, CH_2_), 2.69–2.47 (m, 3H, CH_2_, and indole-CH_2_), 2.38–2.20 (m, 1H, CH_2_); ^13^C NMR (75 MHz, CDCl_3_) δ 180.3 (C=O), 142.0 (Cq), 140.9 (Cq), 140.4 (Cq), 140.1 (Cq), 136.3 (Cq), 127.5 (ArCH), 127.4 (Cq), 127.3 (ArCH), 127.2 (ArCH), 125.8 (ArCH), 122.3 (ArCH), 122.2 (ArCH), 119.6 (ArCH), 119.0 (ArCH), 111.9 (Cq), 111.3 (ArCH), 102.5 (C-7a), 73.0 (C-2), 65.3 (CH_2_OH), 55.8 (C-3), 35.3 (CH_2_), 32.8 (CH_2_), 29.9 (indole-CH_2_); Anal. Calcd. (C_28_H_26_N_2_O_3_·0.40H_2_O): C: 75.44%; H: 6.07%; N: 6.29%. Found C: 75.18%; H: 6.21%; N: 6.14%.

(3*R*,7a*R*)-3-((1*H*-indol-3-yl)methyl)-7a-(3’-chloro-[1,1’-biphenyl]-4-yl)tetrahydropyrrolo[2,1-*b*]oxazol-5(6*H*)-one (**7r**): Following the general procedure, to a solution of **7d** (0.070 g, 0.170 mmol) in dioxane (2.0 mL) was added Pd(PPh_3_)_2_Cl_2_ (0.005 g, 17.0 µmol), 1 M aq. sol. of Na_2_CO_3_ (520 µL), and 3-chlorophenylboronic acid (0.033 g, 0.208 mmol). Reaction time: 4 h. The compound was purified by flash chromatography (EtOAc/*n*-hexane 1:1) to afford the title compound as a pale yellow solid (0.059 g, 78%); m.p.: 204–206 °C; ^1^H NMR (300 MHz, CDCl_3_) δ 7.98 (s, 1H, NH), 7.47 (t, *J* = 1.6 Hz, 1H, ArH), 7.43 (s, 4H, ArH), 7.36 (dt, *J* = 7.4 Hz, 1.6 Hz, 1H, ArH), 7.30 (d, *J* = 7.7 Hz, 1H, ArH), 7.25–7.17 (m, 3H, ArH), 7.06–7.00 (m, 1H, ArH), 6.95–6.91 (m, 2H, ArH), 4.51–4.42 (m, 1H, H-3), 4.05 (dd, *J* = 8.9, 6.9 Hz, 1H, H-2), 3.51 (dd, *J* = 8.8, 6.8 Hz, 1H, H-2), 2.95 (dd, *J* = 14.4, 6.6 Hz, 1H, indole-CH_2_), 2.80–2.66 (m, 1H, CH_2_), 2.53–2.34 (m, 3H, CH_2_, and indole-CH_2_), 2.22–2.08 (m, 1H, CH_2_); ^13^C NMR (75 MHz, CDCl_3_) δ 180.3 (C=O), 142.51 (Cq), 142.46 (Cq), 139.9 (Cq), 136.3 (Cq), 134.9 (Cq), 130.3 (ArCH), 127.8 (ArCH), 127.6 (ArCH), 127.6 (Cq), 127.5 (ArCH), 125.9 (ArCH), 125.4 (ArCH), 122.3 (ArCH), 119.6 (ArCH), 118.9 (ArCH), 111.7 (Cq), 111.3 (ArCH), 102.4 (C-7a), 72.9 (C-2), 55.7 (C-3), 35.2 (CH_2_), 32.8 (CH_2_), 29.8 (indole-CH_2_); Anal. Calcd. for C_27_H_23_ClN_2_O_2_: C: 73.21%; H: 5.23%; N: 6.32%. Found C: 72.91%; H: 5.70%; N: 6.24%.

(3*R*,7a*R*)-3-((1H-indol-3-yl)methyl)-7a-(3’,4’-dichloro-[1,1’-biphenyl]-4-yl)tetrahydropyrrolo[2,1-*b*]oxazol-5(6*H*)-one (**7s**): Following the general procedure, to a solution of **7d** (0.070 g, 0.170 mmol) in dioxane (2.0 mL) was added Pd(PPh_3_)_2_Cl_2_ (0.005 g, 17.0 µmol), 1 M aq. sol. of Na_2_CO_3_ (520 µL), and 3,4-dichlorophenylboronic acid (0.040 g, 0.208 mmol). Reaction time: 4 h. The compound was purified by flash chromatography (EtOAc/*n*-hexane 1:1) to afford the title compound as a pale yellow solid (0.069 g, 85%); m.p.: 176–178 °C; ^1^H NMR (300 MHz, CDCl_3_) δ 7.98 (s, 1H, NH), 7.71 (d, *J* = 2.1 Hz, 1H, ArH), 7.49 (s, 5H, ArH), 7.38 (dd, *J* = 8.4 Hz, 2.2 Hz, 2H, ArH), 7.27 (d, *J* = 8.1 Hz, 1H, ArH), 7.11 (d, *J* = 8.1 Hz, 1H, ArH), 7.06–6.97 (m, 2H, ArH), 4.65–4.45 (m, 1H, H-3), 4.14 (dd, *J* = 8.7, 7.4 Hz, 1H, H-2), 3.58 (dd, *J* = 8.8, 6.8 Hz, 1H, H-2), 3.02 (dd, *J* = 15.0, 6.6 Hz, 1H, indole-CH_2_), 2.87–2.76 (m, 1H, CH_2_), 2.65–2.38 (m, 3H, CH_2_,, and indole-CH_2_), 2.26–2.15 (m, 1H, CH_2_); ^13^C NMR (75 MHz, (CD_3_)_2_SO) δ 180.3 (C=O), 142.9 (Cq), 140.7 (Cq), 138.9 (Cq), 136.2 (Cq), 132.9 (Cq), 131.7 (Cq), 131.0 (ArCH), 128.9 (ArCH), 127.5 (Cq), 127.4 (ArCH), 126.3 (ArCH), 126.0 (ArCH), 122.3 (ArCH), 122.2 (ArCH), 119.6 (ArCH), 118.8 (ArCH), 111.7 (Cq), 111.3 (ArCH), 102.3 (C-7a), 72.8 (C-2), 55.8 (C-3), 35.2 (CH_2_), 32.9 (CH_2_), 29.9 (indole-CH_2_); Anal. Calcd. for C_27_H_22_Cl_2_N_2_O_2_: C: 67.93%; H: 4.65%; N: 5.87%. Found C: 67.96%; H: 4.90%; N: 5.73%. HRMS-ESI *m*/*z* calcd for C_27_H_22_Cl_2_N_2_O_2_: 476.1058, found 477.1143 ± 3.6 ppm [M+H]+.

(3*R*,7a*R*)-3-((1*H*-indol-3-yl)methyl)-7a-(4-(pyridin-4-yl)phenyl)tetrahydropyrrolo[2,1-*b*]oxazol-5(6*H*)-one (**7t**): Following the general procedure, to a solution of **7d** (0.070 g, 0.170 mmol) in dioxane (2.0 mL) was added Pd(PPh_3_)_2_Cl_2_ (0.005 g, 17.0 µmol), 1 M aq. sol. of Na_2_CO_3_ (520 µL), and 4-pyridinylboronic acid (0.026 g, 0.208 mmol). Reaction time: 2 h. The compound was purified by flash chromatography (EtOAc/*n*-hexane 3:1) to afford the title compound as a pale yellow solid (0.068 g, 97%); m.p.: 214–215 °C; ^1^H NMR (300 MHz, CDCl_3_) δ 8.69 (d, *J* = 5.6 Hz, 2H, ArH), 8.23 (s, 1H, NH), 7.63 (q, *J* = 8.4 Hz, 4H, ArH), 7.54 (d, *J* = 5.9 Hz, 2H, ArH), 7.43 (d, *J* = 7.8 Hz, 1H, ArH), 7.32 (d, *J* = 8.0 Hz, 1H, ArH), 7.17 (t, *J* = 7.4 Hz, 1H, ArH), 7.12–7.01 (m, 2H, ArH), 4.71–4.55 (m, 1H, H-3), 4.21 (t, *J* = 8.1 Hz, 1H, H-2), 3.65 (dd, *J* = 8.6, 7.0 Hz, 1H, H-2), 3.09 (dd, *J* = 14.7, 6.1 Hz, 1H, indole-CH_2_), 2.88 (ddd, *J* = 24.1, 12.1, 6.3 Hz, 1H, CH_2_), 2.72–2.45 (m, 3H, CH_2_, and indole-CH_2_), 2.37–2.20 (m, 1H, CH_2_); ^13^C NMR (75 MHz, CDCl_3_) δ 180.3 (C=O), 150.5 (ArCH), 147.9 (Cq), 144.0 (Cq), 138.2 (Cq), 136.3 (Cq), 127.5 (ArCH), 126.1 (ArCH), 122.4 (ArCH), 122.3 (ArCH), 121.8 (ArCH), 119.6 (ArCH), 118.9 (ArCH), 111.6 (Cq), 111.3 (ArCH), 102.3 (C-7a), 73.0 (C-2), 55.81 (C-3), 35.19 (CH_2_), 32.75 (CH_2_), 29.85 (indole-CH_2_). Anal. Calcd. for C_26_H_23_N_3_O_2_·0.20H_2_O: C: 75.59%; H: 5.72%; N: 10.17%. Found C: 75.90%; H: 5.81%; N: 9.73%.

(3*R*,7a*R*)-3-((1*H*-indol-3-yl)methyl)-7a-(4-(2,3-dihydrobenzo[*b*][1,4]dioxin-6-yl)phenyl)tetrahydropyrrolo[2,1-*b*]oxazol-5(6*H*)-one (**7u**): Following the general procedure, to a solution of **7d** (0.070 g, 0.170 mmol) in dioxane (2.0 mL) was added Pd(PPh_3_)_2_Cl_2_ (0.005 g, 17.0 µmol), 1 M aq. sol. of Na_2_CO_3_ (520 µL), and 1,4-benzodioxane-6-boronic acid (0.037 g, 0.208 mmol). Reaction time: 5 h. The compound was purified by flash chromatography (EtOAc/*n*-hexane 1:1) to afford the title compound as a pale yellow solid (0.022 g, 28%); m.p.: 286–288 °C; ^1^H NMR (300 MHz, CDCl_3_) δ 7.97 (s, 1H, NH), 7.56–7.44 (m, 5H, ArH), 7.33 (d, *J* = 8.0 Hz, 1H, ArH), 7.19–7.08 (m, 5H, ArH), 6.95 (d, *J* = 8.3 Hz, 1H, ArH), 4.64–4.54 (m, 1H, H-3), 4.31 (s, 4H, CH2), 4.18 (dd, *J* = 8.7, 7.5 Hz, 1H, H-2), 3.65 (dd, *J* = 8.8, 6.9 Hz, 1H, H-2), 3.11 (dd, *J* = 14.7, 6.1 Hz, 1H, indole-CH2), 2.93–2.82 (m, 1H, CH2), 2.67–2.45 (m, 3H, CH2, and indole-CH2), 2.37–2.19 (m, 1H, CH2); ^13^C NMR (75 MHz, CDCl_3_) δ 180.4 (C=O), 143.9 (Cq), 143.6 (Cq), 141.4 (Cq), 140.7 (Cq), 136.3 (Cq), 134.2 (Cq), 127.3 (Cq), 126.9 (ArCH), 125.4 (ArCH), 122.3 (ArCH), 122.0 (ArCH), 120.4 (ArCH), 119.4 (ArCH), 118.8 (ArCH), 117.6 (ArCH), 115.8 (ArCH), 111.8 (Cq), 111.2 (ArCH), 102.4 (C-7a), 72.9 (C-2), 64.7 (CH_2_), 55.7 (C-3), 35.2 (CH_2_), 32.9 (CH_2_), 29.9 (indole-CH_2_). Anal. Calcd. for C_29_H_26_N_2_O_4_: C: 74.66%; H: 5.68%; N: 6.00%. Found C: 74.65%; H: 5.70%; N: 5.67%.

### 3.2. Biological Assays

#### 3.2.1. Cytotoxicity Assays

The cytotoxicity was assessed in different cell lines with the endpoint MTT, using previously reported procedure [29,30,31]. The following cells were obtained from the American Type Culture Collection: human embryonic kidney epithelial cell line (HEK 293T, ATCC HBT-22™), breast cancer cell line (MDA-MB-231, ATCC HTB-26™), osteosarcoma cell line (MG-63, ATCC CRL-1427™), gastric adenocarcinoma cell line (AGS, ATTC CRL-1739TM), prostate cancer cell line (DU-145, ATTC HTB-81™), and lung carcinoma cell line (A-549, ATCC CCL-185™). All cell lines were seeded at 2 × 10^4^ cells/well with exception of A-549 cell line, which was seeded at 5 × 10^3^ cells/well.

#### 3.2.2. Caspase 3/7 Activity Assay

The activity of caspase 3/7 was determined by fluorimetric assay based on the hydrolysis of the peptide substrate acetyl-Asp-Glu-ValAsp-7-amido-4-methylcoumarin (Ac-DEVD-AMC) by caspase 3/7 using a previously reported procedure [32].

### 3.3. In Vitro Stability Assays

#### 3.3.1. Buffer and Human Plasma Stabilities for Compound **7s**

Human plasma was obtained from healthy volunteers and provided by Instituto Português do Sangue, Lisbon, Portugal. Buffer and human plasma stabilities were determined by standard methodology [33]. Specifically, human plasma was centrifuged (5 min, 2000× *g*, room temperature) and, then, diluted 50% in PBS buffer (pH 7.4). The reactions were initiated by the addition of a solution of compound **7s** (4 mM in DMSO, 25 µL) to 975 µL of plasma solution, at 37 °C, obtaining a final concentration of 100 µM. Solutions were stirred at 37 °C and 100 µL aliquots were collected at different time points: 0, 30, 60, 120, and 180 min (one additional aliquot was collected at 24 h). A cold reserpine solution (internal standard, 5 µM in acetonitrile, 300 µL) was then added to quench the reactions. Following centrifugation (10 min, 10,000× *g*, room temperature), the clear supernatants were stored at −20 °C until further analysis by HPLC-DAD. Assays were run in duplicate and procaine was used as a positive control for plasma stability. Additional control assays were conducted using PBS (pH 7.4) instead of a plasma solution. HPLC-DAD analysis was performed as previously described [34].

#### 3.3.2. Metabolic Stability for Compound **7s**

The evaluation of the metabolic stability of compound **7s** was conducted in human liver microsomes (GIBCO^TM^, 20 donors) by a previously reported procedure [35]. Specifically, for a total incubation volume of 1 mL, in 100 mM phosphate buffer at pH 7.4, **7s** (10 μM), human liver microsomes (0.8 mg protein/mL), NADPH (1 mM), and NADPH regeneration system (10 µL, Vivid^®^ Regeneration System, 100×) were used. Nevirapine was used as a positive control. Additional control incubations were performed in the absence of **7s** or NADPH, and using heat-denatured (90 °C, 15 min) microsomes. The resulting mixtures were incubated at 37 °C, and all assays were run in duplicate. Aliquots (50 µL) were collected at different time points (0, 5, 10, 20, 30, 40, 50, 60, 75, 90, 120, and 180 min and 24 h) and 50 µL of cold reserpine solution (2.5 µM in acetonitrile) was then added to quench the reactions. Following centrifugation (10 min, 10,000× *g*, 4 °C), the supernatants were stored at −20 °C until LC-MS and LC-HRMS/MS analysis.

#### 3.3.3. Half-Life *t*_1/2_ Determination

Samples from the metabolic stability assay were analyzed by LC-MS using the experimental conditions previously described [36]. The in vitro depletion half-life of **7s**, *t*_1/2_, was calculated using Equation (1), assuming that the compound follows a first-order kinetic trend (see Supplementary Materials Figure S2). The “slope” was determined from linear fitting of the natural logarithm of the concentration of drug remaining plotted against time.

(1) $$t_{1/2}=\frac{ln2}{slope}$$

The intrinsic clearance was calculated using Equation (2) [24,25]

(2) $$CL_{int\;}\;=\;\frac{0.693}{\;t_{\frac{1}{2}}\;}\;\times \;\frac{mL\;incubation}{mg\;microsomes}\;\times \;\frac{45\;mg\;microsomes}{g\;liver}\;\times \;\frac{26\;g\;liver}{Kg\;b.w.}$$

#### 3.3.4. Metabolite Identification

The 60 min aliquot was analyzed by LC-HRMS/MS, as previously described [36]. All spectra corresponding to metabolites were then manually checked. The mass deviation from the accurate mass of the identified metabolites remained below 5 ppm for the precursor and product ions. After their detection, structural characterization of the potential metabolites was based on tandem mass data (see Supplementary Materials Figures S2–S4).

## 4. Conclusions

A series of enantiopure tryptophanol-derived bicyclic lactams was prepared, and its antiproliferative activity was evaluated in AGS cells. From the first screening emerged compound **7c**, a (*R*)-tryptophanol derivative with a *para*-chloro phenyl substituent, which was selected for further optimization. Introduction of an additional di-halogenated aromatic ring in **7c** structure led to two derivatives 2.3- to 2.7-fold more active in AGS cells. These compounds also showed moderate activity in prostate cancer cells, representing useful hit compounds for further optimization in this type of cancer. More importantly, additional assays with the two compounds showed they are not toxic in normal HEK 293T cells, and that the antiproliferative activity in AGS cells occurs through apoptosis. Stability studies with the most potent derivative, compound **7s**, showed that the compound is stable in PBS and human plasma. Moreover, incubation assays in human liver microsomes, followed by LC-HRMS/MS analysis, showed that this compound is moderately metabolically stable and that the major metabolites stem from mono-hydroxylation of the indole ring, which is not anticipated to be a toxicity red flag alert.

## Figures and Tables

**Table 1:** Screening of (*R*) and (*S*)-tryptophanol-derived oxazolopyrrolidone lactams **7a**–**g** and **8a**–**g** in AGS cell line.

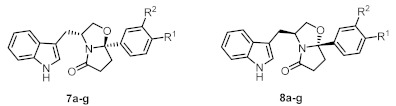			
Compound	R^1^	R^2^	% CV at 100 µM ^1^
**7a**	H	H	50 ± 5
**7b**	F	H	50 ± 8
**7c**	Cl	H	6 ± 1
**7d**	Br	H	14 ± 3
**7e**	CH_3_	H	14 ± 2
**7f**	OCH_3_	H	18 ± 2
**7g**	SO_2_CH_3_	H	94 ± 12
**8a**	H	H	58 ± 4
**8b**	F	H	22 ± 4
**8c**	Cl	H	11 ± 1
**8d**	Br	H	25 ± 4
**8e**	CH_3_	H	60 ± 3
**8f**	OCH_3_	H	30 ± 4
**8g**	SO_2_CH_3_	H	91 ± 11

^1^ Each % of CV (cell viability) value is the mean ± SD of triplicate of at least two different experiments. % CV determined by the MTT method after 48 h of compounds’ incubation.

**Table 2:** Screening of (*R*)-tryptophanol-derived oxazolopyrrolidone lactams **7c**, **7h**–**u**, and **7j’** in AGS cell line.

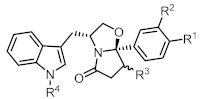					
Compound	R^1^	R^2^	R^3^	R^4^	% CV at 50 µM ^1^
**7c**	Cl	H	H	H	11 ± 1
**7h**	CH_3_	Cl	H	H	11 ± 2
**7i**	OCH_3_	F	H	H	84 ± 7
**7j**	Cl	H	(*S*)-CH_3_	H	8 ± 0
**7j’**	Cl	H	(*R*)-CH_3_	H	81 ± 3
**7k**	Cl	H	H	CH_2_CH_3_	40 ± 5
**7l**	Cl	H	H	COCH_3_	63 ± 13
**7m**	Cl	H	H	CO_2_C(CH_3_)_3_	61 ± 9
**7n**	*p*-Cl-Ph	H	H	H	65 ± 13
**7o**	*p*-CF_3_-Ph	H	H	H	7 ± 1
**7p**	*p*-OH-Ph	H	H	H	56 ± 1
**7q**	*p*-CH_2_OH-Ph	H	H	H	55 ± 1
**7r**	*m*-Cl-Ph	H	H	H	17 ± 1
**7s**	3,4-Cl-Ph	H	H	H	8 ± 1
**7t**	pyridine	H	H	H	75 ± 14
**7u**	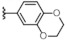	H	H	H	67 ± 6

^1^ Each % of CV (cell viability) value is the mean ± SD of triplicate of at least two different experiments. % CV determined by the MTT method after 48 h of compounds’ incubation.

**Table 3:** IC_50_ values ^1^ in cancer cell lines of selected compounds.

Compound	AGS IC_50_ (µM)	MDA-MB-231 IC_50_ (µM)	A-549 IC_50_ (µM)	DU-145 IC_50_ (µM)	MG-63 IC_50_ (µM)	HEK 293T IC_50_ (µM)
**7c**	(3.4 ± 0.2) × 10	-	-	-	-	-
**7j**	(2.8 ± 0.4) × 10	-	-	-	-	-
**7o**	(1.5 ± 0.6) × 10	(2.8 ± 0.2) × 10	(6.3 ± 1.8) × 10	(2.4 ± 0.5) × 10	26.8 ± 0.4	(5.2 ± 0.2) × 10
**7s**	(1.3 ± 0.4) × 10	(6.8 ± 0.6) × 10	(8.8 ± 1.7) × 10	(2.1 ± 1.2) × 10	(5.6 ± 0.7) × 10	(11.7 ± 0.6) × 10

^1^ IC_50_ values determined by the MTT method after 48 h of compounds’ incubation. Each IC_50_ value is the mean ± SD of, at least, three independent experiments with six replicates each.

## Data Availability

CCDC 2050433-2050435 contains the supplementary crystallographic data for this paper. These data are provided free of charge by The Cambridge Crystallographic Data Centre.

## Supplementary Materials

The following are available online at https://www.mdpi.com/1424-8247/14/3/208/s1: crystallographic information for compounds **7j**, **7j’**, and **8b**; LC-HRMS/MS data for compound **7s** and its metabolites; NMR spectra of compounds **7h**, **7j**, **7j’**, **7o**, and **7s**.

## References

[1] Bray F., Ferlay J., Soerjomataram I., Siegel R.L., Torre L.A., Jemal A. (2018). Global cancer statistics 2018: GLOBOCAN estimates of incidence and mortality worldwide for 36 cancers in 185 countries. CA Cancer J. Clin..

[2] https://www.who.int/news-room/fact-sheets/detail/cancer.

[3] Rawla P., Barsouk A. (2019). Epidemiology of gastric cancer: Global trends, risk factors and prevention. Prz. Gastroenterol..

[4] Digklia A., Wagner A.D. (2016). Advanced gastric cancer: Current treatment landscape and future perspectives. World J. Gastroenterol..

[5] Tomasello G., Ghidini M., Liguigli W., Ratti M., Toppo L., Passalacqua R. (2016). Targeted therapies in gastric cancer treatment: Where we are and where we are going. Investig. New Drugs.

[6] Lazăr D.C., Tăban S., Cornianu M., Faur A., Goldiş A. (2016). New advances in targeted gastric cancer treatment. World J. Gastroenterol..

[7] Sitarz R., Skierucha M., Mielko J., Offerhaus G.J.A., Maciejewski R., Polkowski W.P. (2018). Gastric cancer: Epidemiology, prevention, classification, and treatment. Cancer Manag. Res..

[8] Marin J.J., Al-Abdulla R., Lozano E., Briz O., Bujanda L., Banales J.M., Macias R.I. (2016). Mechanisms of Resistance to Chemotherapy in Gastric Cancer. Anticancer Agents Med. Chem..

[9] Sravanthi T.V., Manju S.L. (2016). Indoles—A promising scaffold for drug development. Eur. J. Pharm. Sci..

[10] Sharma V., Kumar P., Pathak D. (2010). Biological Importance of the Indole Nucleus in Recent Years: A Comprehensive Review. J. Heterocycl. Chem..

[11] Wan Y., Li Y., Yan C., Yan M., Tang Z. (2019). Indole: A privileged scaffold for the design of anti-cancer agents. Eur. J. Med. Chem..

[12] Soares J., Raimundo L., Pereira N.A., dos Santos D.J., Pérez M., Queiroz G., Leão M., Santos M.M.M., Saraiva L. (2015). A tryptophanol-derived oxazolopiperidone lactam is cytotoxic against tumors via inhibition of p53 interaction with murine double minute proteins. Pharmacol. Res..

[13] Soares J., Raimundo L., Pereira N.A., Monteiro Â., Gomes S., Bessa C., Pereira C., Queiroz G., Bisio A., Fernandes J. (2016). Reactivation of wild-type and mutant p53 by tryptophanolderived oxazoloisoindolinone SLMP53-1, a novel anticancer small-molecule. Oncotarget.

[14] Soares J., Espadinha M., Raimundo L., Ramos H., Gomes A.S., Gomes S., Loureiro J.B., Inga A., Reis F., Gomes C. (2017). DIMP53-1: A novel small-molecule dual inhibitor of p53-MDM2/X interactions with multifunctional p53-dependent anticancer properties. Mol. Oncol..

[15] Raimundo L., Espadinha M., Soares J., Loureiro J.B., Alves M.G., Santos M.M.M., Saraiva L. (2018). Improving anticancer activity towards colon cancer cells with a new p53-activating agent. Br. J. Pharmacol..

[16] Gomes S., Bosco B., Loureiro J.B., Ramos H., Raimundo L., Soares J., Nazareth N., Barcherini V., Domingues L., Oliveira C. (2019). SLMP53-2 Restores Wild-Type-Like Function to Mutant p53 through Hsp70: Promising Activity in Hepatocellular Carcinoma. Cancers.

[17] Barcherini V., Almeida J., Lopes E.A., Wang M., Magalhães E., Silva D., Mori M., Wang S., Saraiva L., Santos M.M.M. (2020). Potency and Selectivity Optimization of Tryptophanol-Derived Oxazoloisoindolinones: Novel p53 Activators in Human Colorectal Cancer. ChemMedChem.

[18] Jiang L.J., Lv S.M., Cheng C., Dong L., Li Y., Yin S.F. (2015). Synthesis and Antitumor Activity of a Novel Series of Helicid-Pyrrolidone Derivatives. Chem. Nat. Compd..

[19] Li J., Wu Y., Guo Z., Zhuang C., Yao J., Dong G., Yu Z., Min X., Wang S., Liu Y. (2014). Discovery of 1-arylpyrrolidone derivatives as potent p53-MDM2 inhibitors based on molecule fusing strategy. Bioorg. Med. Chem. Lett..

[20] Pereira N.A., Monteiro Â., Machado M., Gut J., Molins E., Perry M.J., Dourado J., Moreira R., Rosenthal P.J., Prudêncio M. (2015). Enantiopure Indolizinoindolones with in vitro Activity against Blood- and Liver-Stage Malaria Parasites. ChemMedChem.

[21] Espadinha M., Viejo L., Lopes R.M.R.M., Herrera-Arozamena C., Molins E., Dos Santos D.J.V.A., Gonçalves L., Rodríguez-Franco M.I., Ríos C.L., Santos M.M.M. (2020). Identification of tetracyclic lactams as NMDA receptor antagonists with potential application in neurological disorders. Eur. J. Med. Chem..

[22] Shukla S.J., Huang R., Austin C.P., Xia M. (2010). The future of toxicity testing: A focus on in vitro methods using a quantitative high-throughput screening platform. Drug Discov. Today.

[23] Cerny M.A., Kalgutkar A.S., Obach R.S., Sharma R., Spracklin D.K., Walker G.S. (2020). Effective Application of Metabolite Profiling in Drug Design and Discovery. J. Med. Chem..

[24] McNaney C.A., Drexler D.M., Hnatyshyn S.Y., Zvyaga T.A., Knipe J.O., Belcastro J.V., Sanders M. (2008). An automated liquid chromatography-mass spectrometry process to determine metabolic stability half-life and intrinsic clearance of drug candidates by substrate depletion. Assay Drug Dev. Technol..

[25] Słoczyńska K., Gunia-Krzyżak A., Koczurkiewicz P., Wójcik-Pszczoła K., Żelaszczyk D., Popiół J., Pękala E. (2019). Metabolic stability and its role in the discovery of new chemical entities. Acta Pharm..

[26] Gillam E.M., Notley L.M., Cai H., De Voss J.J., Guengerich F.P. (2000). Oxidation of indole by cytochrome P450 enzymes. Biochemistry.

[27] Wang K., Burton R., Anderson S. (2015). Bioactivation of Indoles by Human Liver Microsomes with Glutathione as Trapping Agent: Formation of Novel Cyclic Cysteine and Cysteine-Glycine Adducts. FASEB J..

[28] Bailey P.D., Collier I.D., Hollinshead S.P., Moore M.H., Morgan K.M., Smith D.I., Vernon J.M. (1997). New asymmetric route to bridged indole alkaloids: Formal enantiospecific syntheses of (−)-suaveoline, (−)-raumacline and (−)-Nb methylraumacline. J. Chem. Soc. Perkin Trans..

[29] Mosmann T. (1983). Rapid colorimetric assay for cellular growth and survival: Application to proliferation and cytotoxicity assays. J. Immunol. Methods.

[30] Cadete A., Figueiredo L., Lopes R., Calado C.C., Almeida A.J., Gonçalves L.M. (2012). Development and characterization of a new plasmid delivery system based on chitosan-sodium deoxycholate nanoparticles. Eur. J. Pharm. Sci..

[31] Monteiro Â., Gonçalves L.M., Santos M.M.M. (2014). Synthesis of novel spiropyrazoline oxindoles and evaluation of cytotoxicity in cancer cell lines. Eur. J. Med. Chem..

[32] Marto J., Ascenso A., Gonçalves L.M., Gouveia L.F., Manteigas P., Pinto P., Oliveira E., Almeida A.J., Ribeiro H.M. (2016). Melatonin-based pickering emulsion for skin’s photoprotection. Drug Deliv..

[33] Hartman D.A. (2003). Determination of the stability of drugs in plasma. Curr. Protoc. Pharmacol..

[34] Martins I.L., Nunes J., Charneira C., Morello J., Pereira S.A., Telo J.P., Marques M.M., Antunes A.M.M. (2018). The first-line antiepileptic drug carbamazepine: Reaction with biologically relevant free radicals. Free Radic. Biol. Med..

[35] Lopes B.T., Caldeira M.J., Gaspar H., Antunes A.M.M. (2021). Metabolic profile of four selected cathinones in microsome incubations: Identification of Phase I and II metabolites by Liquid Chromatography High Resolution Mass Spectrometry. Front. Chem..

[36] Godinho A.L.A., Martins I.L., Nunes J., Charneira C., Grilo J., Silva D.M., Pereira S.A., Soto K., Oliveira M.C., Marques M.M. (2018). High resolution mass spectrometry-based methodologies for identification of Etravirine bioactivation to reactive metabolites: In vitro and in vivo approaches. Eur. J. Pharm. Sci..

