# Supplementary material for: Tryptophanol-Derived Oxazolopyrrolidone Lactams as Potential Anticancer Agents against Gastric Adenocarcinoma
Source: Pharmaceuticals (Basel). 2021 Mar 2;14(3):208. doi: 10.3390/ph14030208 (PMC8001353; doi:10.3390/ph14030208)

Supporting Information

Tryptophanol-derived oxazolopyrrolidone lactams as potential anticancer agents against gastric adenocarcinoma

Margarida Espadinha[a], Valentina Barcherini[a], Lídia M. Gonçalves[a], Elies Molins[b], Alexandra M. M. Antunes[c], Maria M. M. Santos[a,*]

[a] Research Institute for Medicines (iMed.ULisboa), Faculty of Pharmacy, Universidade de Lisboa, Av. Prof. Gama Pinto, 1649-003 Lisboa, Portugal

[b] Institut de Ciència de Materials de Barcelona (ICMAB-CSIC), Campus UAB, 08193 Bellaterra, Catalonia, Spain

[c] Centro de Química Estrutural, Instituto Superior Técnico, ULisboa, 1049-001 Lisboa, Portugal

Table of contents:

X-ray crystallographic data for compounds **7j**, **7j'** and **8b** 1

LC-HRMS/MS data for compound **7s** and its metabolites 31

^{1}H NMR and ^{13}C NMR spectra 35

LC-MS data 56

X-ray crystallographic data for compound 7j

Table S1. Crystal data and structure refinement for **7j**.

Identification code	Jb156	
Empirical formula	$C_{22}H_{21}ClN_2O_2$	
Formula weight	380.86	
Temperature	294(2) K	
Wavelength	0.71073 Å	
Crystal system	Monoclinic	
Space group	P 21	
Unit cell dimensions	a = 9.6271(19) Å	a= 90°.
	b = 9.2727(19) Å	b= 103.725(4)°.
	c = 11.535(2) Å	g = 90°.
Volume	1000.3(3) $Å^3$	
Z	2	
Density (calculated)	1.264 Mg/m^3	
Absorption coefficient	0.210 mm^{-1}	
F(000)	400	
Crystal size	0.210 x 0.210 x 0.080 mm^3	
Theta range for data collection	1.817 to 28.357°.	
Index ranges	-12<=h<=12, -12<=k<=12, -15<=l<=15	
Reflections collected	21809	
Independent reflections	4999 [R(int) = 0.1121]	
Completeness to theta = 25.242°	100.0 %	
Refinement method	Full-matrix least-squares on F^2	
Data / parameters	4999 / 245	

Goodness-of-fit on F^2	0.956
Final R indices [I>2sigma(I)]	R1 = 0.0568, wR2 = 0.0897
R indices (all data)	R1 = 0.1797, wR2 = 0.1213
Absolute structure parameter	0.02(7)
Largest diff. peak and hole	0.129 and -0.167 e.Å^{-3}

Table S2. Atomic coordinates (x 10^4) and equivalent isotropic displacement parameters (Å^{2}x 10^3) for **7j**. U(eq) is defined as one third of the trace of the orthogonalized U^{ij} tensor.

	x	y	z	U(eq)
Cl(1)	1756(2)	1271(2)	-801(2)	121(1)
O(1)	2561(4)	7442(4)	2230(3)	76(1)
O(5)	6047(3)	6188(5)	5157(3)	91(1)
N(1A)	-910(5)	5680(5)	5659(4)	79(2)
N(4)	4104(4)	6113(5)	3583(3)	57(1)
C(2)	1809(6)	7002(6)	3098(5)	81(2)
C(2A)	-158(6)	5080(6)	4906(6)	74(2)
C(3)	2927(4)	6389(6)	4157(4)	56(1)
C(3A)	1252(5)	5409(5)	5290(5)	60(1)
C(3B)	1396(5)	6253(6)	6353(4)	53(1)
C(4A)	2536(6)	6926(5)	7137(4)	62(1)
C(5)	5509(5)	6270(7)	4087(5)	68(2)
C(5A)	2295(6)	7681(6)	8090(5)	74(2)
C(6)	6228(6)	6511(7)	3084(5)	85(2)
C(6A)	915(7)	7814(7)	8256(5)	79(2)
C(7)	5051(6)	7079(6)	2034(5)	76(2)
C(7A)	3689(5)	6419(6)	2316(4)	60(1)
C(7B)	-231(6)	7176(6)	7508(5)	75(2)
C(8A)	18(5)	6407(6)	6557(5)	62(1)
C(30)	2433(5)	5051(6)	4700(5)	64(2)
C(70)	5020(7)	8713(7)	1967(6)	120(3)
C(71)	3157(5)	5104(6)	1573(5)	58(1)
C(72)	2119(6)	5247(7)	513(5)	81(2)

C(73)	1697(7)	4068(9)	-214(5)	93(2)
C(74)	2312(6)	2774(8)	103(5)	72(2)
C(75)	3335(6)	2588(6)	1136(6)	79(2)
C(76)	3751(5)	3773(7)	1871(5)	72(2)

Table S3. Bond lengths [Å] and angles [°] for **7j**.

Cl(1)-C(74)	1.747(6)
O(1)-C(2)	1.427(6)
O(1)-C(7A)	1.428(6)
O(5)-C(5)	1.222(5)
N(1A)-C(2A)	1.374(6)
N(1A)-C(8A)	1.374(6)
N(4)-C(5)	1.347(6)
N(4)-C(7A)	1.449(5)
N(4)-C(3)	1.464(5)
C(2)-C(3)	1.533(6)
C(2A)-C(3A)	1.359(7)
C(3)-C(30)	1.517(7)
C(3A)-C(3B)	1.433(6)
C(3A)-C(30)	1.496(6)
C(3B)-C(4A)	1.393(6)
C(3B)-C(8A)	1.408(6)
C(4A)-C(5A)	1.369(6)
C(5)-C(6)	1.500(6)
C(5A)-C(6A)	1.393(7)
C(6)-C(7)	1.541(7)
C(6A)-C(7B)	1.364(7)
C(7)-C(70)	1.517(8)
C(7)-C(7A)	1.549(7)
C(7A)-C(71)	1.508(7)
C(7B)-C(8A)	1.376(7)

C(71)-C(76)	1.370(7)
C(71)-C(72)	1.390(7)
C(72)-C(73)	1.379(8)
C(73)-C(74)	1.350(8)
C(74)-C(75)	1.364(7)
C(75)-C(76)	1.388(7)
C(2)-O(1)-C(7A)	105.4(4)
C(2A)-N(1A)-C(8A)	109.3(4)
C(5)-N(4)-C(7A)	115.0(4)
C(5)-N(4)-C(3)	126.2(4)
C(7A)-N(4)-C(3)	111.4(4)
O(1)-C(2)-C(3)	106.7(4)
C(3A)-C(2A)-N(1A)	109.8(5)
N(4)-C(3)-C(30)	113.3(4)
N(4)-C(3)-C(2)	100.2(4)
C(30)-C(3)-C(2)	113.8(4)
C(2A)-C(3A)-C(3B)	106.8(4)
C(2A)-C(3A)-C(30)	127.6(5)
C(3B)-C(3A)-C(30)	125.5(4)
C(4A)-C(3B)-C(8A)	118.3(5)
C(4A)-C(3B)-C(3A)	134.5(4)
C(8A)-C(3B)-C(3A)	107.1(4)
C(5A)-C(4A)-C(3B)	119.6(5)
O(5)-C(5)-N(4)	124.8(5)
O(5)-C(5)-C(6)	128.7(5)
N(4)-C(5)-C(6)	106.4(5)
C(4A)-C(5A)-C(6A)	120.3(5)

C(5)-C(6)-C(7)	105.6(4)
C(7B)-C(6A)-C(5A)	122.0(6)
C(70)-C(7)-C(6)	112.5(5)
C(70)-C(7)-C(7A)	113.5(5)
C(6)-C(7)-C(7A)	101.6(4)
O(1)-C(7A)-N(4)	102.9(4)
O(1)-C(7A)-C(71)	110.3(4)
N(4)-C(7A)-C(71)	113.0(5)
O(1)-C(7A)-C(7)	113.0(5)
N(4)-C(7A)-C(7)	104.1(4)
C(71)-C(7A)-C(7)	113.0(4)
C(6A)-C(7B)-C(8A)	117.5(5)
N(1A)-C(8A)-C(7B)	130.6(5)
N(1A)-C(8A)-C(3B)	107.1(5)
C(7B)-C(8A)-C(3B)	122.3(5)
C(3A)-C(30)-C(3)	110.7(4)
C(76)-C(71)-C(72)	118.5(5)
C(76)-C(71)-C(7A)	121.4(5)
C(72)-C(71)-C(7A)	119.9(5)
C(73)-C(72)-C(71)	120.4(6)
C(74)-C(73)-C(72)	119.5(6)
C(73)-C(74)-C(75)	121.9(5)
C(73)-C(74)-Cl(1)	119.7(5)
C(75)-C(74)-Cl(1)	118.4(6)
C(74)-C(75)-C(76)	118.5(6)
C(71)-C(76)-C(75)	121.1(5)

Table S4. Anisotropic displacement parameters ($Å^2$x 10^3) for **7j**. The anisotropic displacement factor exponent takes the form: $-2p^2[h^2a^{*2}U^{11} + ... + 2 h k a^* b^* U^{12}]$

	U^{11}	U^{22}	U^{33}	U^{23}	U^{13}	U^{12}
Cl(1)	101(1)	137(2)	134(2)	-76(1)	44(1)	-41(1)
O(1)	83(3)	62(3)	89(3)	5(2)	33(2)	19(2)
O(5)	47(2)	141(4)	83(3)	-17(3)	12(2)	-8(3)
N(1A)	46(3)	92(4)	104(4)	-1(3)	32(3)	-6(3)
N(4)	49(3)	66(3)	59(3)	-11(2)	19(2)	-2(2)
C(2)	76(4)	86(5)	83(4)	-9(3)	25(4)	22(3)
C(2A)	54(4)	84(4)	87(4)	-19(3)	21(3)	-11(3)
C(3)	42(3)	63(3)	65(3)	-16(3)	19(2)	1(3)
C(3A)	41(3)	65(4)	75(4)	-8(3)	19(3)	-5(3)
C(3B)	44(3)	51(3)	67(3)	6(3)	19(2)	5(3)
C(4A)	60(4)	62(4)	65(3)	2(3)	19(3)	2(3)
C(5)	47(3)	84(4)	78(4)	-19(4)	23(3)	-4(3)
C(5A)	82(4)	71(4)	71(4)	-2(3)	21(3)	1(3)
C(6)	61(3)	102(5)	101(4)	-12(4)	40(3)	-12(4)
C(6A)	97(5)	69(4)	79(4)	4(3)	38(4)	19(4)
C(7)	87(4)	74(5)	76(4)	-10(3)	33(4)	-25(3)
C(7A)	64(3)	55(3)	65(3)	-3(3)	23(3)	5(3)
C(7B)	69(4)	81(4)	87(4)	14(4)	43(4)	22(3)
C(8A)	49(3)	64(4)	76(4)	11(4)	23(3)	9(3)
C(30)	49(3)	67(4)	81(4)	-16(3)	22(3)	-6(3)
C(70)	142(7)	67(5)	155(7)	-4(5)	42(5)	-30(4)
C(71)	50(3)	63(4)	64(4)	-1(3)	21(3)	0(3)

C(72)	90(5)	86(5)	64(4)	5(4)	11(4)	16(4)
C(73)	97(5)	113(6)	64(4)	-11(5)	10(4)	-2(5)
C(74)	66(4)	88(5)	68(4)	-38(4)	26(3)	-20(4)
C(75)	66(4)	61(4)	113(5)	-19(4)	26(4)	-1(3)
C(76)	51(3)	71(4)	89(4)	-15(4)	6(3)	5(3)

Table S5. Hydrogen bonds for **7j** [Å and °].

D-H...A	d(D-H)	d(H...A)	d(D...A)	<(DHA)
N(1A)-H(1A)...O(5)#1	0.86	2.07	2.887(6)	159.2

Symmetry transformations used to generate equivalent atoms:
#1 x-1,y,z

X-ray crystallographic data for compound 7j'

Table S6. Crystal data and structure refinement for **7j'**.

Identification code	Jb155	
Empirical formula	$C_{22}H_{21}ClN_2O_2$	
Formula weight	380.86	
Temperature	294(2) K	
Wavelength	0.71073 Å	
Crystal system	Monoclinic	
Space group	P 21	
Unit cell dimensions	a = 9.7065(10) Å	a= 90°.
	b = 7.9809(8) Å	b= 105.143(2)°.
	c = 13.0587(13) Å	g = 90°.
Volume	976.49(17) Å^3	
Z	2	
Density (calculated)	1.295 Mg/m^3	
Absorption coefficient	0.215 mm^{-1}	
F(000)	400	
Crystal size	0.15 x 0.15 x 0.08 mm^3	
Theta range for data collection	1.615 to 28.332°.	
Index ranges	-12<=h<=12, -4<=k<=10, -17<=l<=17	
Reflections collected	7895	
Independent reflections	3487 [R(int) = 0.0544]	
Completeness to theta = 25.242°	100.0 %	
Refinement method	Full-matrix least-squares on F^2	
Data / restraints / parameters	3487 / 1 / 245	

Goodness-of-fit on F^2	0.929
Final R indices [I>2sigma(I)]	R1 = 0.0503, wR2 = 0.0916
R indices (all data)	R1 = 0.1253, wR2 = 0.1129
Absolute structure parameter	0.02(13)
Largest diff. peak and hole	0.129 and -0.144 e.Å^{-3}

Table S7. Atomic coordinates (x 10^4) and equivalent isotropic displacement parameters (Å^{2}x 10^3) for **7j'**. U(eq) is defined as one third of the trace of the orthogonalized U^{ij} tensor.

	x	y	z	U(eq)
Cl(1)	8366(2)	1475(2)	10416(1)	113(1)
O(1)	7435(3)	8515(4)	7592(2)	63(1)
O(5)	3909(3)	6644(5)	5030(2)	67(1)
N(1A)	10857(3)	6321(5)	4592(2)	57(1)
N(4)	5902(3)	6892(5)	6405(2)	48(1)
C(2)	8205(4)	7903(6)	6863(3)	62(1)
C(2A)	10127(4)	5828(6)	5298(3)	55(1)
C(3)	7058(4)	7213(5)	5907(3)	50(1)
C(3A)	8699(4)	6033(5)	4892(3)	48(1)
C(3B)	8526(4)	6692(6)	3849(3)	47(1)
C(4A)	7349(4)	7189(6)	3036(3)	63(1)
C(5)	4489(4)	6892(6)	5969(3)	55(1)
C(5A)	7571(6)	7779(7)	2108(4)	83(2)
C(6)	3783(4)	7269(7)	6856(3)	67(1)
C(6A)	8952(6)	7893(7)	1970(4)	81(2)
C(7)	4959(4)	8161(6)	7687(3)	65(1)
C(7A)	6320(4)	7356(6)	7526(3)	52(1)
C(7B)	10122(5)	7437(6)	2744(3)	61(1)
C(8A)	9899(4)	6849(5)	3684(3)	47(1)
C(30)	7532(4)	5652(6)	5422(3)	55(1)
C(70)	4765(5)	8207(9)	8808(3)	99(2)
C(71)	6849(4)	5840(6)	8234(3)	53(1)
C(72)	7793(5)	6076(7)	9222(3)	71(1)

C(73)	8248(5)	4717(8)	9887(4)	76(2)
C(74)	7775(5)	3167(7)	9564(4)	71(1)
C(75)	6865(5)	2894(7)	8583(4)	73(1)
C(76)	6414(5)	4230(7)	7923(4)	64(1)

Table S8. Bond lengths [Å] and angles [°] for **7j’**.

Cl(1)-C(74)	1.749(5)
O(1)-C(7A)	1.409(5)
O(1)-C(2)	1.440(4)
O(5)-C(5)	1.225(4)
N(1A)-C(2A)	1.358(4)
N(1A)-C(8A)	1.369(4)
N(4)-C(5)	1.341(4)
N(4)-C(3)	1.459(4)
N(4)-C(7A)	1.461(5)
C(2)-C(3)	1.541(5)
C(2A)-C(3A)	1.358(5)
C(3)-C(30)	1.522(6)
C(3A)-C(3B)	1.429(5)
C(3A)-C(30)	1.504(5)
C(3B)-C(4A)	1.398(5)

C(3B)-C(8A)	1.410(5)
C(4A)-C(5A)	1.369(6)
C(5)-C(6)	1.522(5)
C(5A)-C(6A)	1.402(7)
C(6)-C(7)	1.530(5)
C(6A)-C(7B)	1.358(6)
C(7)-C(70)	1.524(5)
C(7)-C(7A)	1.532(5)
C(7A)-C(71)	1.528(6)
C(7B)-C(8A)	1.383(5)
C(71)-C(76)	1.380(6)
C(71)-C(72)	1.386(5)
C(72)-C(73)	1.388(7)
C(73)-C(74)	1.349(8)
C(74)-C(75)	1.370(6)
C(75)-C(76)	1.370(6)

C(7A)-O(1)-C(2)	105.0(3)
C(2A)-N(1A)-C(8A)	108.7(3)
C(5)-N(4)-C(3)	129.2(3)
C(5)-N(4)-C(7A)	114.2(3)
C(3)-N(4)-C(7A)	110.9(3)
O(1)-C(2)-C(3)	105.5(3)
N(1A)-C(2A)-C(3A)	111.1(3)
N(4)-C(3)-C(30)	113.1(3)
N(4)-C(3)-C(2)	100.3(3)
C(30)-C(3)-C(2)	113.4(3)
C(2A)-C(3A)-C(3B)	105.6(3)
C(2A)-C(3A)-C(30)	127.7(4)
C(3B)-C(3A)-C(30)	126.7(3)
C(4A)-C(3B)-C(8A)	118.5(4)
C(4A)-C(3B)-C(3A)	134.2(4)
C(8A)-C(3B)-C(3A)	107.3(3)
C(5A)-C(4A)-C(3B)	118.9(4)

O(5)-C(5)-N(4)	125.2(4)
O(5)-C(5)-C(6)	127.9(4)
N(4)-C(5)-C(6)	107.0(3)
C(4A)-C(5A)-C(6A)	120.9(4)
C(5)-C(6)-C(7)	103.3(3)
C(7B)-C(6A)-C(5A)	121.9(4)
C(70)-C(7)-C(6)	115.8(4)
C(70)-C(7)-C(7A)	118.0(4)
C(6)-C(7)-C(7A)	102.6(3)
O(1)-C(7A)-N(4)	103.6(3)
O(1)-C(7A)-C(71)	110.6(3)
N(4)-C(7A)-C(71)	111.6(4)
O(1)-C(7A)-C(7)	113.1(4)
N(4)-C(7A)-C(7)	102.7(3)
C(71)-C(7A)-C(7)	114.5(3)
C(6A)-C(7B)-C(8A)	117.2(4)
N(1A)-C(8A)-C(7B)	130.2(3)

N(1A)-C(8A)-C(3B)	107.2(3)
C(7B)-C(8A)-C(3B)	122.6(3)
C(3A)-C(30)-C(3)	111.8(3)
C(76)-C(71)-C(72)	118.6(5)
C(76)-C(71)-C(7A)	122.0(4)
C(72)-C(71)-C(7A)	119.5(4)
C(71)-C(72)-C(73)	120.1(5)
C(74)-C(73)-C(72)	119.6(4)
C(73)-C(74)-C(75)	121.5(5)
C(73)-C(74)-Cl(1)	118.8(4)
C(75)-C(74)-Cl(1)	119.7(5)
C(76)-C(75)-C(74)	119.1(5)
C(75)-C(76)-C(71)	121.1(4)

Table S9. Anisotropic displacement parameters ($Å^2 x 10^3$) for **7j'**. The anisotropic displacement factor exponent takes the form: $-2p^2[h^2a^{*2}U^{11} + ... + 2 h k a^* b^* U^{12}]$

	U^{11}	U^{22}	U^{33}	U^{23}	U^{13}	U^{12}
Cl(1)	104(1)	103(1)	134(1)	58(1)	34(1)	32(1)
O(1)	75(2)	57(2)	60(2)	-4(2)	26(2)	-17(2)
O(5)	49(1)	83(2)	66(2)	1(2)	10(1)	3(2)
N(1A)	40(2)	71(3)	64(2)	-1(2)	19(2)	4(2)
N(4)	44(2)	53(2)	48(2)	0(2)	15(1)	-3(2)
C(2)	60(2)	64(3)	67(3)	2(3)	28(2)	-16(3)
C(2A)	53(2)	58(3)	56(3)	5(2)	19(2)	6(2)
C(3)	45(2)	54(3)	55(2)	10(2)	18(2)	-5(2)
C(3A)	45(2)	43(3)	60(2)	1(2)	20(2)	-2(2)
C(3B)	49(2)	41(2)	51(2)	-3(2)	12(2)	-4(2)
C(4A)	51(2)	62(3)	70(3)	-4(3)	4(2)	3(3)
C(5)	53(2)	50(3)	64(3)	3(2)	18(2)	4(2)
C(5A)	95(4)	80(4)	64(3)	8(3)	3(3)	7(3)
C(6)	54(2)	75(3)	80(3)	11(3)	30(2)	14(3)
C(6A)	117(4)	76(4)	54(3)	4(3)	28(3)	-4(4)
C(7)	73(3)	56(3)	75(3)	-1(3)	34(3)	7(3)
C(7A)	55(2)	48(3)	58(2)	0(2)	22(2)	-7(2)
C(7B)	74(3)	58(3)	59(3)	-8(3)	30(2)	-5(3)
C(8A)	49(2)	38(3)	55(2)	-4(2)	16(2)	-3(2)
C(30)	50(2)	54(3)	65(3)	5(2)	22(2)	-4(2)
C(70)	108(4)	122(5)	84(3)	-12(4)	58(3)	18(4)
C(71)	54(2)	58(3)	56(3)	-1(3)	28(2)	-4(3)

C(72)	85(3)	69(4)	55(3)	-3(3)	14(2)	-8(3)
C(73)	78(3)	86(5)	62(3)	14(3)	13(3)	4(4)
C(74)	64(3)	75(4)	80(3)	29(3)	32(3)	10(3)
C(75)	69(3)	53(3)	101(4)	11(3)	28(3)	-8(3)
C(76)	56(3)	66(4)	70(3)	3(3)	19(2)	-10(3)

Table S10. Hydrogen bonds for **7j’** [Å and °].

D-H...A	d(D-H)	d(H...A)	d(D...A)	<(DHA)
N(1A)-H(1A)...O(5)#1	0.86	2.03	2.879(4)	171.0
C(2)-H(2B)...N(1A)#2	0.97	2.67	3.576(5)	155.4

Symmetry transformations used to generate equivalent atoms:

#1 x+1,y,z #2 -x+2,y+1/2,-z+1

X-ray crystallographic data for compound 8b

Table S11. Crystal data and structure refinement for compound **8b**

Identification code	Jb109	
Empirical formula	$C_{21}H_{19}FN_2O_2$	
Formula weight	350.38	
Temperature	294(2) K	
Wavelength	0.71073 Å	
Crystal system	Orthorhombic	
Space group	P 21 21 21	
Unit cell dimensions	a = 8.853(2) Å	α = 90°.
	b = 10.6234(14) Å	β = 90°.
	c = 18.662(3) Å	γ = 90°.
Volume	1755.1(5) $Å^3$	
Z	4	
Density (calculated)	1.326 Mg/m^3	
Absorption coefficient	0.093 mm^{-1}	
F(000)	736	
Crystal size	0.39 x 0.30 x 0.21 mm^3	
Theta range for data collection	2.18 to 24.97°.	
Index ranges	0<=h<=10, 0<=k<=12, 0<=l<=22	
Reflections collected	1852	
Independent reflections	1789	

Completeness to theta = 25.0°	99.9 %
Refinement method	Full-matrix least-squares on F^2
Data / restraints / parameters	1789 / 0 / 236
Goodness-of-fit on F^2	1.104
Final R indices [I>2sigma(I)]	R1 = 0.0385, wR2 = 0.0924
R indices (all data)	R1 = 0.0601, wR2 = 0.1013
Extinction coefficient	0.072(4)
Largest diff. peak and hole	0.129 and -0.117 e.$Å^{-3}$

Table S12. Atomic coordinates (x 10^4) and equivalent isotropic displacement parameters ($Å^2$x 10^3) for compound **8b.** U(eq) is defined as one third of the trace of the orthogonalized U^{ij} tensor.

	x	y	z	U(eq)
F(1)	3539(3)	-3942(2)	3814(1)	106(1)
O(1)	4905(3)	1008(3)	5459(1)	77(1)
O(5)	1077(3)	1137(2)	6898(1)	68(1)
N(4)	2901(2)	390(2)	6140(1)	46(1)
N(17)	1700(3)	-2856(2)	7593(1)	57(1)
C(2)	5334(4)	1100(3)	6187(2)	59(1)
C(3)	4216(3)	285(3)	6607(2)	49(1)
C(5)	1583(4)	983(3)	6294(2)	55(1)
C(6)	907(4)	1408(3)	5591(2)	74(1)
C(7)	2286(5)	1545(3)	5111(2)	75(1)
C(7A)	3384(3)	564(3)	5398(2)	52(1)
C(8)	3382(3)	-652(3)	4971(2)	53(1)
C(9)	4176(5)	-717(4)	4331(2)	80(1)
C(10)	4197(5)	-1821(5)	3933(2)	91(1)
C(11)	3467(4)	-2845(4)	4192(2)	73(1)
C(12)	2662(4)	-2828(3)	4808(2)	66(1)
C(13)	2635(4)	-1712(3)	5198(2)	58(1)
C(14)	4769(3)	-1054(3)	6702(2)	52(1)

C(15)	3634(3)	-1968(3)	7003(1)	47(1)
C(16)	2460(4)	-1750(3)	7453(2)	53(1)
C(18)	2360(3)	-3797(3)	7216(2)	50(1)
C(19)	1962(4)	-5068(3)	7152(2)	63(1)
C(20)	2831(5)	-5804(3)	6711(2)	77(1)
C(21)	4058(5)	-5314(3)	6340(2)	80(1)
C(22)	4464(4)	-4066(3)	6403(2)	64(1)
C(23)	3601(3)	-3285(3)	6843(1)	48(1)

Table S13. Bond lengths [Å] and angles [°] for compound **8b**.

F(1)-C(11)	1.363(4)
O(1)-C(2)	1.414(4)
O(1)-C(7A)	1.431(4)
O(5)-C(5)	1.223(4)
N(4)-C(5)	1.357(4)
N(4)-C(3)	1.458(3)
N(4)-C(7A)	1.460(4)
N(17)-C(18)	1.355(4)
N(17)-C(16)	1.379(4)
C(2)-C(3)	1.531(4)
C(3)-C(14)	1.515(4)
C(5)-C(6)	1.512(4)
C(6)-C(7)	1.521(5)
C(7)-C(7A)	1.522(5)
C(7A)-C(8)	1.518(4)
C(8)-C(13)	1.373(4)
C(8)-C(9)	1.388(4)
C(9)-C(10)	1.388(5)
C(10)-C(11)	1.354(5)
C(11)-C(12)	1.352(5)
C(12)-C(13)	1.392(4)

C(14)-C(15)	1.505(4)
C(15)-C(16)	1.357(4)
C(15)-C(23)	1.431(4)
C(18)-C(19)	1.400(4)
C(18)-C(23)	1.409(4)
C(19)-C(20)	1.371(5)
C(20)-C(21)	1.389(5)
C(21)-C(22)	1.379(5)
C(22)-C(23)	1.396(4)
C(2)-O(1)-C(7A)	110.6(2)
C(5)-N(4)-C(3)	126.5(2)
C(5)-N(4)-C(7A)	113.2(2)
C(3)-N(4)-C(7A)	110.0(2)
C(18)-N(17)-C(16)	108.7(2)
O(1)-C(2)-C(3)	106.2(2)
N(4)-C(3)-C(14)	113.6(2)
N(4)-C(3)-C(2)	99.6(2)
C(14)-C(3)-C(2)	112.5(2)
O(5)-C(5)-N(4)	124.9(3)
O(5)-C(5)-C(6)	128.0(3)
N(4)-C(5)-C(6)	107.1(3)
C(5)-C(6)-C(7)	102.8(3)
C(6)-C(7)-C(7A)	103.9(2)

O(1)-C(7A)-N(4)	104.0(2)
O(1)-C(7A)-C(8)	108.9(3)
N(4)-C(7A)-C(8)	112.9(2)
O(1)-C(7A)-C(7)	113.8(3)
N(4)-C(7A)-C(7)	103.5(3)
C(8)-C(7A)-C(7)	113.4(3)
C(13)-C(8)-C(9)	118.0(3)
C(13)-C(8)-C(7A)	122.5(2)
C(9)-C(8)-C(7A)	119.6(3)
C(8)-C(9)-C(10)	120.6(4)
C(11)-C(10)-C(9)	118.8(3)
C(12)-C(11)-C(10)	122.9(4)
C(12)-C(11)-F(1)	118.4(4)
C(10)-C(11)-F(1)	118.6(3)
C(11)-C(12)-C(13)	117.7(4)
C(8)-C(13)-C(12)	121.9(3)
C(15)-C(14)-C(3)	115.7(2)
C(16)-C(15)-C(23)	106.2(3)
C(16)-C(15)-C(14)	129.2(3)
C(23)-C(15)-C(14)	124.5(3)
C(15)-C(16)-N(17)	110.2(3)
N(17)-C(18)-C(19)	130.3(3)
N(17)-C(18)-C(23)	107.9(3)

C(19)-C(18)-C(23)	121.7(3)
C(20)-C(19)-C(18)	117.4(3)
C(19)-C(20)-C(21)	121.6(3)
C(22)-C(21)-C(20)	121.5(4)
C(21)-C(22)-C(23)	118.6(3)
C(22)-C(23)-C(18)	119.2(3)
C(22)-C(23)-C(15)	133.8(3)
C(18)-C(23)-C(15)	106.9(3)

Table S14. Anisotropic displacement parameters ($Å^2$x 10^3) for compound **8b**. The anisotropic displacement factor exponent takes the form: $-2\pi^2[h^2a^{*2}U^{11} + ... + 2 h k a^* b^* U^{12}]$

	U^{11}	U^{22}	U^{33}	U^{23}	U^{13}	U^{12}
F(1)	93(2)	116(2)	109(2)	-55(2)	-4(2)	16(2)
O(1)	65(2)	101(2)	65(1)	8(1)	2(1)	-38(2)
O(5)	62(1)	65(1)	76(1)	-5(1)	18(1)	10(1)
N(4)	44(1)	46(1)	48(1)	2(1)	2(1)	-1(1)
N(17)	49(1)	64(2)	57(1)	7(1)	12(1)	-7(1)
C(2)	56(2)	50(2)	71(2)	2(2)	-4(2)	-13(2)
C(3)	43(2)	55(2)	48(2)	0(2)	-3(1)	-5(2)
C(5)	48(2)	43(2)	74(2)	4(2)	0(2)	-3(2)
C(6)	62(2)	66(2)	93(3)	16(2)	-16(2)	8(2)
C(7)	96(3)	68(2)	62(2)	18(2)	-8(2)	0(2)
C(7A)	48(2)	58(2)	51(2)	11(2)	0(2)	-8(2)
C(8)	42(2)	71(2)	46(2)	6(2)	3(1)	-2(2)
C(9)	81(3)	94(3)	67(2)	0(2)	20(2)	-16(2)
C(10)	78(3)	123(4)	73(2)	-25(3)	26(2)	-11(3)
C(11)	60(2)	90(3)	68(2)	-31(2)	-9(2)	10(2)
C(12)	64(2)	71(2)	61(2)	-6(2)	-10(2)	-3(2)
C(13)	56(2)	70(2)	47(2)	-2(2)	-1(2)	-7(2)
C(14)	41(2)	56(2)	60(2)	7(2)	-3(1)	-2(2)

C(15)	41(2)	51(2)	48(2)	7(1)	-5(1)	-3(1)
C(16)	52(2)	51(2)	58(2)	1(2)	0(2)	2(2)
C(18)	50(2)	52(2)	46(2)	8(1)	-8(2)	-2(2)
C(19)	64(2)	61(2)	65(2)	16(2)	-10(2)	-10(2)
C(20)	103(3)	48(2)	81(2)	5(2)	-5(2)	-8(2)
C(21)	104(3)	54(2)	83(2)	5(2)	14(2)	13(2)
C(22)	69(2)	59(2)	65(2)	11(2)	11(2)	5(2)
C(23)	47(2)	52(2)	44(2)	8(1)	-2(1)	2(1)

Table S15. Hydrogen bonds for compound **8b** [Å and °].

D-H...A	d(D-H)	d(H...A)	d(D...A)	<(DHA)
N(17)-H(17)...O(5)#1	0.86	2.08	2.845(3)	147.8

Symmetry transformations used to generate equivalent atoms:

#1 -x,y-1/2,-z+3/2

Depletion plot of compound 7s

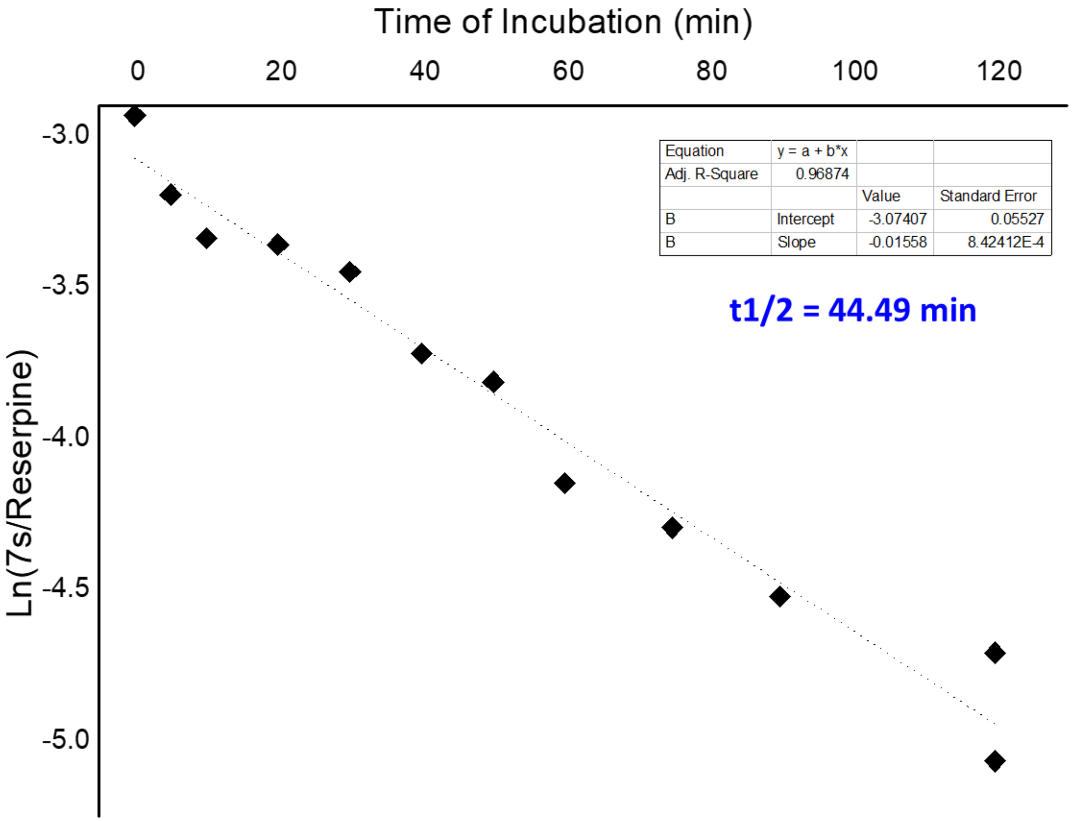

Figure S1. Depletion plot of compound **7s**.

<u>HRMS characterization of compound **7s**</u>: Compound **7s** displays its protonated molecule at *m/z* 477.1148 with the characteristic isotopic pattern of a di-chlorinated compound and elutes with retention time 17.9 min (**Figure S2.A**). In the MS/MS spectrum (**Figure S2.B**) is observed a fragment ion at *m/z* 304.0289 +/- 0.3 ppm and minor fragment ions at *m/z* 156.0809 +/- 0.6 ppm, *m/z* 174.0911 +/- 1.1 ppm and *m/z* 346.0398 +/- 0.6 ppm, which are in accordance with the proposed fragmentation mechanisms given in **Figure S2.C**.

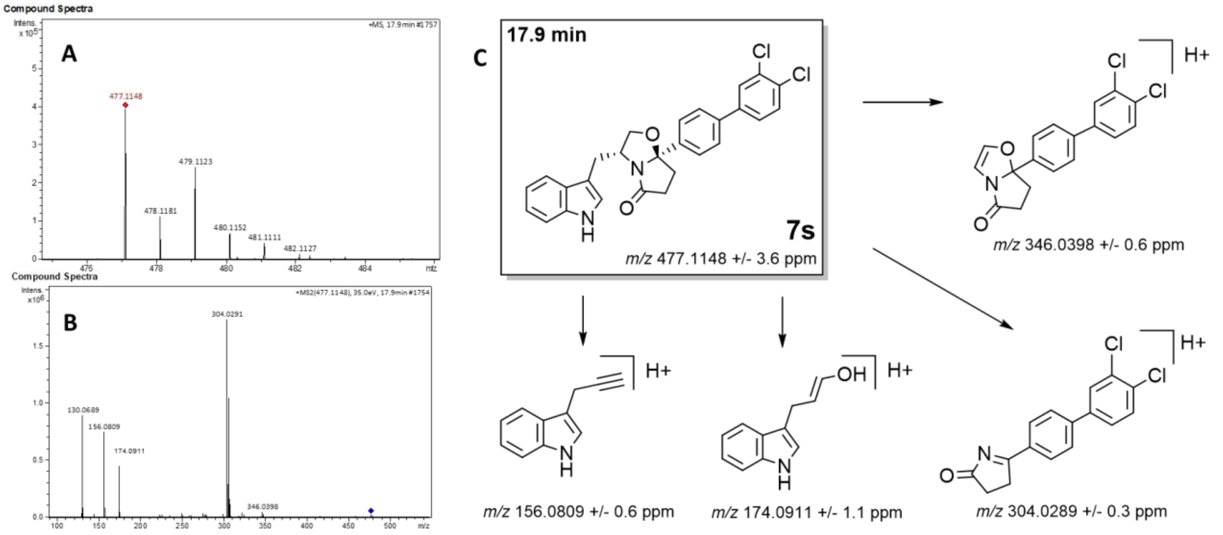

Figure S2. A) Full HRMS ESI(+) spectrum of compound **7s**; **B)** HRMS/MS spectrum; and **C)** proposed fragmentation mechanism of ion at *m/z* 477.1143 ± 3.6 ppm, corresponding to the protonated molecule of **7s**.

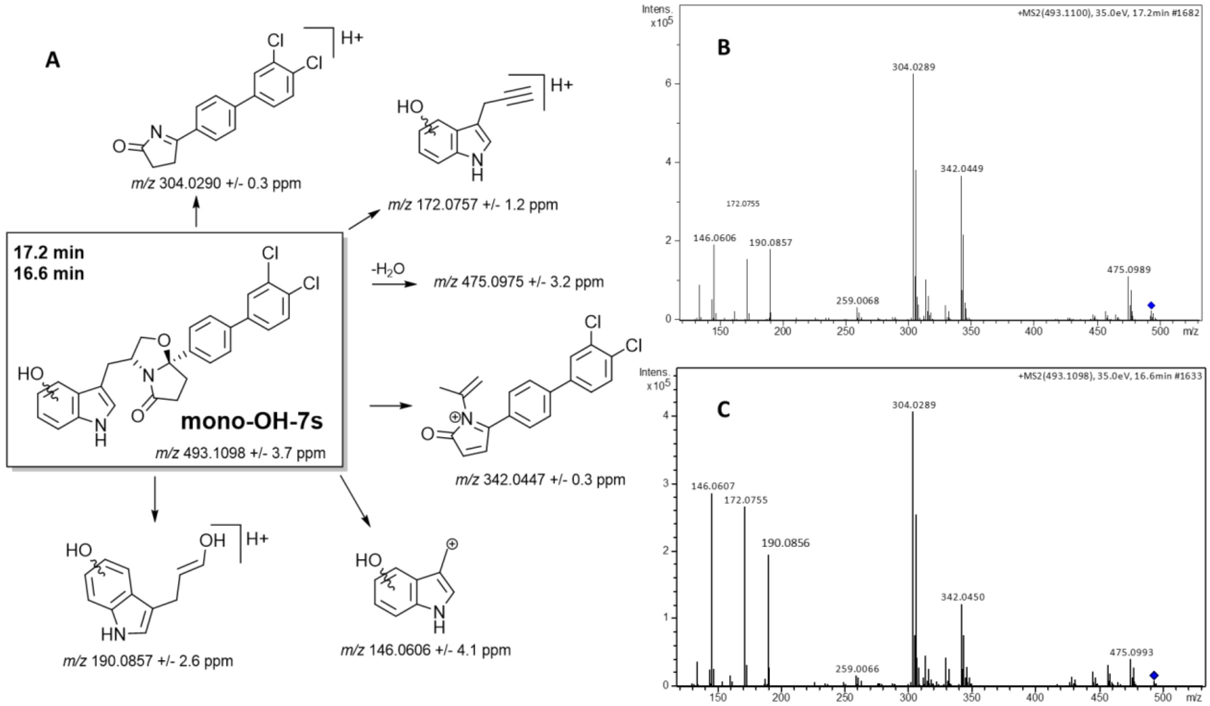

Figure S3. A) Proposed MS/MS-ESI(+) fragmentation mechanism; and **B)** and **C)** HRMS/MS-ESI(+) spectra of ions at *m/z* 493.1100 and *m/z* 493.1098, corresponding to the protonated molecules of two close eluting mono-hydroxylated metabolites, **mono-OH-7s**, identified upon *in vitro* incubation of compound **7s** in human liver microsomes.

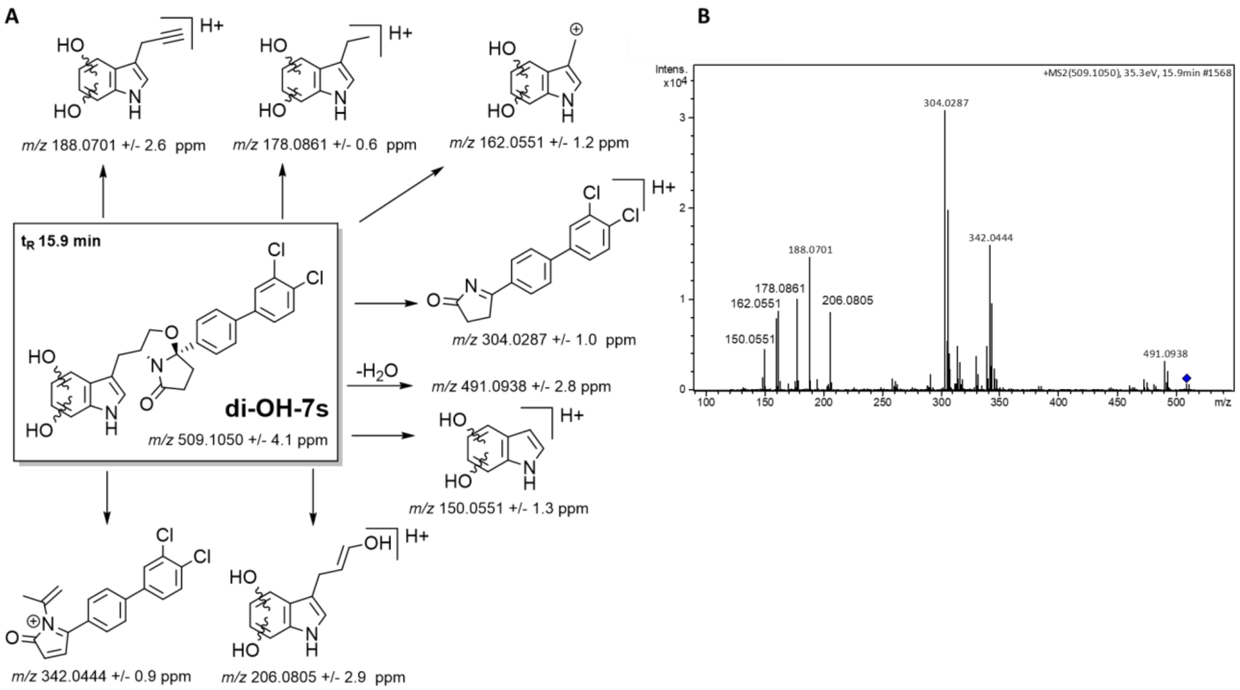

Figure S4. A) Proposed MS/MS-ESI(+) fragmentation mechanism; and **B)** HRMS/MS-ESI(+) spectrum of ion at *m/z* 509.1050 ± 4.1 ppm, corresponding to the di-hydroxylated metabolite identified in **7s** incubations in human liver microsomes.

NMR spectra

^{1}H NMR of compound **8a** ($CDCl_3$)

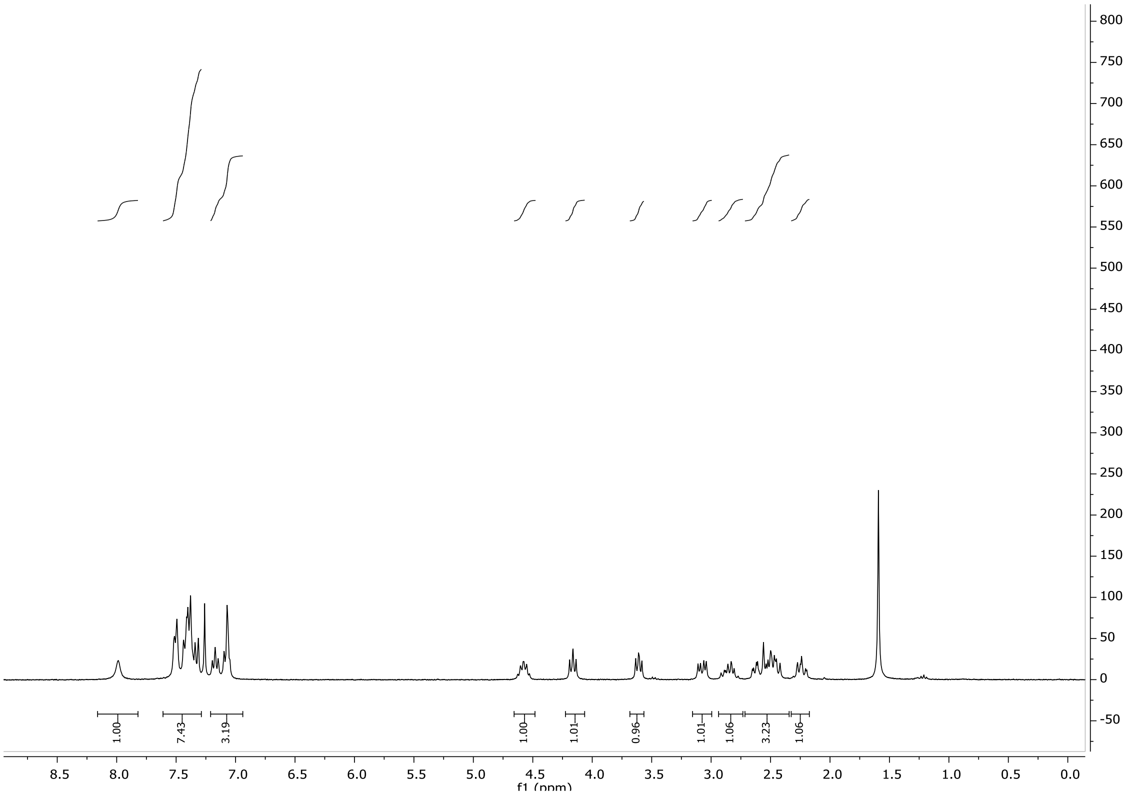

^{1}H NMR of compound **8b** ($CDCl_3$)

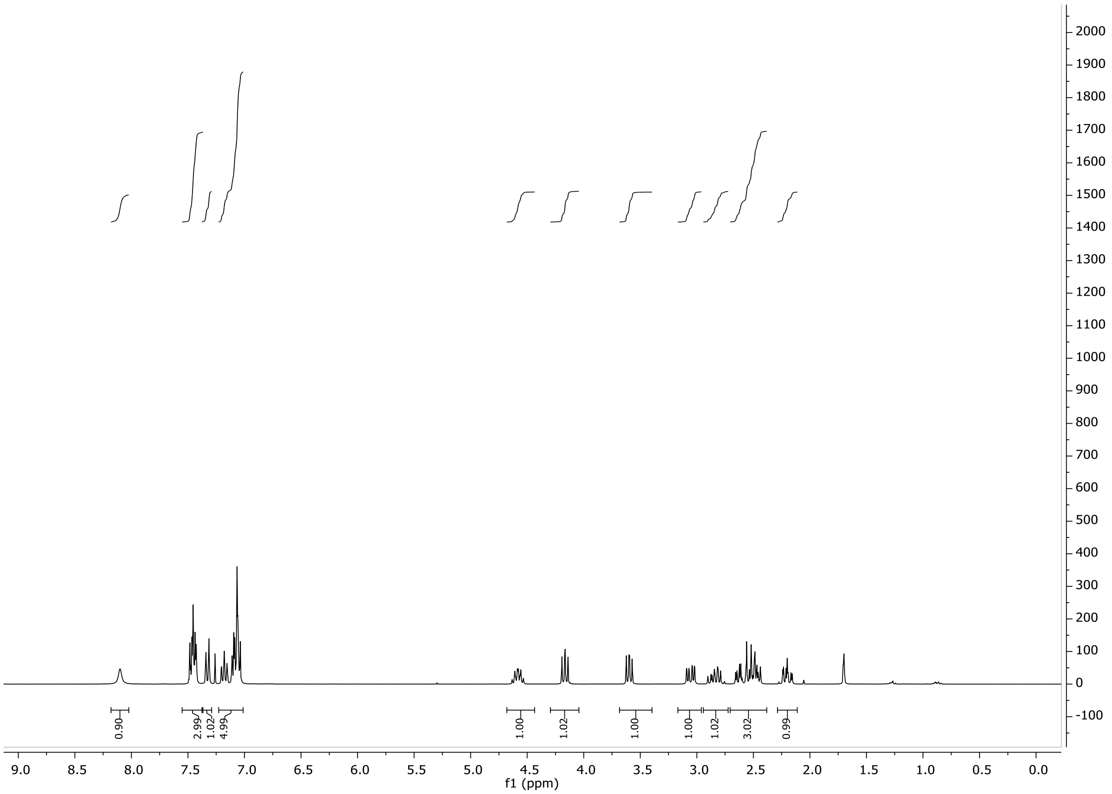

^{13}C NMR (APT) of compound **8b** ($CDCl_3$)

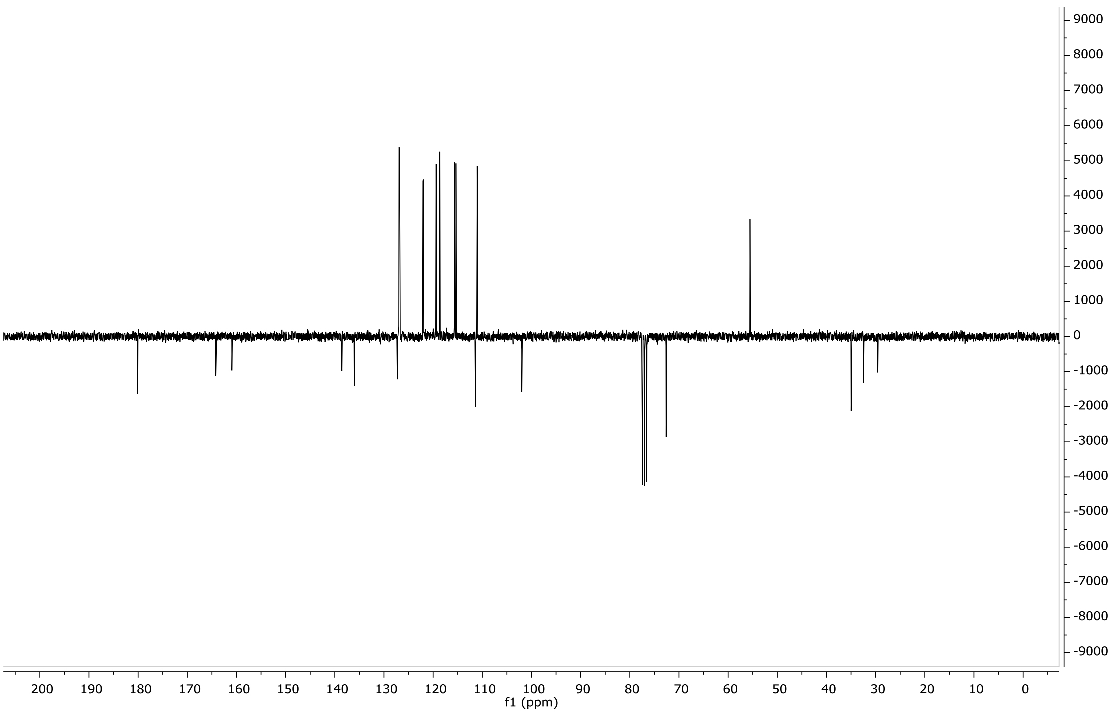

^{1}H NMR of compound **8c** ($CDCl_3$)

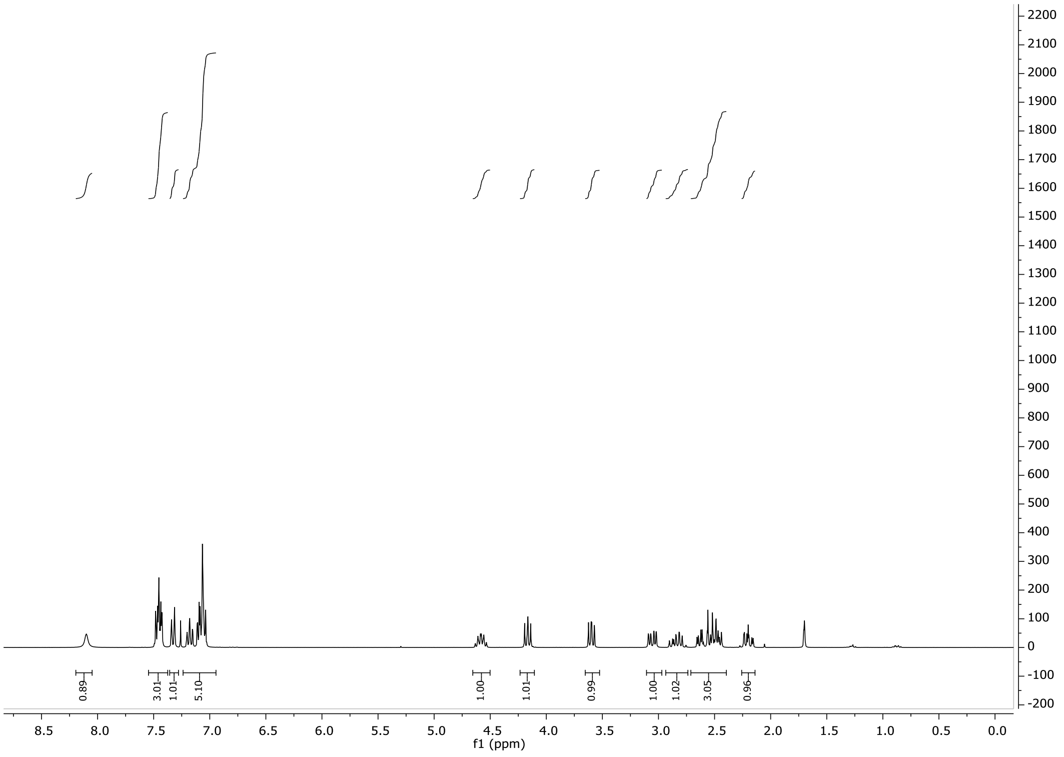

^{13}C NMR (APT) of compound **8c** ($CDCl_3$)

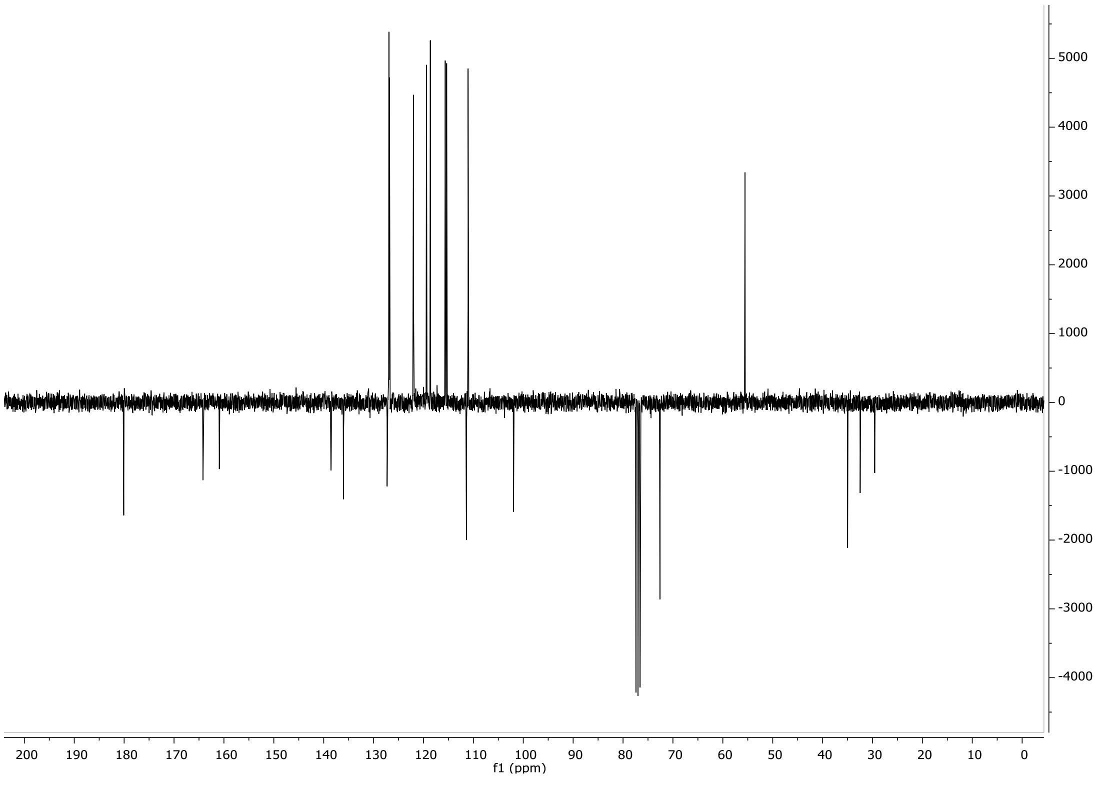

^{1}H NMR of compound **8d** ($CDCl_3$)

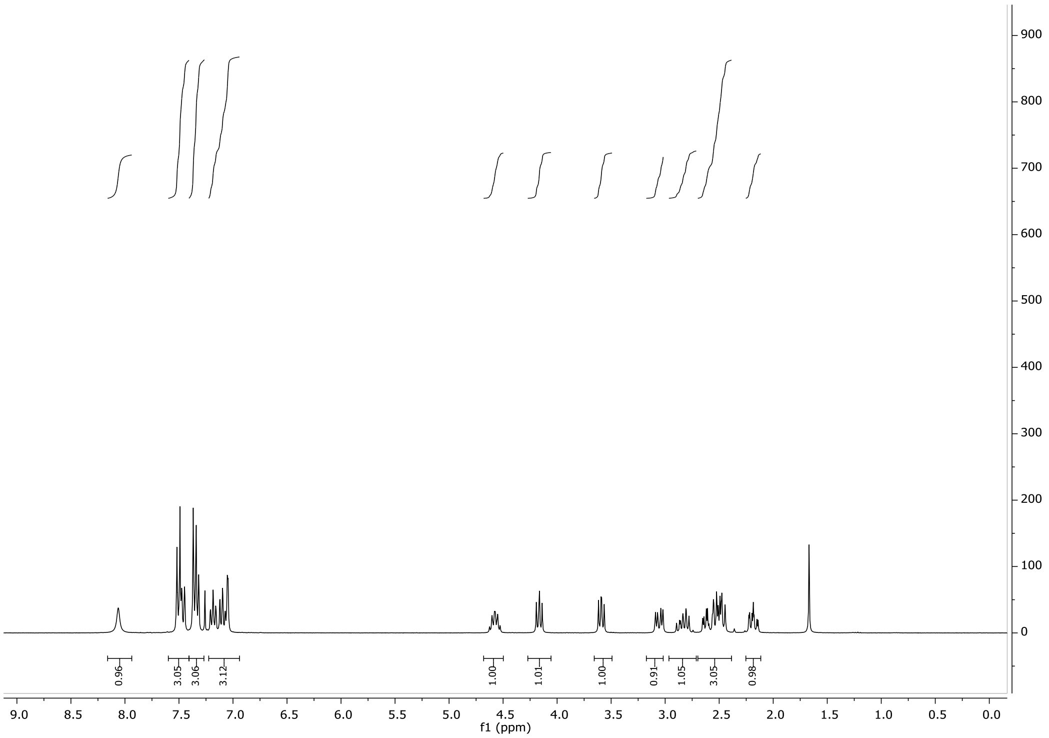

^{13}C NMR of compound **8d** ($CDCl_3$)

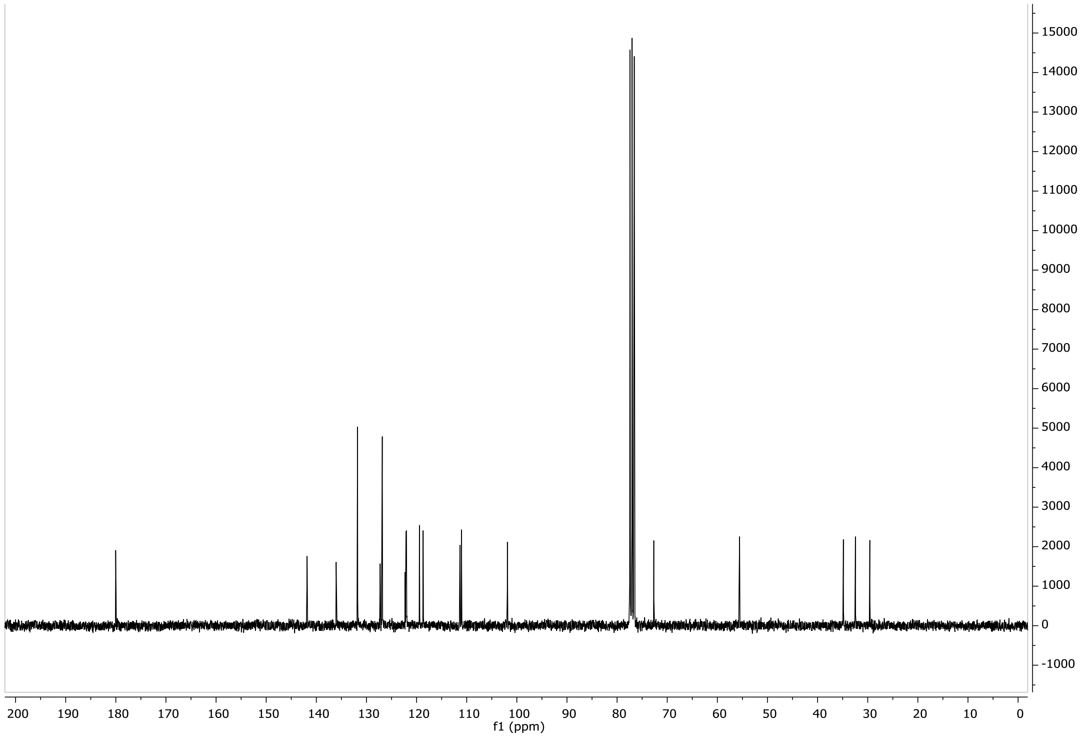

^{1}H NMR of compound **8e** ($CDCl_3$)

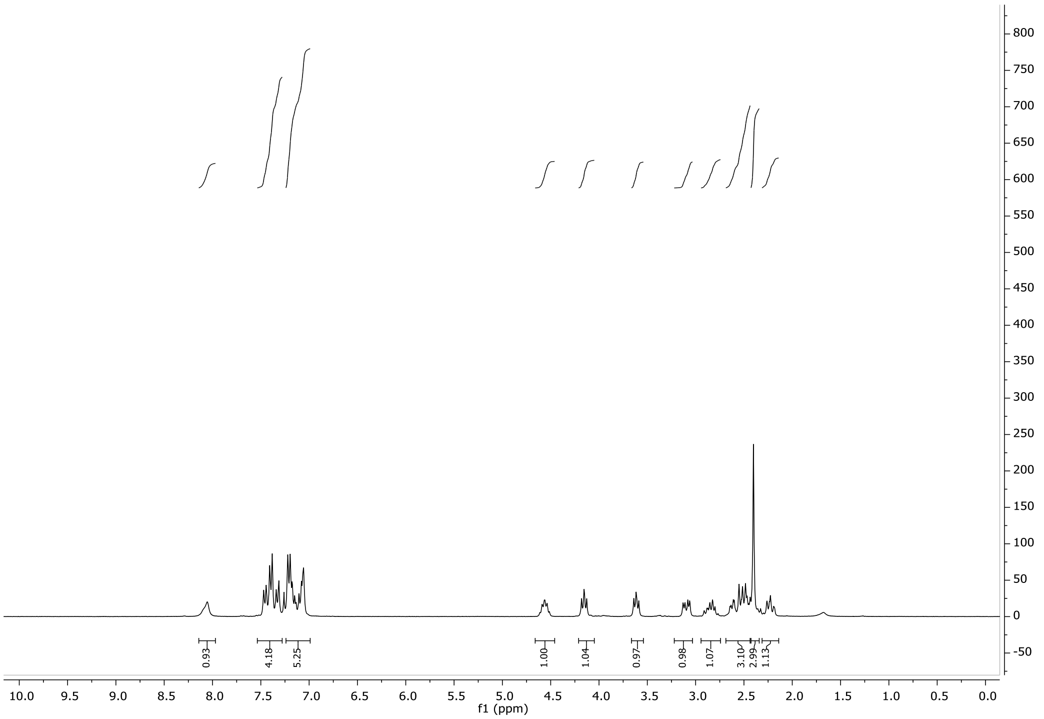

^{13}C NMR (APT) of compound **8e** ($CDCl_3$)

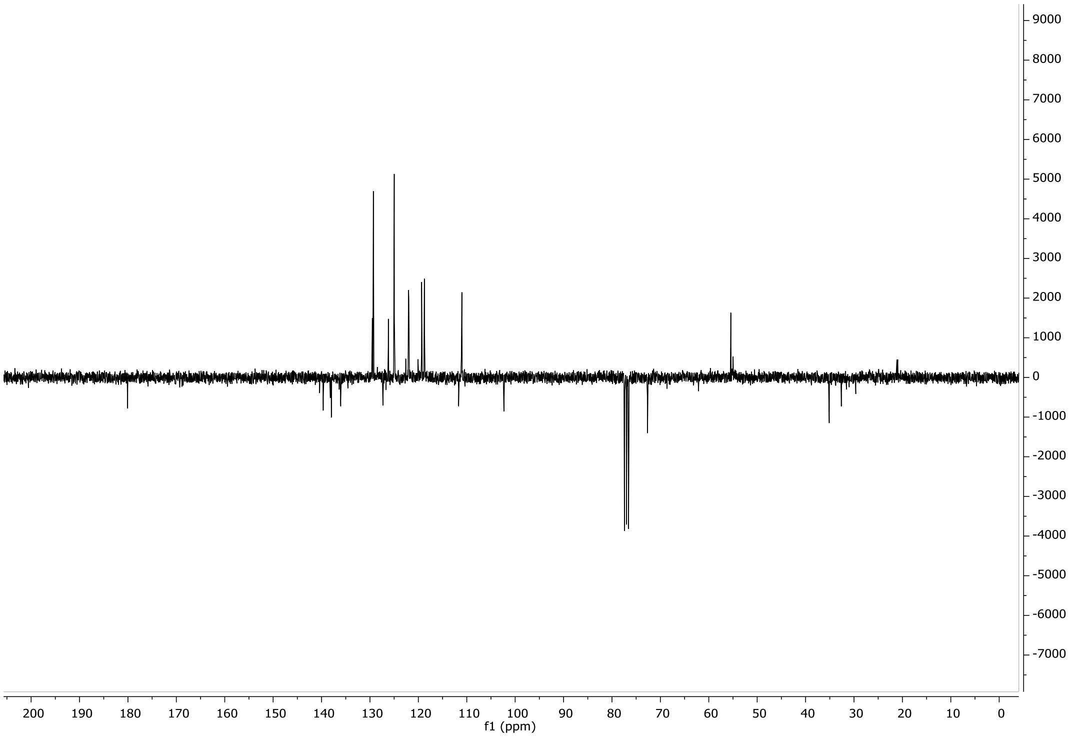

^{1}H NMR of compound **8f** ($CDCl_3$)

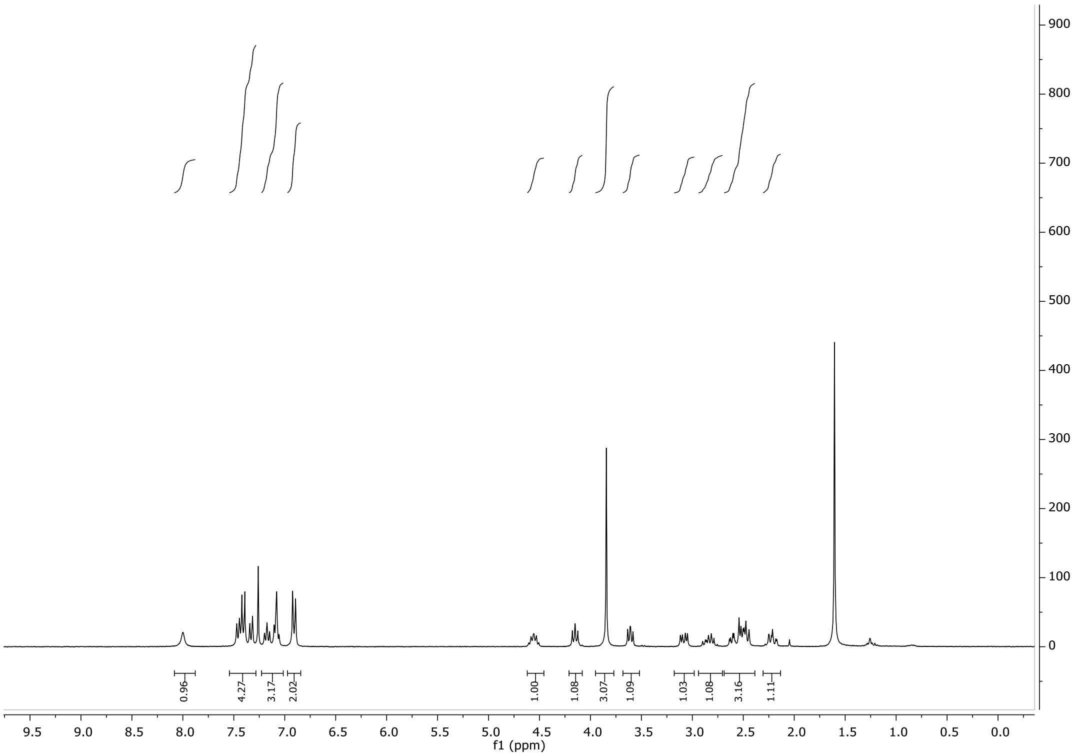

^{13}C NMR (APT) of compound **8f** ($CDCl_3$)

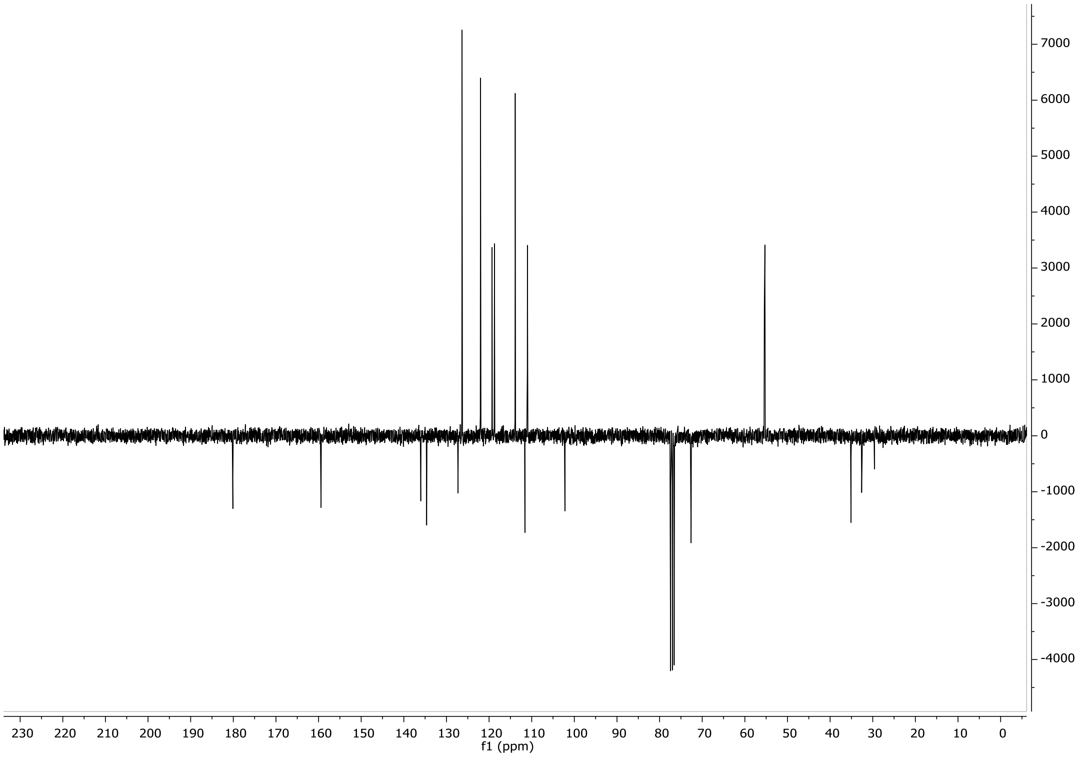

^{1}H NMR of compound **8g** ($CDCl_3$)

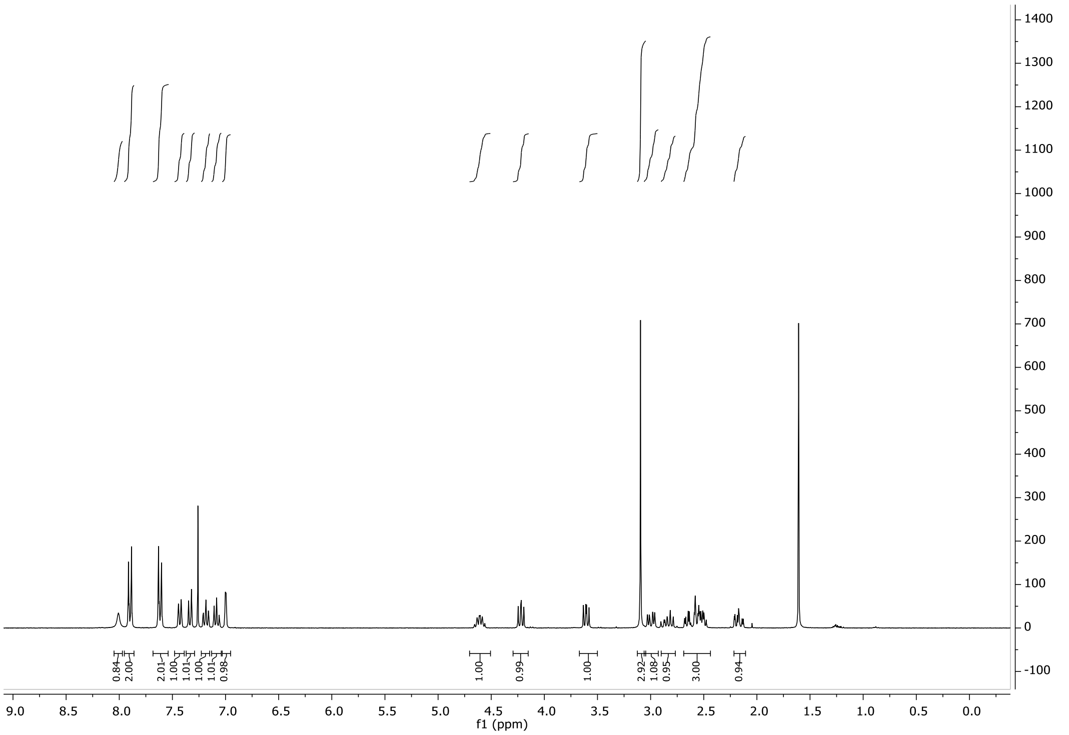

^{13}C NMR (APT) of compound **8g** ($CDCl_3$)

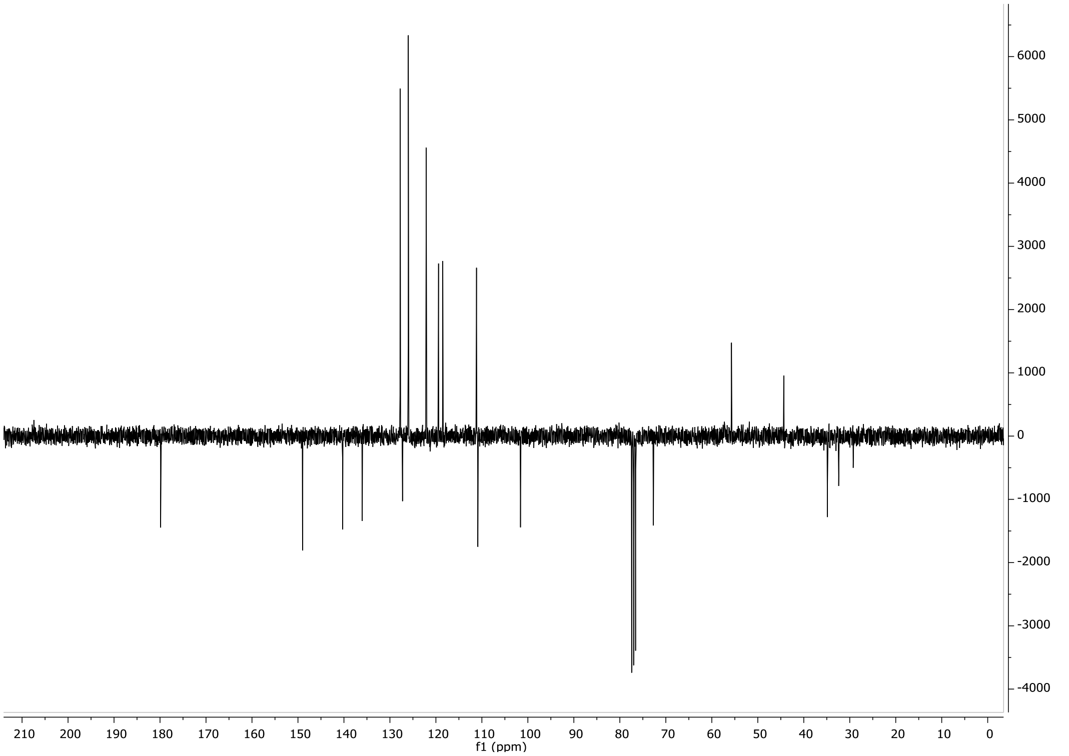

^{1}H NMR of compound **7h** ($CDCl_3$)

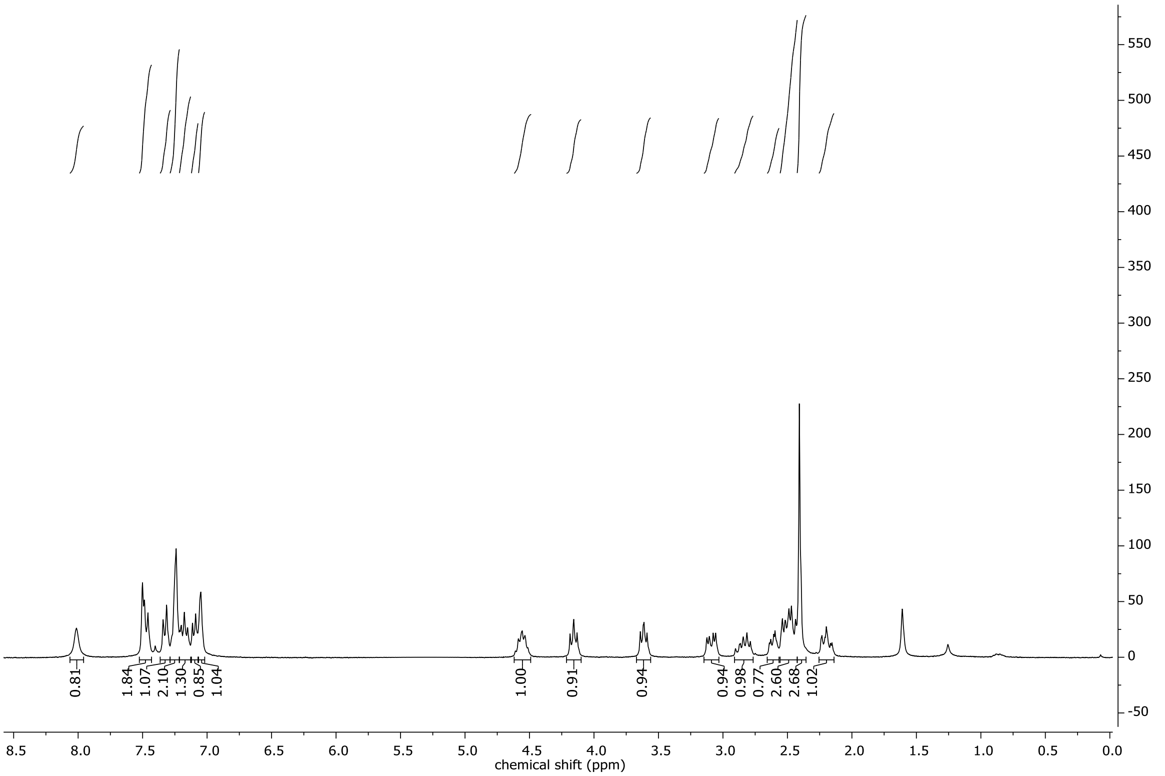

^{13}C NMR of compound **7h** ($CDCl_3$)

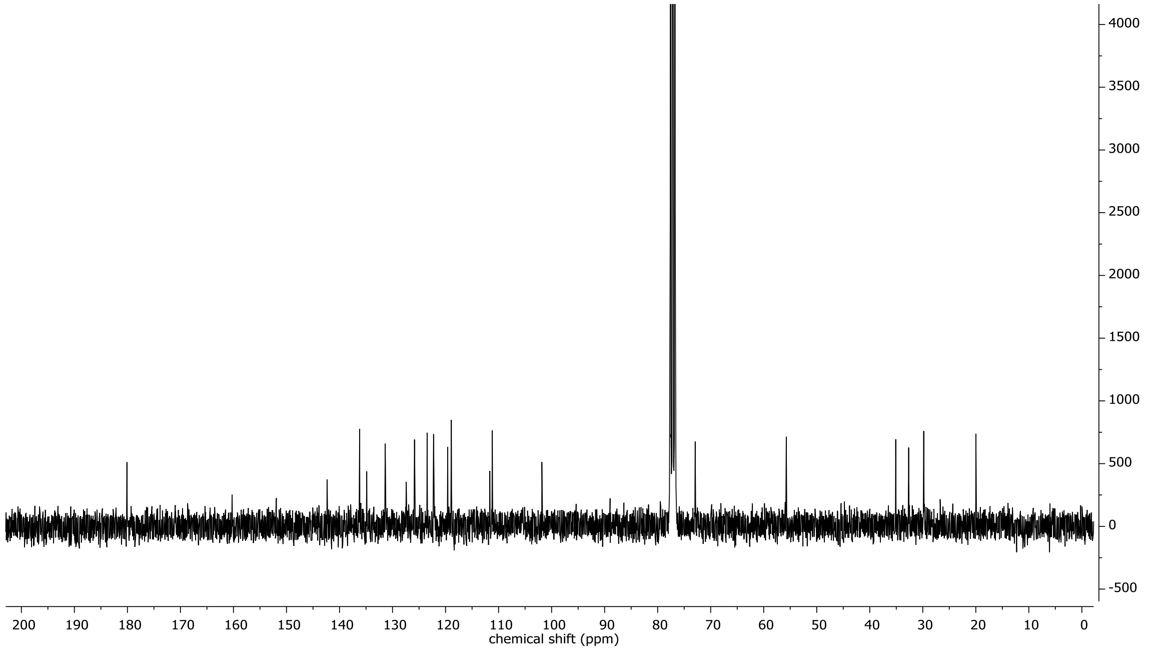

^{1}H NMR of compound **7j** ($CDCl_3$)

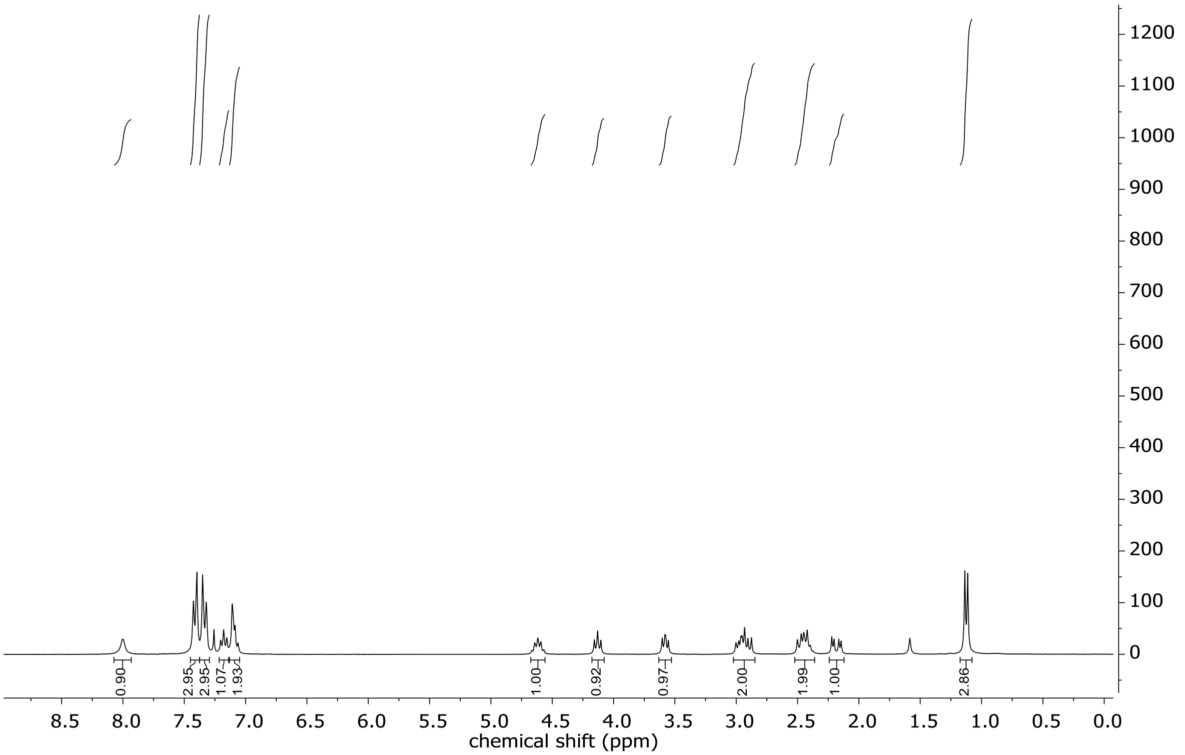

^{13}C NMR of compound **7j** ($CDCl_3$)

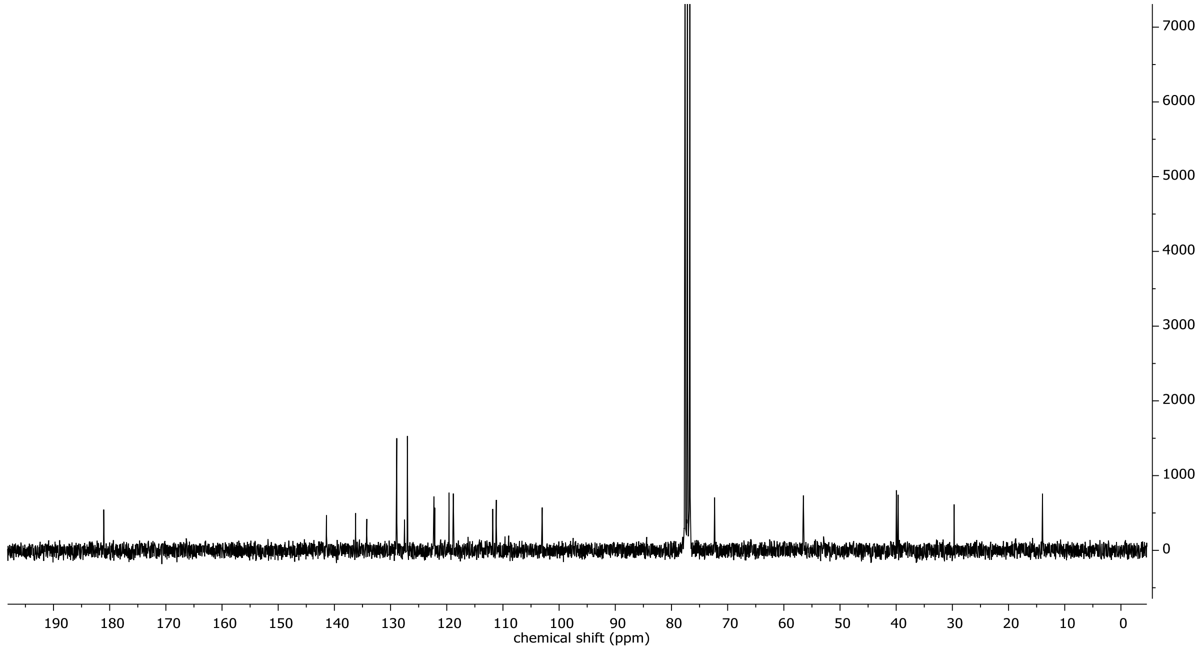

^{1}H NMR of compound **7j'** ($CDCl_3$)

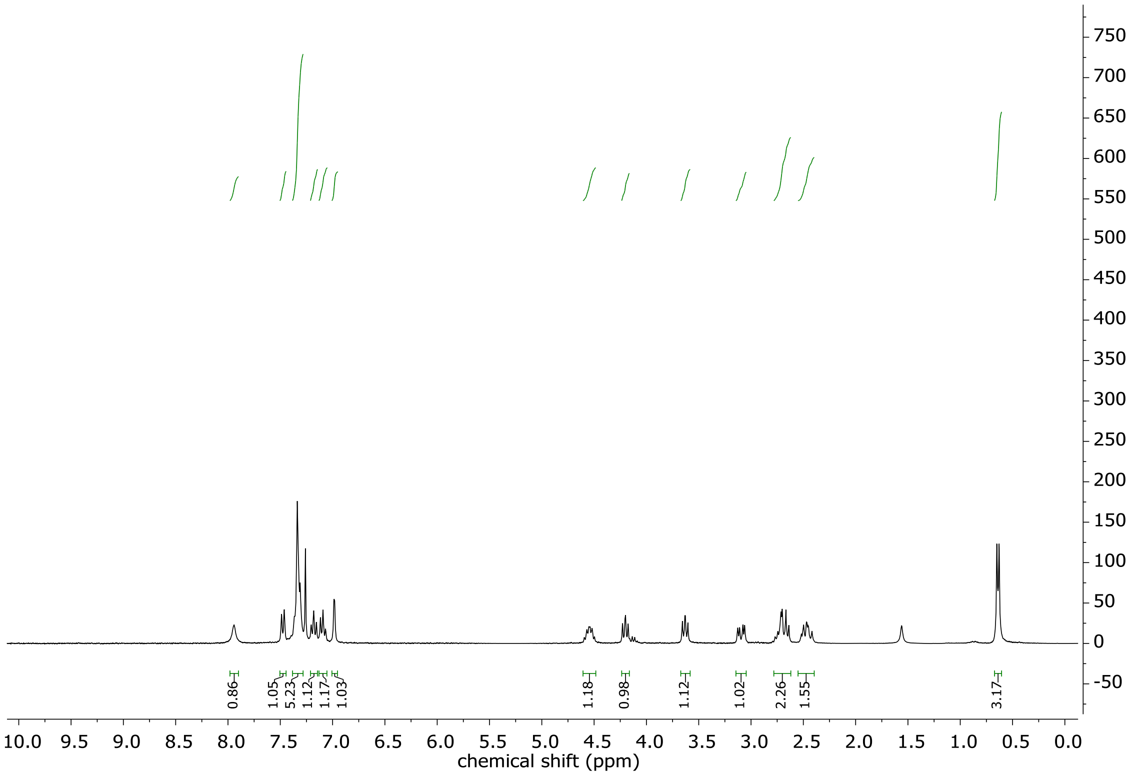

^{13}C NMR of compound **7j'** ($CDCl_3$)

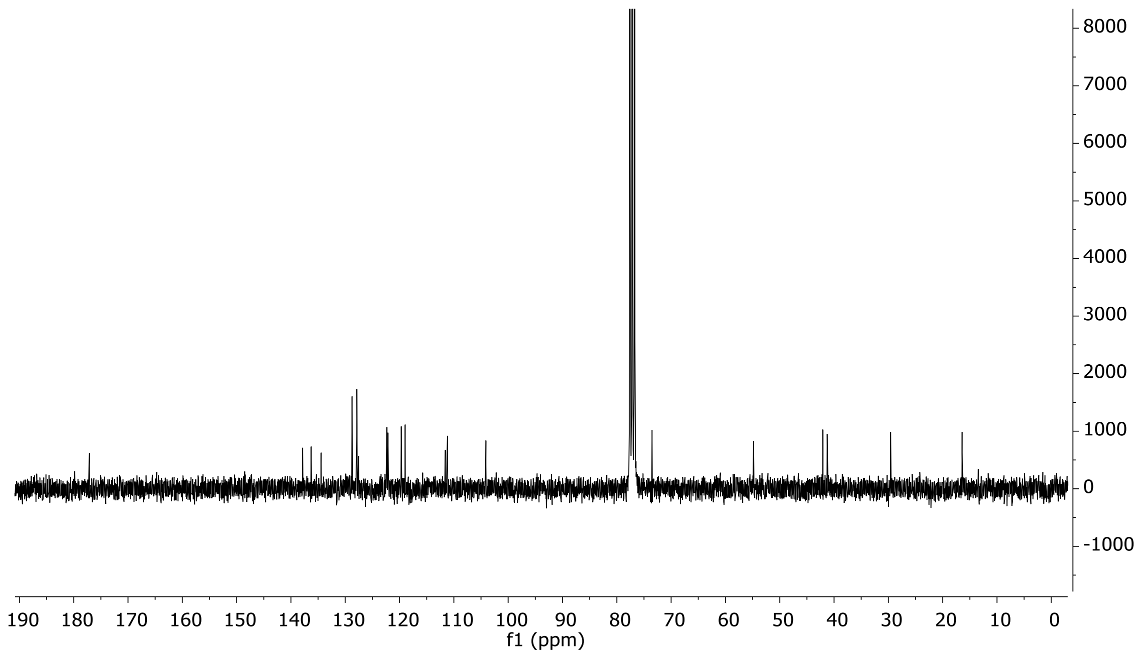

^{1}H NMR of compound **7k** ($CDCl_3$)

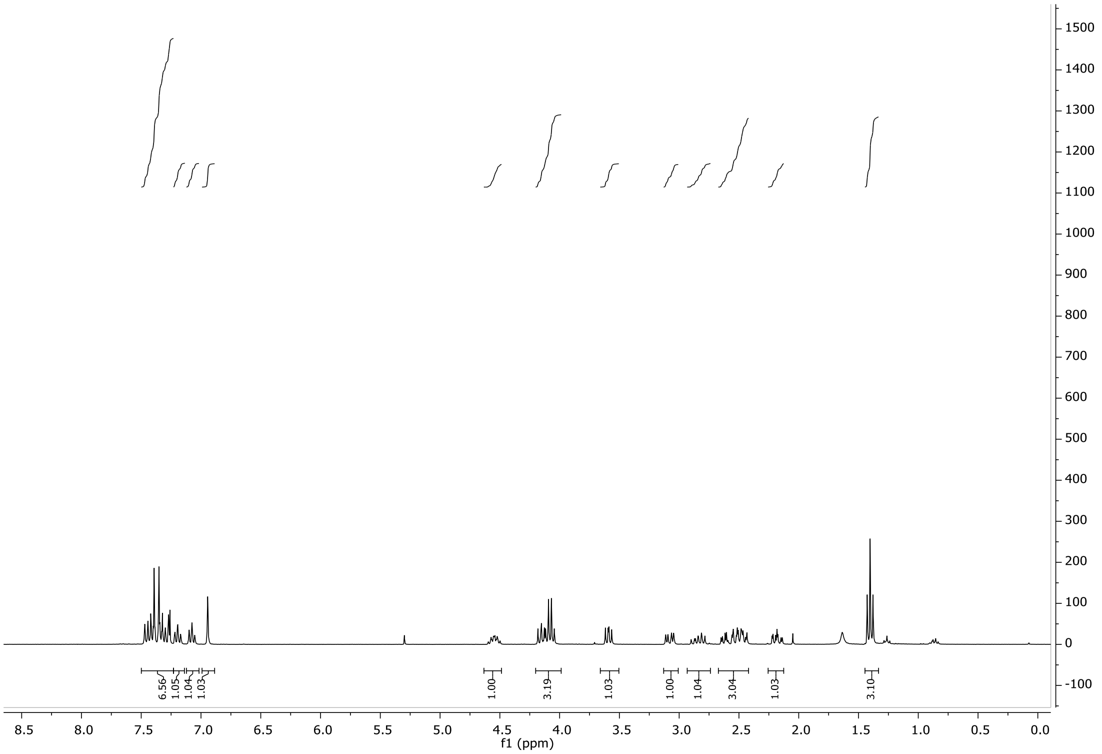

^{13}C NMR (APT) of compound **7k** ($CDCl_3$)

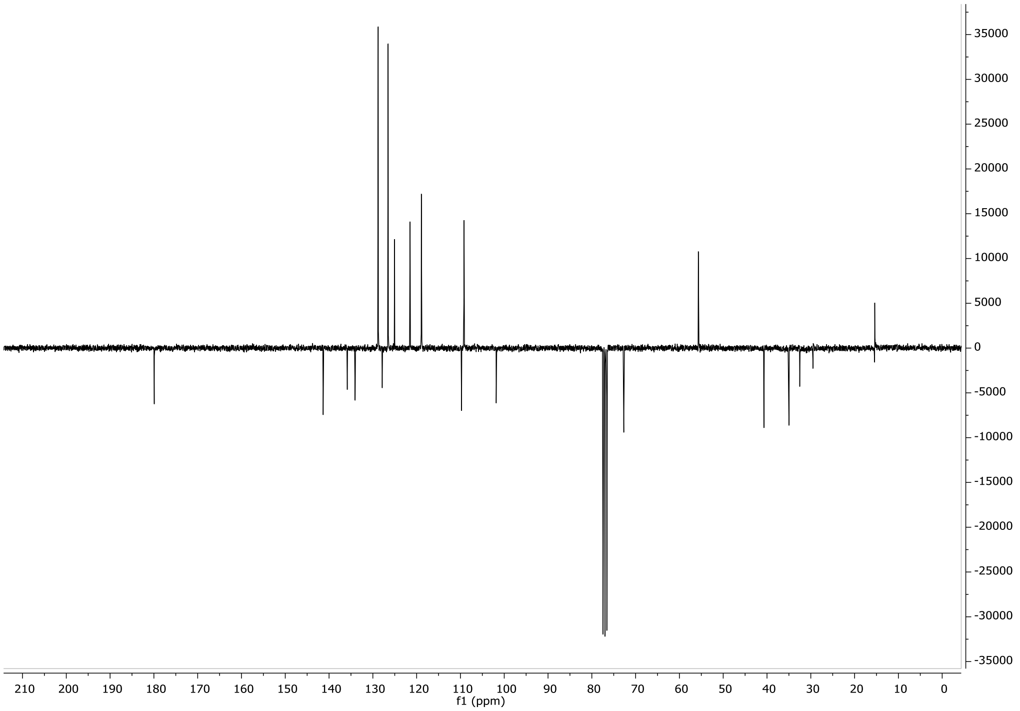

^{1}H NMR of compound **7l** ($CDCl_3$)

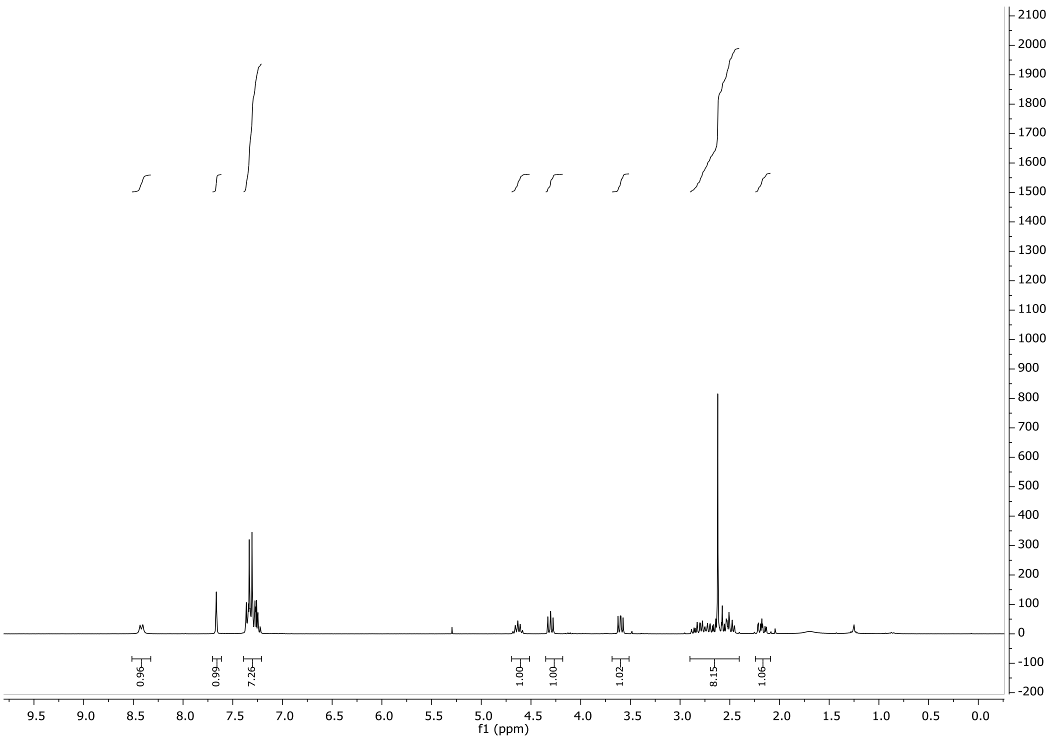

^{13}C NMR (APT) of compound **7l** ($CDCl_3$)

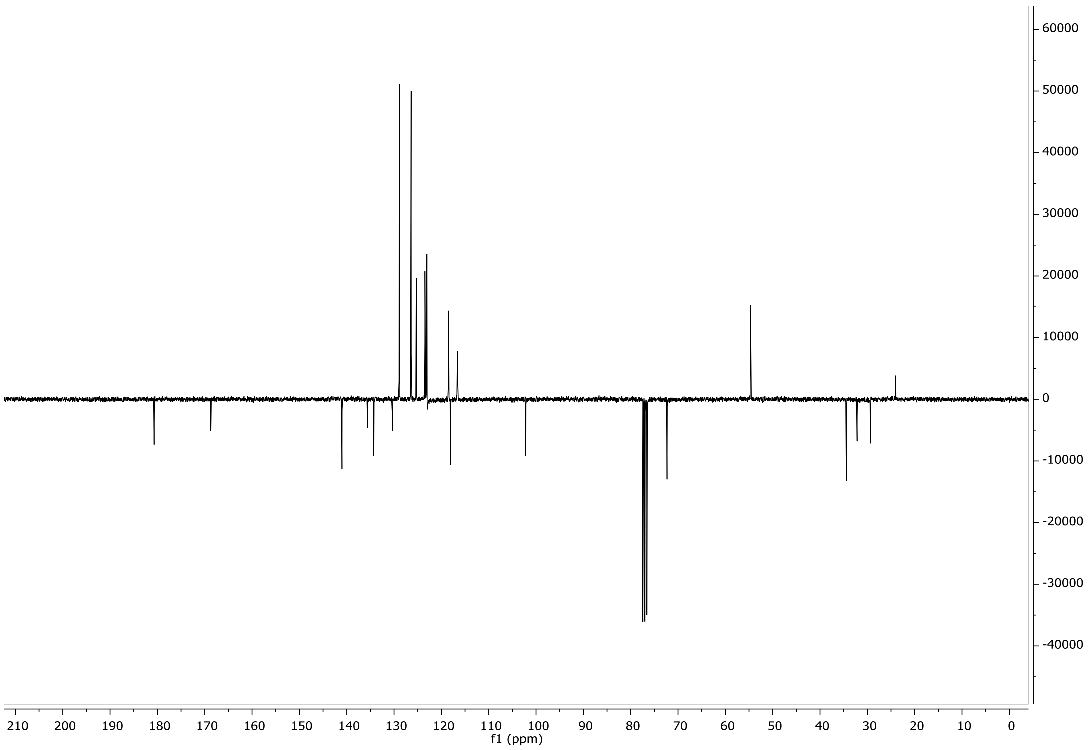

^{1}H NMR of compound **7m** ($CDCl_3$)

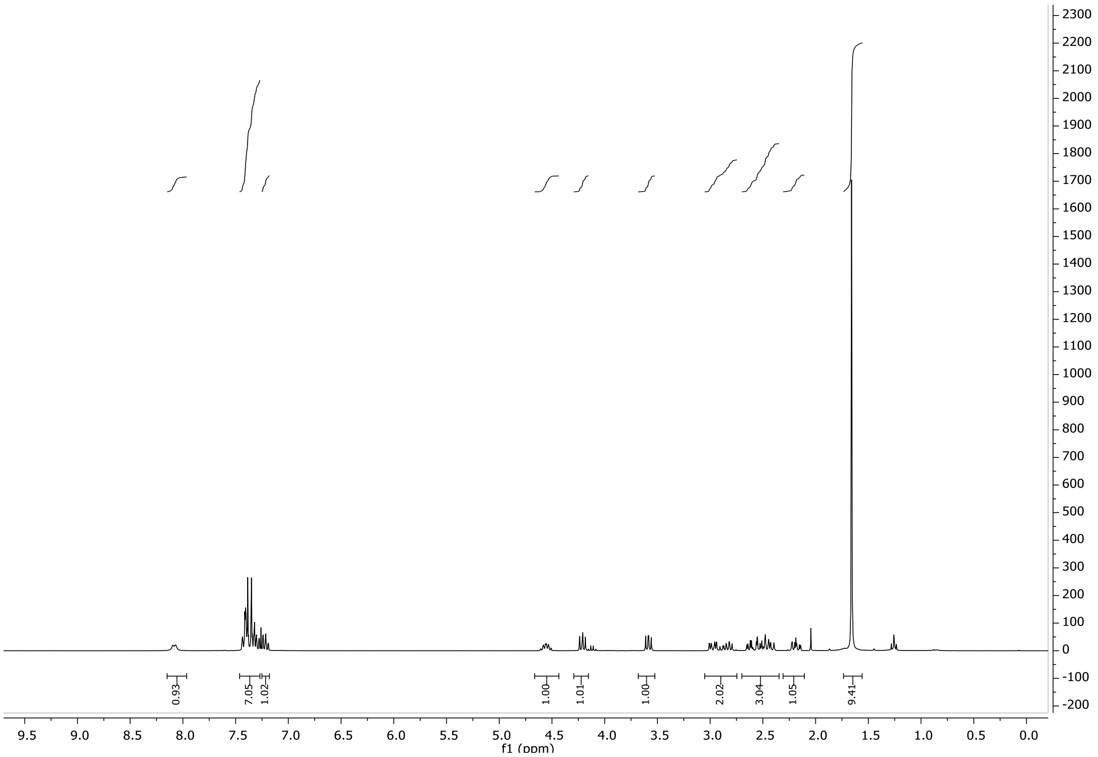

^{13}C NMR (APT) of compound **7m** ($CDCl_3$)

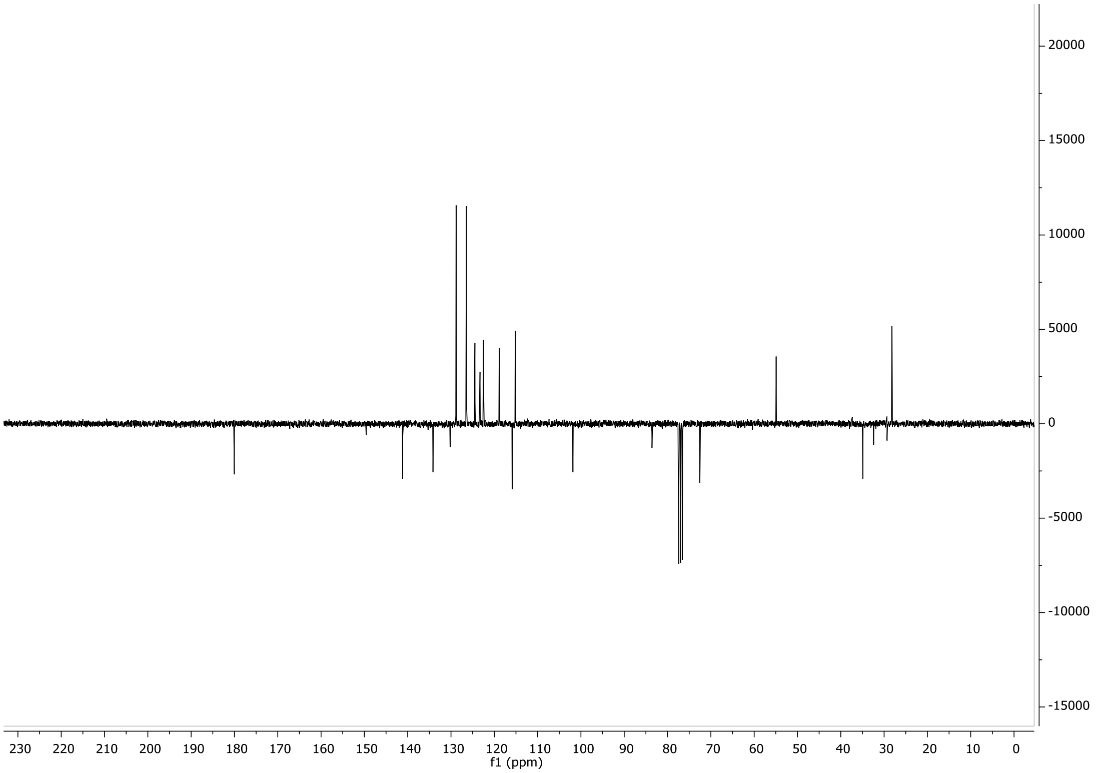

^{1}H NMR of compound **7n** ($CDCl_3$)

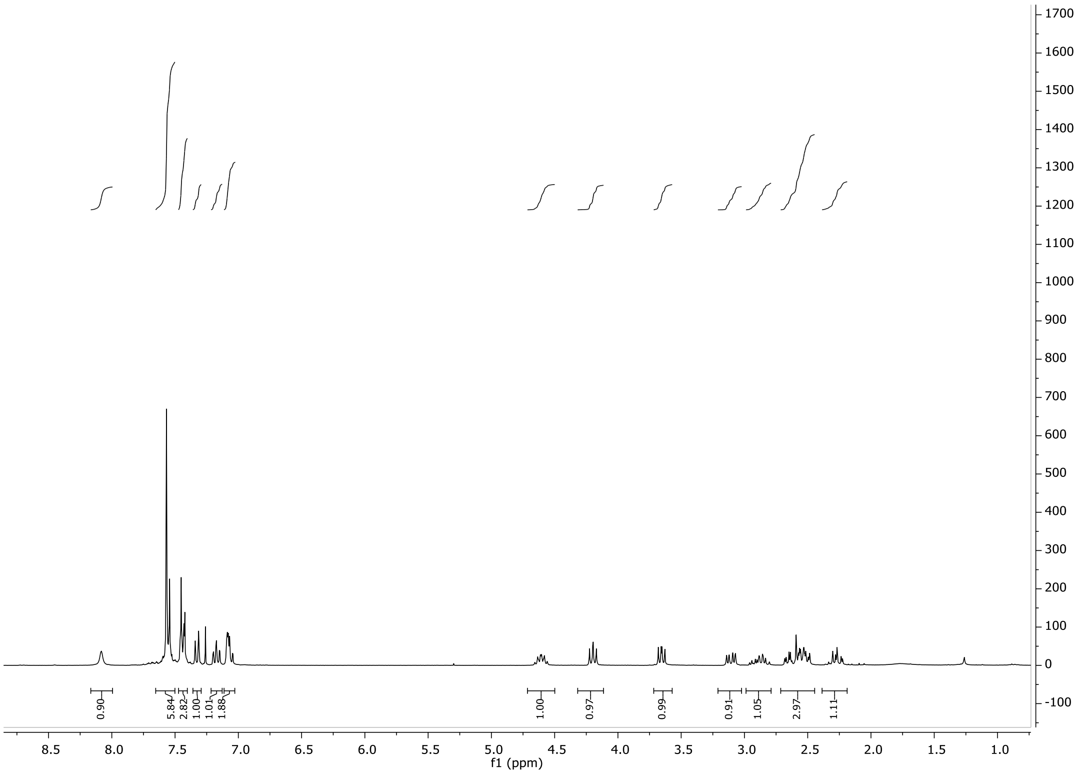

^{13}C NMR (APT) of compound **7n** ($CDCl_3$)

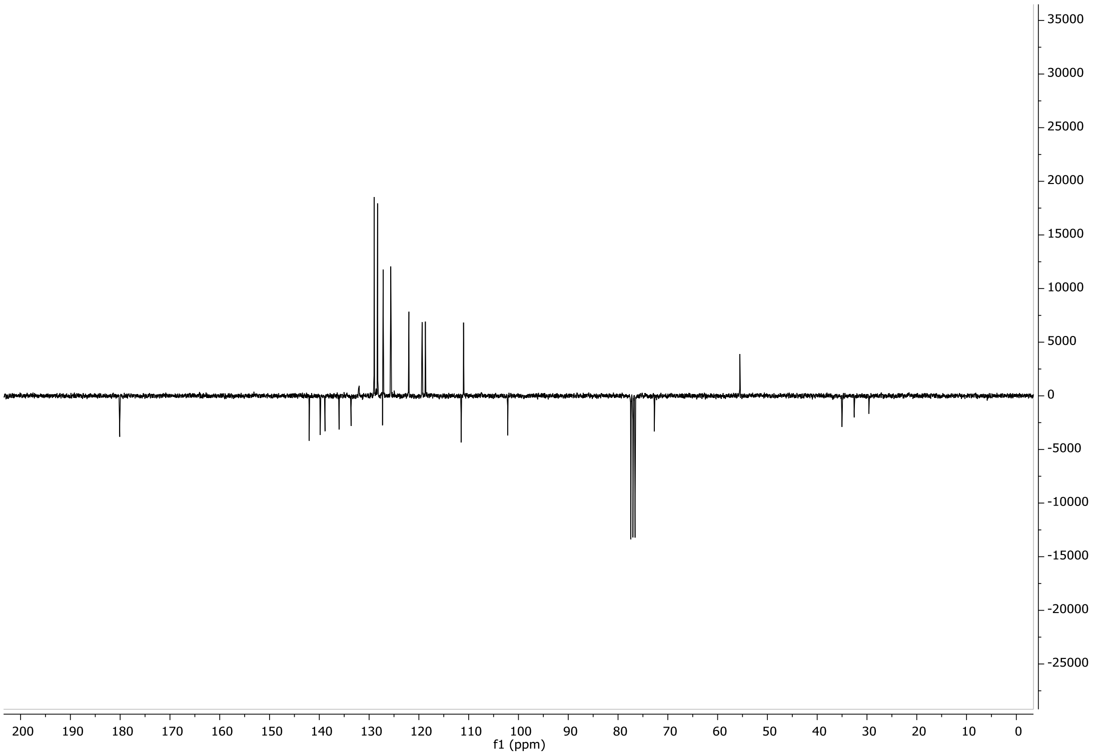

^{1}H NMR of compound **7o** ($CDCl_3$)

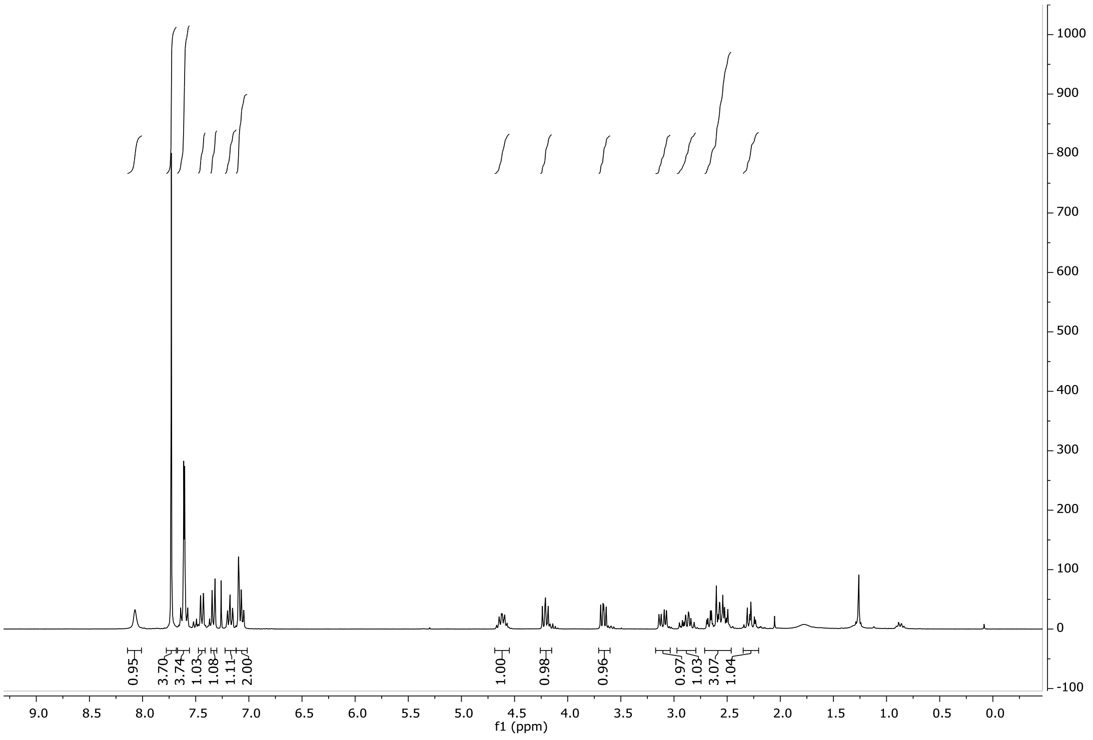

^{13}C NMR (APT) of compound **7o** ($CDCl_3$)

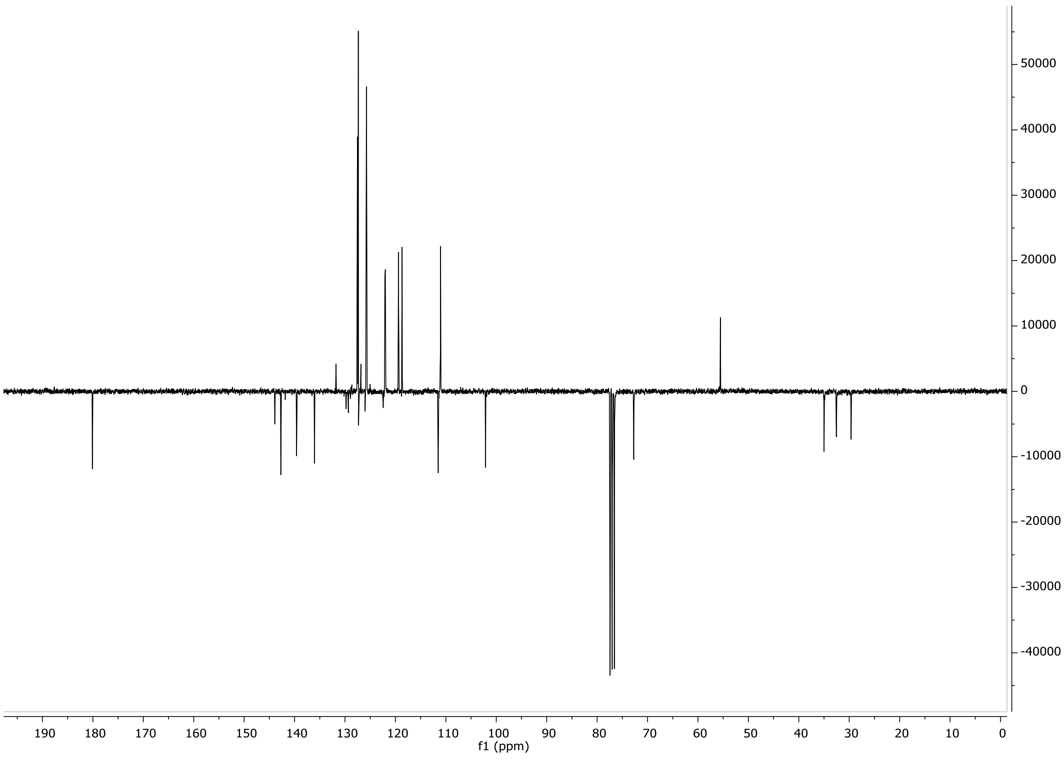

^{1}H NMR of compound **7p** (DMSO-d6)

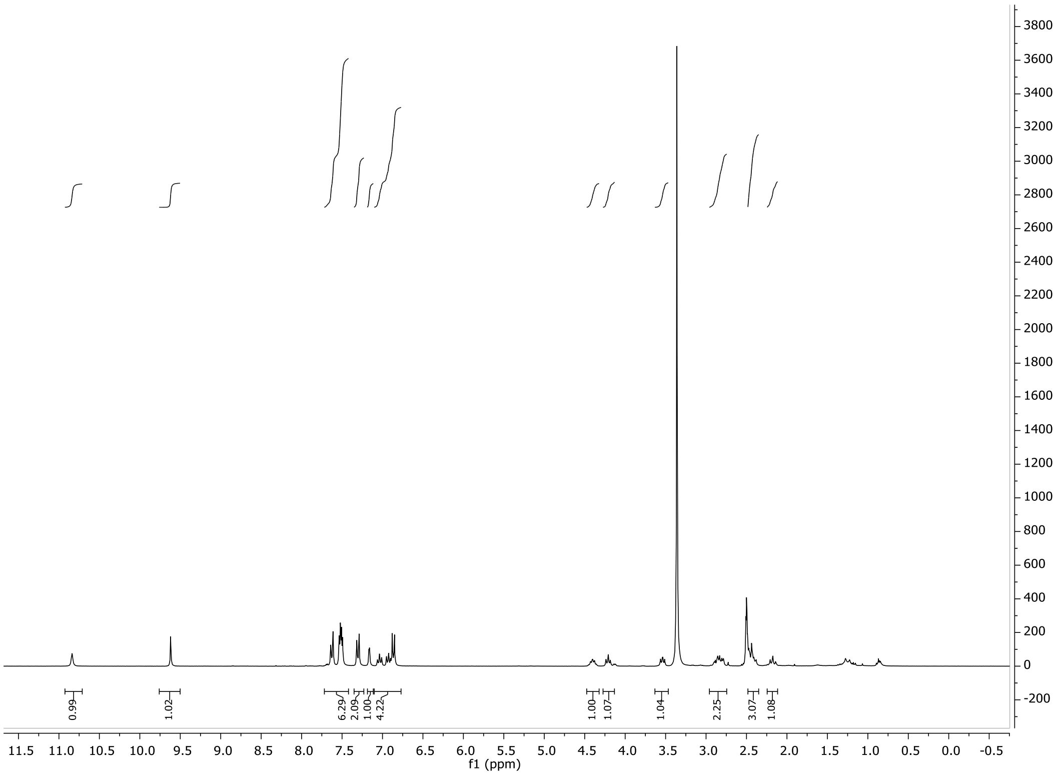

^{13}C NMR (APT) of compound **7p** (DMSO-d6)

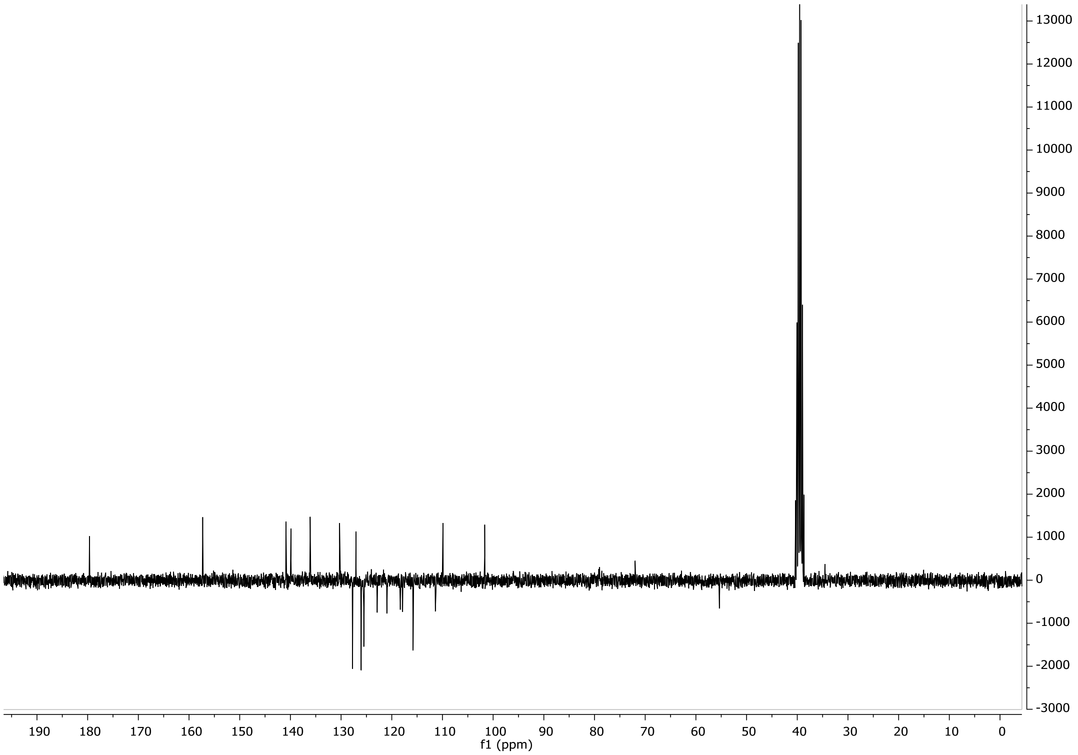

^{1}H NMR of compound **7q** ($CDCl_3$)

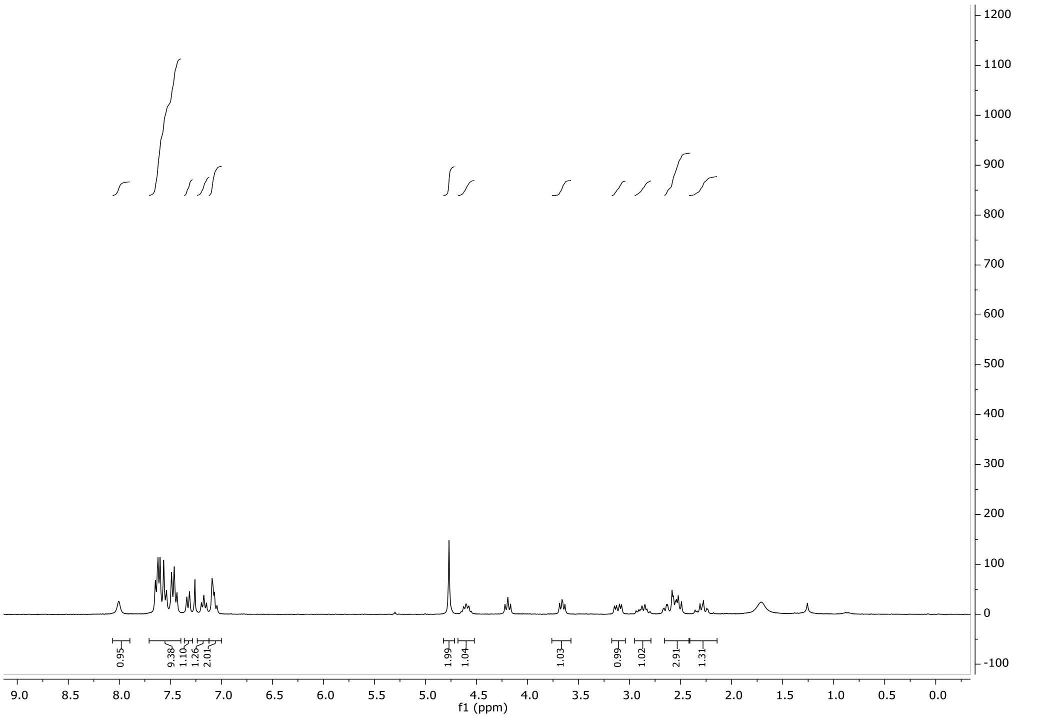

^{13}C NMR (APT) of compound **7q** ($CDCl_3$)

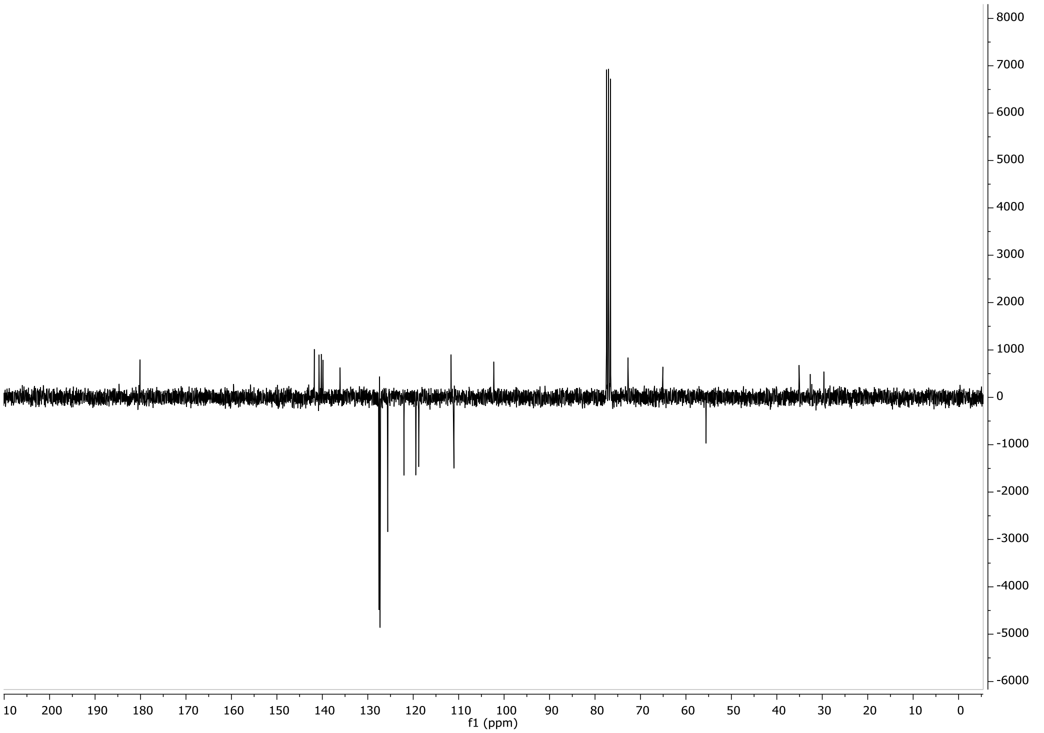

^{1}H NMR of compound **7r** ($CDCl_3$)

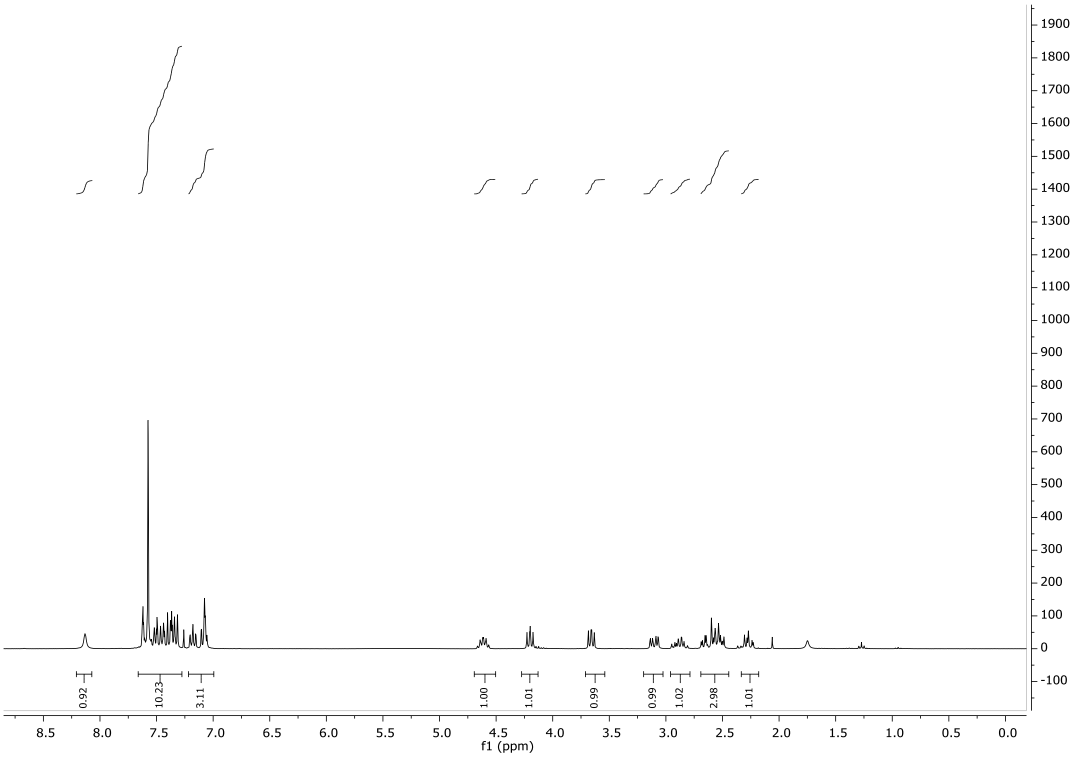

^{13}C NMR (APT) of compound **7r** ($CDCl_3$)

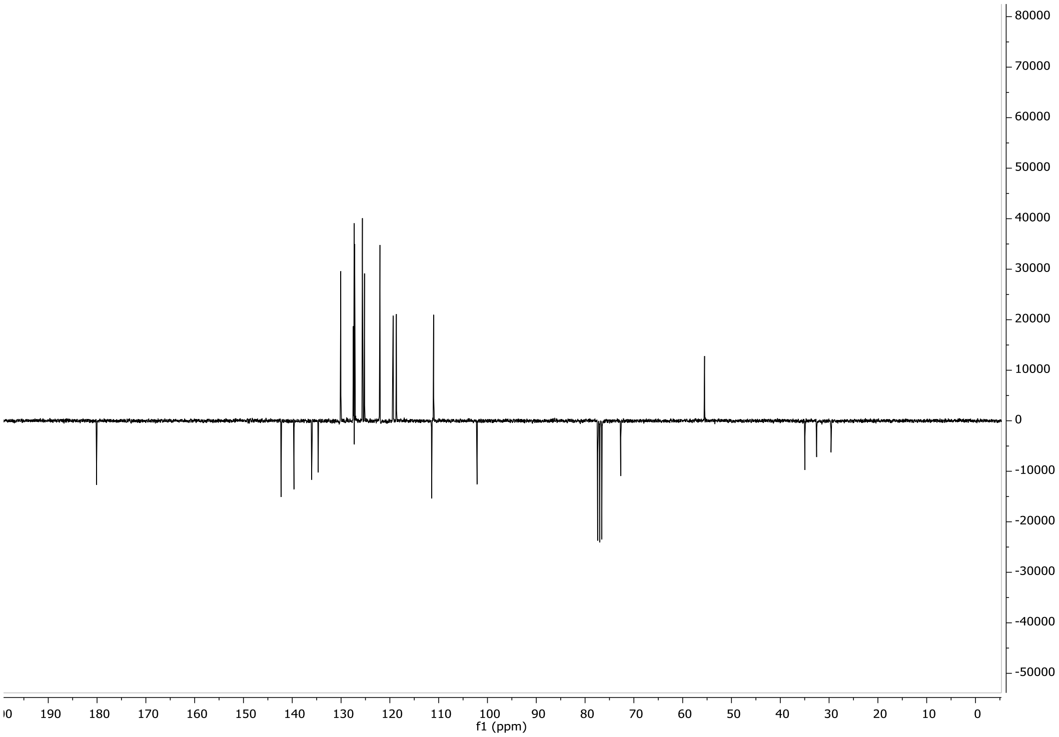

^{1}H NMR of compound **7s** ($CDCl_3$)

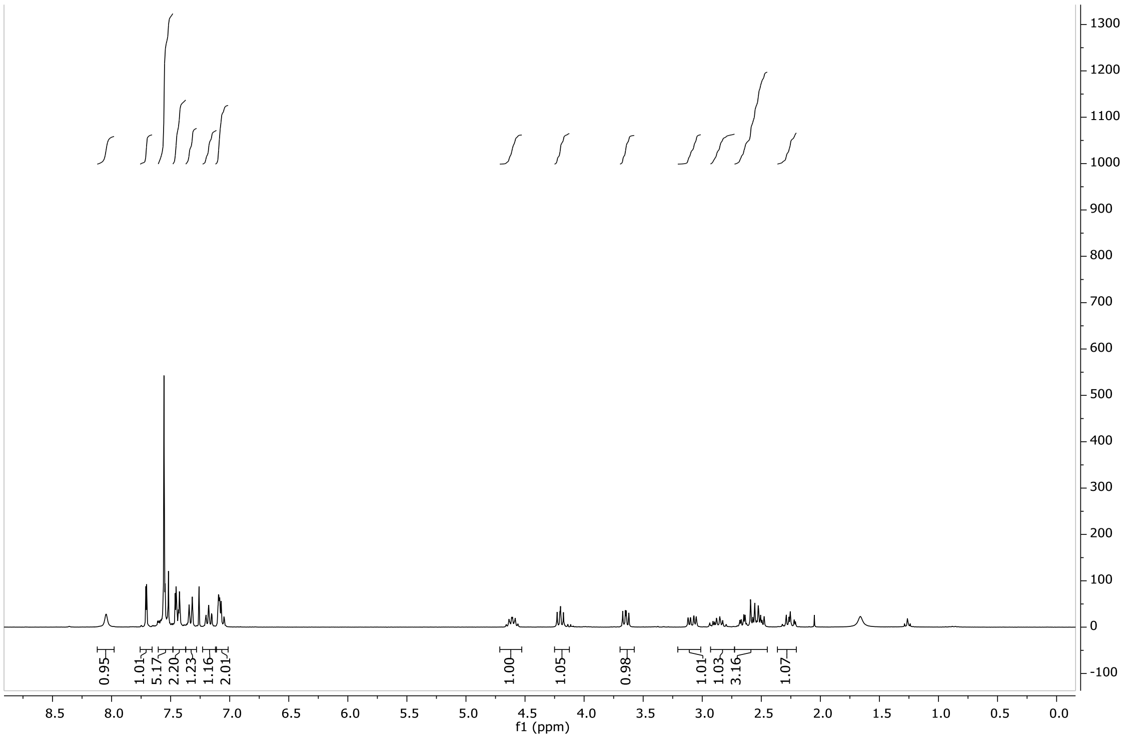

^{13}C NMR (APT) of compound **7s** (DMSO-d6)

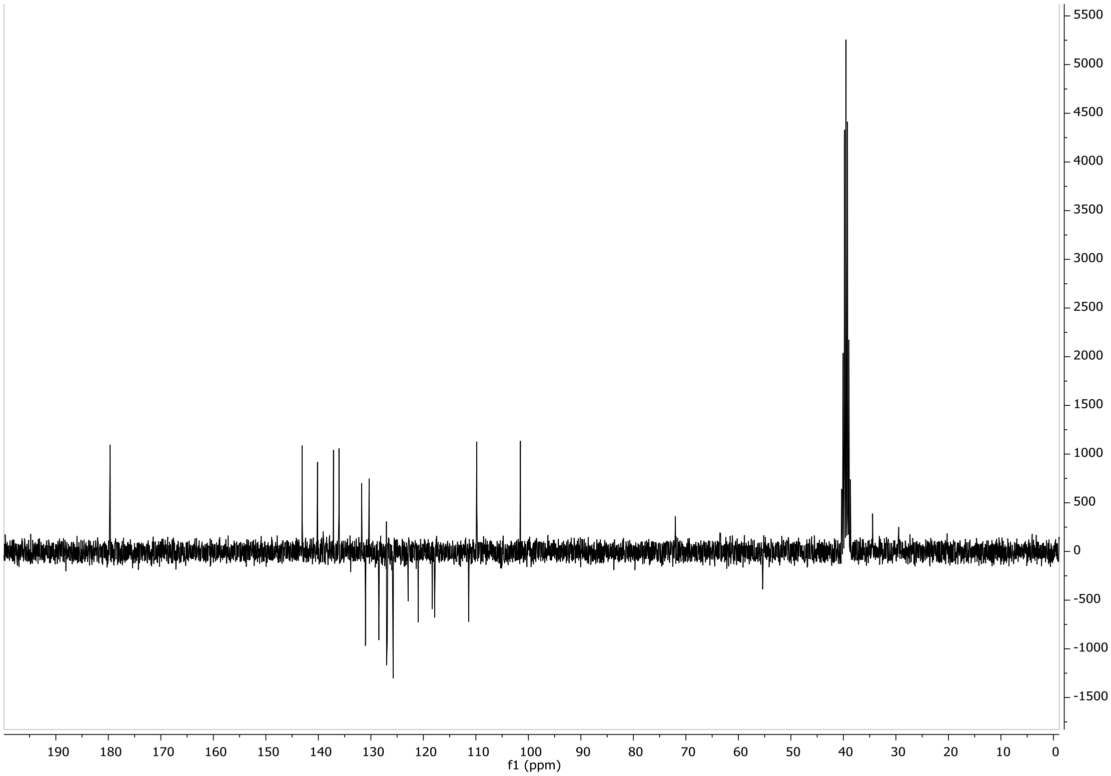

^{1}H NMR of compound **7t** ($CDCl_3$)

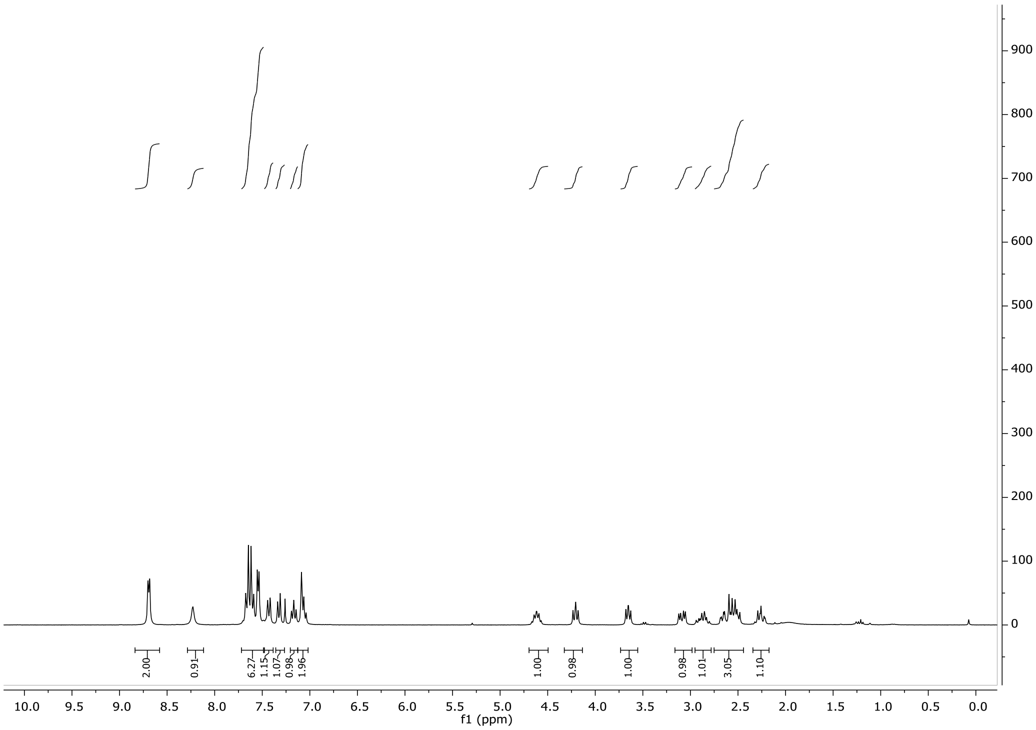

^{13}C NMR (APT) of compound **7t** ($CDCl_3$)

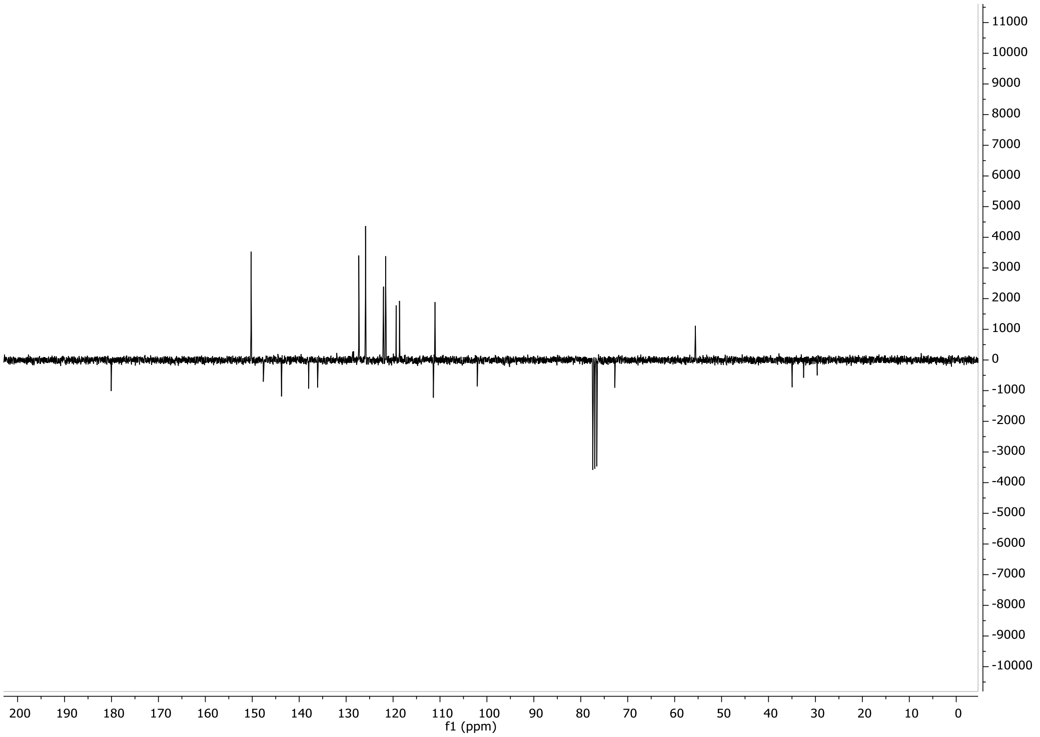

^{1}H NMR of compound **7u** ($CDCl_3$)

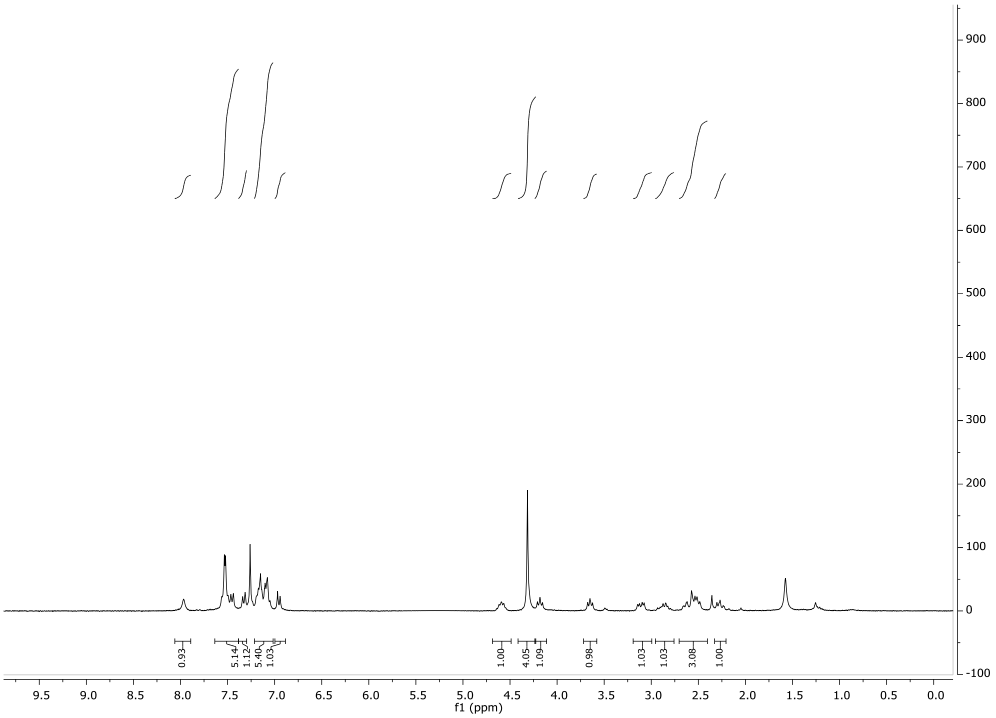

^{13}C NMR (APT) of compound **7u** ($CDCl_3$)

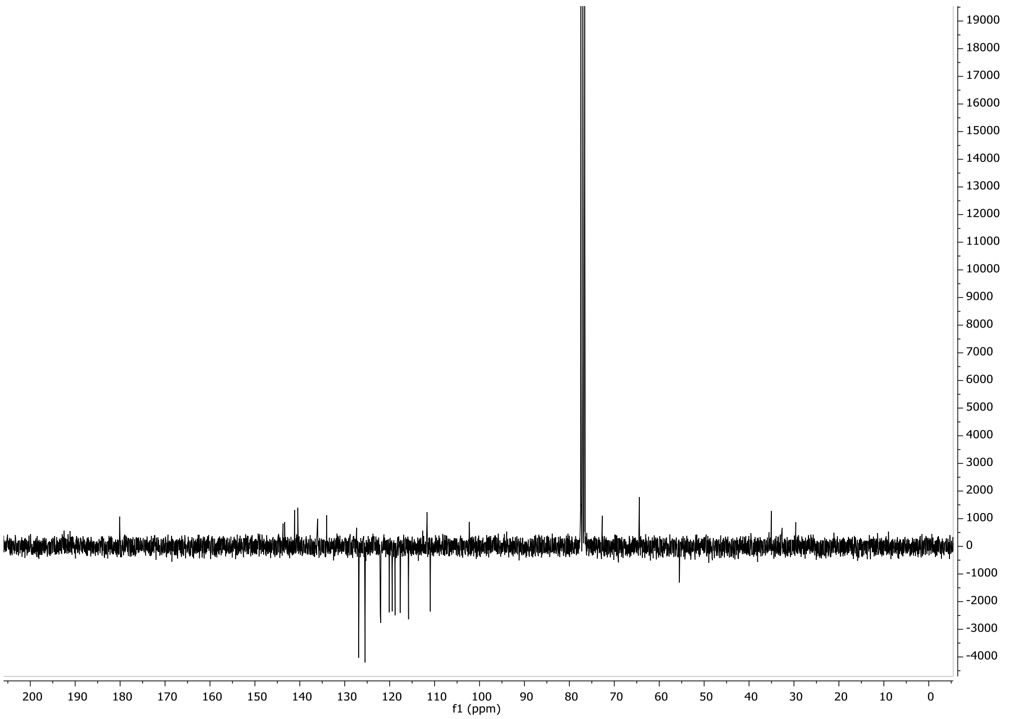

LC-MS data

Compound **7j**: retention time 6.7 min; purity 98.87%.

Page 1 of 1

Area % Report

Data File: C:\EZChrom Elite\Enterprise\Projects\João Pais\02-12-2020 12-48-02VB64A_TEST1.dat
Method: C:\EZChrom Elite\Enterprise\Projects\João Pais\Methods\QuickGrad.met
Acquired: 02-12-2020 12:48:10
Printed: 03-02-2021 11:35:24

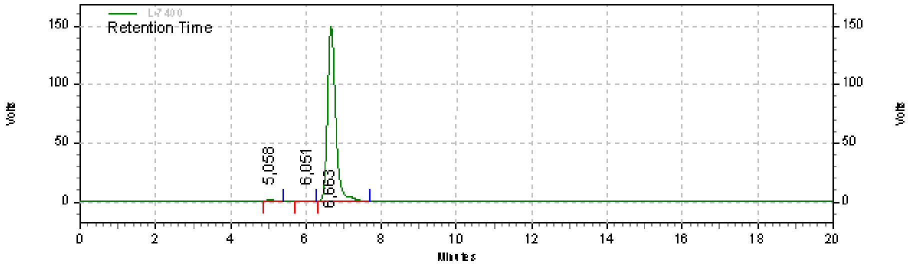

L-7400 Results

Retention Time	Area	Area %	Height	Height %
5,058	12777	0,57	1103	0,73
6,051	7473	0,34	591	0,39
6,663	2208164	99,09	148832	98,87
Totals	2228414	100,00	150526	100,00

MS spectrum of compound **7j** (m/z 381 [MH]+)

Note: red - blank spectrum (MeCN); green: sample spectrum.

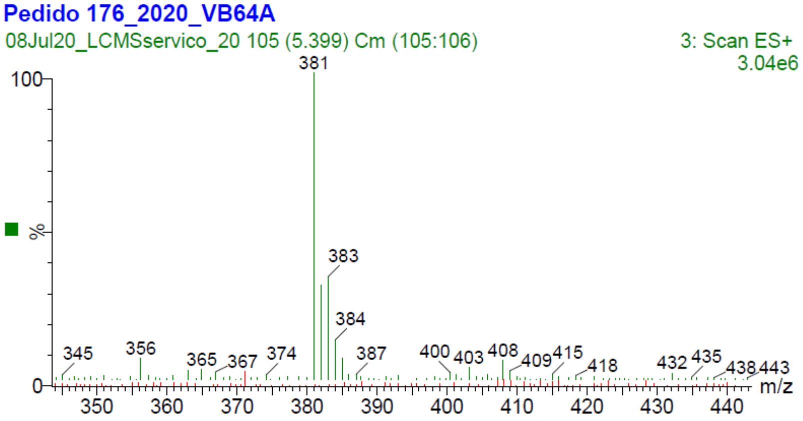

Compound **7j'**: retention time 6.1 min; purity 96.95%.

Area % Report

Data File:	C:\EZChrom Elite\Enterprise\Projects\João Pais\02-12-2020 18-08-05VB64B_2.dat
Method:	C:\EZChrom Elite\Enterprise\Projects\João Pais\Methods\QuickGrad.met
Acquired:	02-12-2020 18:09:57
Printed:	03-02-2021 11:42:24

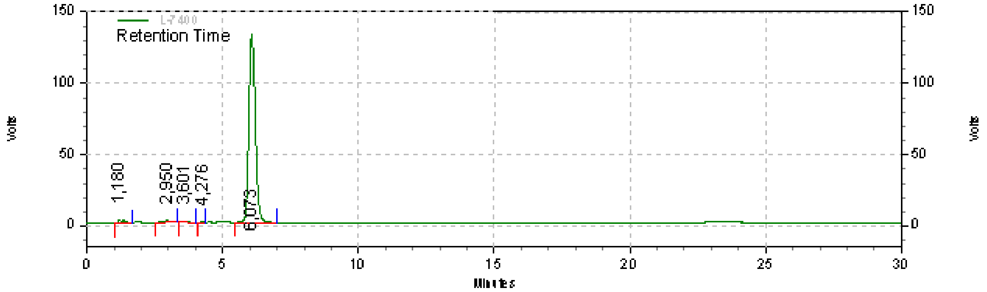

L-7400 Results

Retention Time	Area	Area %	Height	Height %
1,180	35763	1,61	2311	1,70
2,950	31670	1,43	1302	0,96
3,601	5936	0,27	399	0,29
4,276	1241	0,06	126	0,09
6,073	2140916	96,63	131678	96,95

MS spectrum of compound **7j'** (m/z 381 [MH]+ ion)

Note: red - blank spectrum (MeCN); green - sample spectrum.

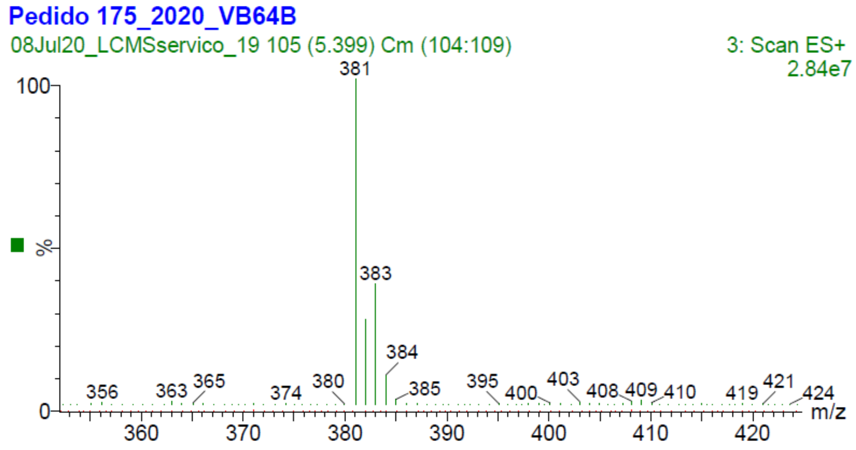

Compound **7h**: retention time 6.4 min; purity 96.62%.

Area % Report

Data File: C:\EZChrom Elite\Enterprise\Projects\João Pais\02-12-2020 11-54-19VB66RECRYSTILLIZED.dat
Method: C:\EZChrom Elite\Enterprise\Projects\João Pais\Methods\QuickGrad.met
Acquired: 02-12-2020 11:55:33
Printed: 03-02-2021 11:30:49

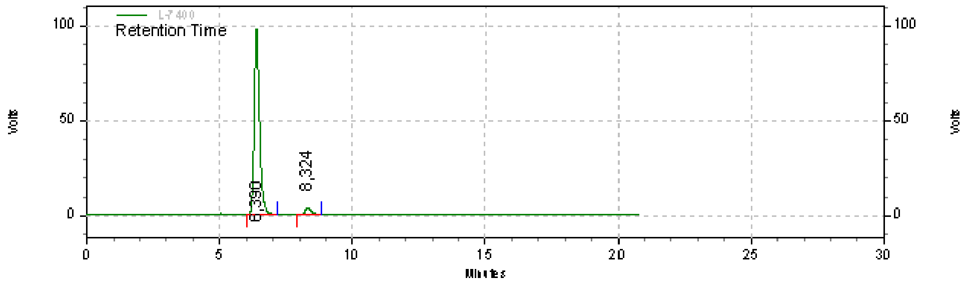

L-7400 Results

Retention Time	Area	Area %	Height	Height %
6,390	1370906	95,87	97763	96,62
8,324	58985	4,13	3424	3,38
Totals	1429891	100,00	101187	100,00

MS spectrum of compound **7h** (m/z 381 [MH]+ ion)

Note: red - blank spectrum (MeCN); green: sample spectrum.

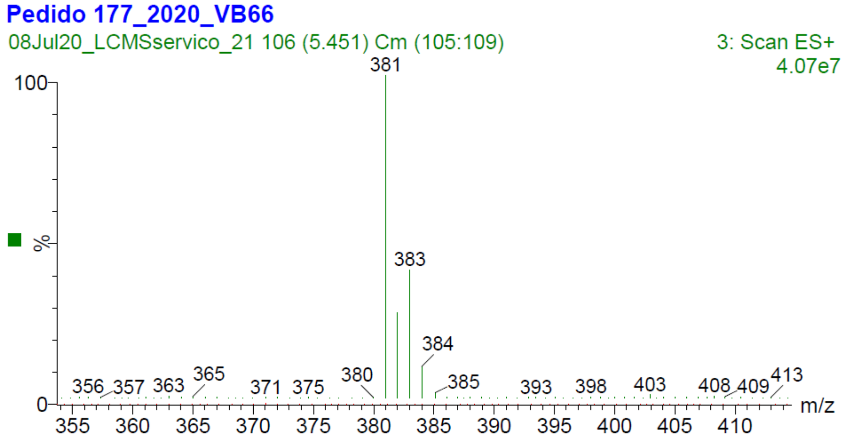

Supplement: Supplementary file 1 [file pharmaceuticals-14-00208-s001.pdf]
